# Evidence for the earliest structural use of wood at least 476,000 years ago

**DOI:** 10.1038/s41586-023-06557-9

**Published:** 2023-09-20

**Authors:** L. Barham, G. A. T. Duller, I. Candy, C. Scott, C. R. Cartwright, J. R. Peterson, C. Kabukcu, M. S. Chapot, F. Melia, V. Rots, N. George, N. Taipale, P. Gethin, P. Nkombwe

**Affiliations:** 1 Department of Archaeology, Classics & Egyptology, University of Liverpool, Liverpool, UK; 2 Department of Geography and Earth Sciences, Aberystwyth University, Aberystwyth, UK; 3 Department of Geography, Royal Holloway, University of London, Egham, UK; 4 Professor Elizabeth Slater Archaeological Research Laboratories, Department of Archaeology, Classics & Egyptology, University of Liverpool, Liverpool, UK; 5 Department of Scientific Research, The British Museum, London, UK; 6 University of Algarve, Interdisciplinary Center for Archaeology and Evolution of Human Behaviour (ICArEHB), Campus de Gambelas, Faro, Portugal; 7 TraceoLab/Prehistory, University of Liège, Liège, Belgium; 8 National Museums Board, Moto Moto Museum, Mbala, Zambia

**Keywords:** Archaeology, Sedimentology, Archaeology, Palaeoclimate, Limnology

## Abstract

Wood artefacts rarely survive from the Early Stone Age since they require exceptional conditions for preservation; consequently, we have limited information about when and how hominins used this basic raw material^1^. We report here on the earliest evidence for structural use of wood in the archaeological record. Waterlogged deposits at the archaeological site of Kalambo Falls, Zambia, dated by luminescence to at least 476 ± 23 kyr ago (ka), preserved two interlocking logs joined transversely by an intentionally cut notch. This construction has no known parallels in the African or Eurasian Palaeolithic. The earliest known wood artefact is a fragment of polished plank from the Acheulean site of Gesher Benot Ya’aqov, Israel, more than 780 ka (refs. ^2,3^). Wooden tools for foraging and hunting appear 400 ka in Europe^4–8^, China^9^ and possibly Africa^10^. At Kalambo we also recovered four wood tools from 390 ka to 324 ka, including a wedge, digging stick, cut log and notched branch. The finds show an unexpected early diversity of forms and the capacity to shape tree trunks into large combined structures. These new data not only extend the age range of woodworking in Africa but expand our understanding of the technical cognition of early hominins^11^, forcing re-examination of the use of trees in the history of technology^12,13^.

## Abstract (web summary)

Wooden artefacts from waterlogged deposits in Zambia dating back 477 ka indicate hitherto unknown sophistication in woodworking at an early date.

## Main

In the African context, indirect evidence for woodworking comes from use-wear traces and residues on Early Pleistocene stone tools in East Africa (Oldowan, Acheulean)^14–16^. Actual wood objects are found in Mid-Pleistocene waterlogged deposits in southern Africa with Acheulean and Middle Stone Age tools. At Kalambo Falls, wood was recovered from Acheulean horizons in the 1950s–1960s (sites A and B), but taphonomic processes removed evidence of intentional shaping from most pieces^17^. A wood chip and three objects with transverse notches raised the possibility of intentional modification. Attempts to date the wood gave minimum ages^18–20^. At Amanzi Springs, South Africa, a single stick with a possible chop mark was reported from waterlogged Acheulean deposits excavated in the 1960s^21^. The deposits were radiometrically dated (approximately 404–390 kyr), with wood found in recent excavations but without evidence of modification^10^. The earliest clearly modified wood object, collected in 1952 from spring deposits at Florisbad, South Africa, was associated with Middle Stone Age tools and hominin remains (*Homo helmei*)^22^. The object’s tip shows cutmarks and fine striations^23^, but its location relative to dated deposits is uncertain^24^.

**Fig. 1:**
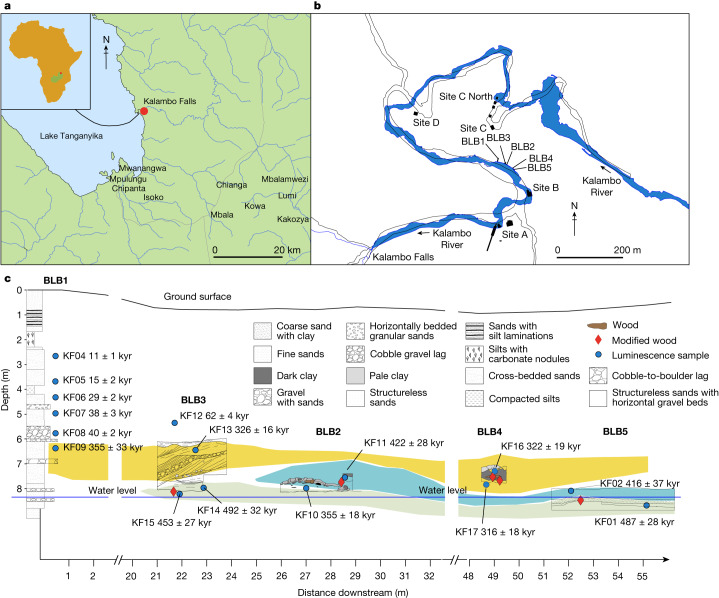
Location of Kalambo Falls archaeological site and excavated areas. **a**, Site location in south-central Africa. **b**, Course of the Kalambo River (in outline) from around 1956 to 2006 in relation to previous excavations at sites A, B, C, D and C North. Site BLB (2019) and excavation units BLB1, BLB2, BLB3, BLB4 and BLB5 are located along the current main channel (blue). **c**, Cross-section of the 2019 excavation units showing the location of 16 luminescence dating samples (KF01–KF17, dark blue circles, uncertainties (±) shown at 1 − *σ*) by unit. Unit BLB1 is a geological section of the full cliff exposure from ground surface to below water level. The three colour bands indicate clusters of pIR IRSL ages grouped by mean ages and standard error of the mean. The earliest wood objects (BLB5, BLB3) are in the lower green band with a mean age of 476 ± 23 kyr. The blue band has a mean age of 390 ± 25 kyr and incorporates the wood object in BLB2. The overlying yellow band has a mean age of 324 ± 15 kyr and incorporates wood objects in BLB4. Red diamonds indicate modified wood objects. Inset map data in **a**: Google Maps, Google 2021, INEGI; the map of Kalambo Falls was drawn using Arc-GIS Open Street Map. Map data in **b**: Google Imagery 2021 CNES/Airbus, Maxar Technologies; the 1960s river course, with site locations, was redrawn from figure 7.2 of ref. ^17^ with permission from Cambridge Univ. Press.

**Fig. 2:**
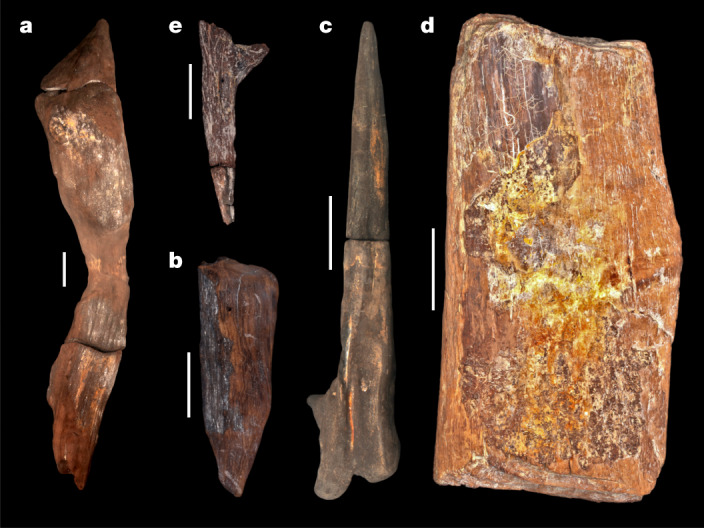
Modified wood tools from site BLB, Kalambo Falls, 2019. **a**, BLB5 structural element (object 1033). **b**, BLB3 ‘wedge’ (object 660). **c**, BLB2 ‘digging stick’ (object 219). **d**, BLB4 cut log. **e**, BLB4, tapered piece with single chop-mark. Scale bars, 10 cm.

Excavations at Kalambo Falls in 2019 recovered five modified wood objects at site BLB (Fig. 1b) from four areas (BLB2, BLB3, BLB4 and BLB5) in sediments above and below river level (Fig. 1c). A sixth object, from BLB3, showed no evidence of modification. Two objects were associated with Acheulean artefacts below the river (BLB3 and BLB5); three from contexts above river level, without stone tools (BLB2 and BLB4) (Fig. 2).

The Quaternary sequence is a 9-m-deep exposure above the Kalambo River (BLB1 is a geological section). Sediments are fluvial sands and gravels with occasional, discontinuous beds of fine sands, silts and clays with wood preserved in the lowermost 2 m (Methods). A permanently elevated water table has preserved wood and plant remains (Supplementary Information Section 1). The depositional sequence is typical of a high- to moderate-energy sandbed river that underwent lateral migration. The sands are dominated by a lower unit of horizontal bedding and an upper unit of planar/trough cross-bedding. Upper and lower sand units are separated by fine sands, silts and clays with plant material deposited in still water after the river migrated/avulsed elsewhere in the floodplain. Wood is deposited in this environment either through anthropogenic emplacement, or naturally transported in the flow, and snagged on sand bedforms (Supplementary Information Section 1).

Dating is based on 16 sand samples collected for luminescence analyses from deposits bracketing key finds including those containing wood. Younger samples are dated using single-grain quartz optically stimulated luminescence (OSL) and older samples by postinfrared infrared stimulated luminescence (pIR IRSL) from potassium-rich feldspars (Methods and Supplementary Information Section 2). The pIR IRSL approach used extensively in recent years^25,26^ does not suffer the problems that can generate large uncertainties associated with thermally transferred OSL (TT-OSL), as seen at Site C North (Fig. 1b)^20^. The new ages (Extended Data Table 1) are in stratigraphic order (1*σ*) (the only exception being KF10 and KF11), forming three clusters based on mean ages (and error of the mean) (Fig. 1c). The earliest cluster (476 ± 23 kyr) incorporates wood in deposits below river level (BLB3, BLB5). The intermediate cluster (390 ± 25 kyr) encompasses one wood tool in BLB2 found above river level. The upper cluster (324 ± 15 kyr) brackets two wood objects in BLB4, above river level. No wood was found higher up the sequence.

The wood was sampled for identification to species level (Methods and Supplementary Information Section 3)^27^ and for radiocarbon dating, to assess for an intrusive origin; all dates were infinite (more than 50 kyr ago (ka), T. Higham personal communication). Infrared spectroscopy shows partial mineralization of the wood (silica) (Methods and Supplementary Information Section 5). Surface modifications were identified on photographically generated models (Methods). The need to keep the wood wet made standard photography, structured light scanning and microscopy impractical because of high surface reflectance and pooling of water in key features, obstacles overcome by photogrammetry and reflectance transformation imaging (RTI) on the submerged wood. Additional photographs were taken after brief episodes of drying.

Manufacture marks and use history are described using standardized terminology^1^ with interpretations supported by replication experiments (Methods and Supplementary Information Sections 6 and 7). Functional interpretations are drawn from wood artefacts in waterlogged Holocene sites in Zambia^28^, the UK^29,30^ and ethnographic sources (Methods and Supplementary Information Section 8)^31,32^. Individual pieces are described by excavation block from oldest to youngest.

**Fig. 3:**
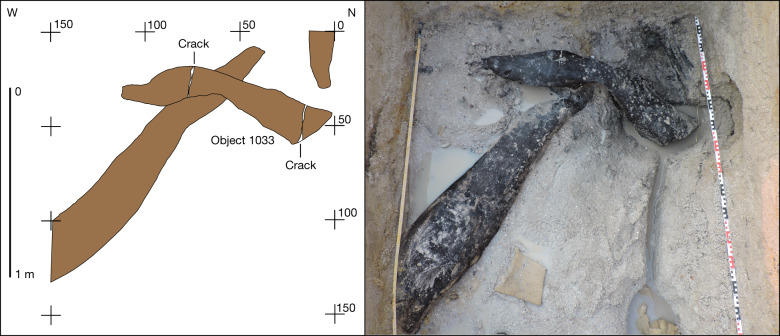
Structural unit formed by two overlapping logs in BLB5. The underlying log passes through a central notch cut into the upper log (object 1033) and extends into the section. Plan view of the unit (left) and during excavation (right). The numbers refer to the distance in centimetres.

**Fig. 4:**
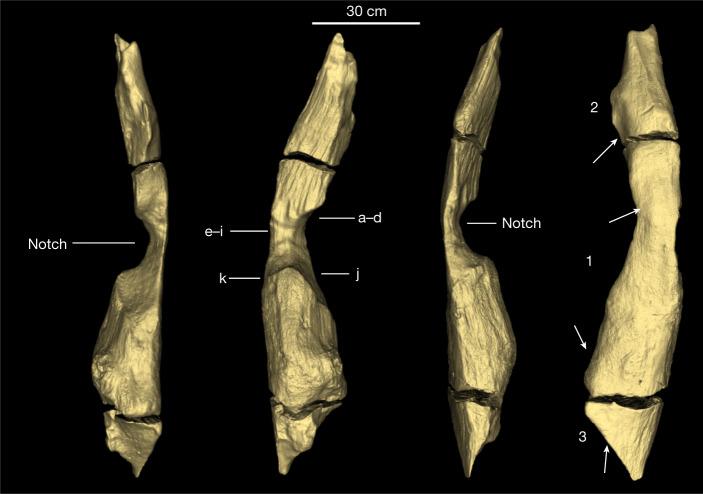
Annotated images of the BLB5 upper log (object 1033) showing areas of intentional modification. From left to right, the location of the central notch in profile, shaping marks in and on the margins of the notch (a–k), the notch in profile from the opposite side. The image on the right shows the upper surface of the log, and the three parts of the log (1–3) separated by cracks. White arrows indicate locations of shaping facets on the sides and upper surface of the log.

In BLB5, one modified object was recovered from basal sands containing flake tools, cleavers and handaxes. Object 1033 is a log (*Combretum zeyheri*) 141.3 cm long × 25.6 cm, with tapering ends in three parts, which overlies a larger treetrunk at a 75° angle (Fig. 3a,b). The area of overlap is a U-shaped notch 13.2 cm long by 11.4 cm wide, transverse to the long axis (Fig. 4). The underlying trunk, also modified, and left in situ, passes through the notch (Fig. 3a).

**Extended Data Fig. 1:**
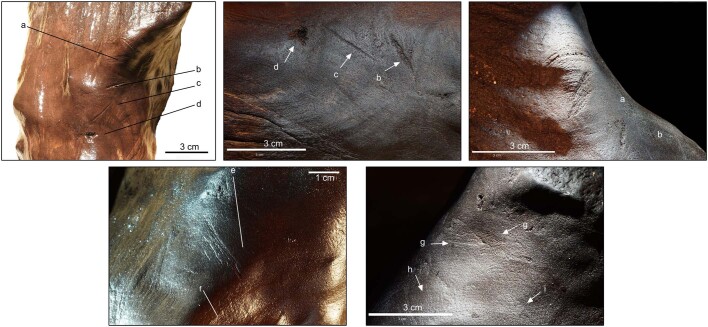
Location of modification marks on object 1033 (BLB5). Two areas of modification are identified in the Upper and Lower parts of the notch (Fig. 2a; Fig. 4). Upper: 1a shows distribution of marks ‘a–d’; 1b provides detail of intersecting striations ‘a’ and facet ‘b’. Image 1c shows facets ‘b’ and ‘c’ and impact pit ‘d’ (scale = 3 cm). Lower: 2a shows striation clusters ‘e’ and ‘f’ (scale = 3 cm). 2b. Intersecting striations at ‘g’; parallel striations at ‘h’; wavy bands of flat striations at ‘i’ (scale = 3 cm).

Bark (on parts 1 and 2) and a knot (part 1) occur near sapwood on object 1033. The notch (part 1) exposes tangential and radial longitudinal sections, with sapwood missing. Evidence of chopping and scraping occurs on the upper surface of the notch (Fig. 4, areas a–d, and Extended Data Fig. 1) and on the lower surface (Fig. 4, areas e–k, and Extended Data Fig. 1). Area a has two transverse intersecting sets of scraping marks with superimposed parallel linear striations (maximum length 12.5 mm with V-shaped cross-sections, maximum width 3.2 mm). Area b has two linear transverse entry facets (18.8 mm long × 1.6 mm wide). Area c is a single facet with clear entry and stop marks (24 mm long × 1.4 mm wide). Area d is an irregular pit (9.7 mm long × 6.8 mm wide) (Extended Data Fig. 1).

**Extended Data Fig. 2:**
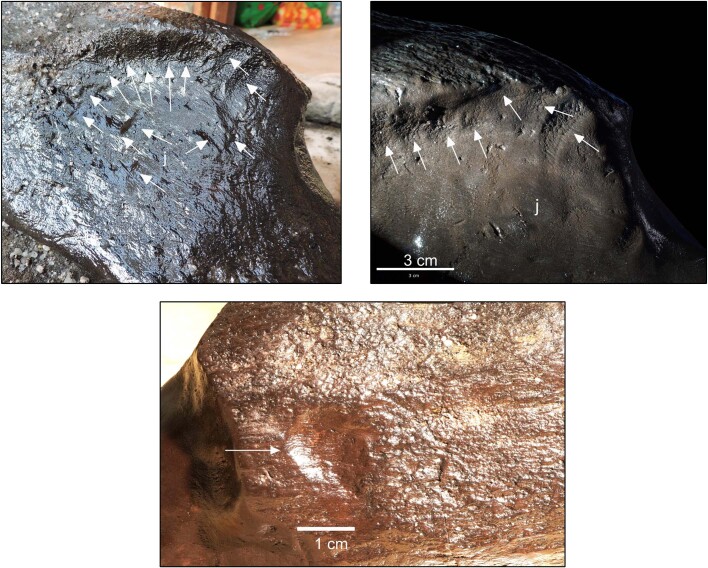
Location of modification marks on Part 1, object 1033. Upper left image shows area ‘j’ on the lower part of the notch seen on excavation with distinctive and extensive convex chop marks on the surface and back edge where it intersects the upper surface of the log. Right image highlights the loss of facet definition in area ‘j’ during storage. (Scale = 3 cm). The fine surface detail is gone leaving only the deepest scarring. Lower image is ‘k’, a single convex chop-mark on lower side of the notch, cutting into the surface bark. (Scale = 1 cm).

The lower areas e–h are sets of multiple intersecting linear striations with V-shaped cross-sections, transverse to the long axis (maximum length 17.3 mm × 1 mm width) (Extended Data Fig. 1). Area i striations are sinuous, parallel (42.8 mm long) and transverse to the grain with indeterminate cross-sections. A small entry facet cuts area e. Area j is defined by multiple convex-shaping facets (27 mm maximum length × 14 mm width) intersecting the bark surface and forming the flat surface below (Extended Data Fig. 2). All facets have lost definition during storage. Area k (Extended Data Fig. 2) is a single convex facet (41.8 mm × 39.8 mm).

**Extended Data Fig. 3:**
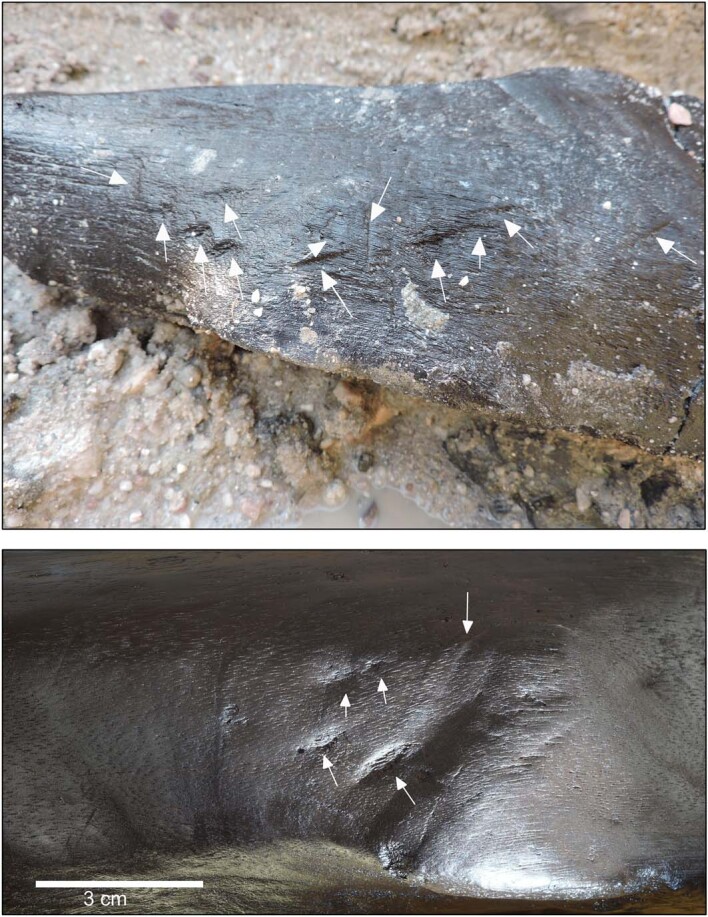
Shaping marks on Part 2 of object 1033. Upper image shows shaping marks on the tapered side and surface of Part 2 as seen on excavation (Fig. 4). Lower image shows loss of definition during storage. The arrows in the Lower image correspond with the area to the left of centre on the Upper image.

**Extended Data Fig. 4:**
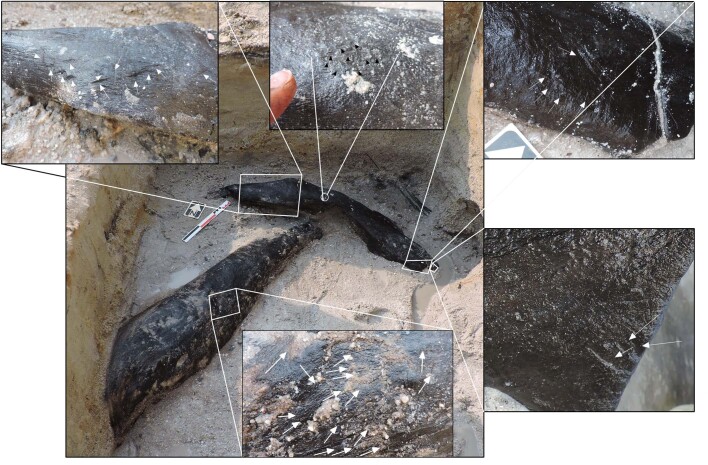
Shaping marks on the upper surfaces of object 1033 and on the underlying treetrunk (Fig. 3). Clockwise, from left upper left; chop marks on Part 2 (Extended Data Fig. 3); cluster of small convex hewing marks on Part 1, near Part 2 (Fig. 4); cutmark (upper arrow) and small facets (lower arrows) on Part 1 near Part 3 (Fig. 4); intercutting chop marks on the upper right edge of the Part 3 taper (Fig. 4); underlying log midsection, intersecting cutmarks transverse to the grain (bold arrow, upper left, indicating direction of grain). Marks on underlying treetrunk interpreted as result of scraping, perhaps from debarking.

On the left side and upper surface of part 2 (Fig. 4), at least 12 convex-shaping facets and some intercutting are clearly visible on excavation (Extended Data Fig. 3). Five remain visible after storage as dimpling (Extended Data Fig. 3), resembling faceting marks on waterlogged wood from Star Carr, UK^29^. Consecutive facets define a portion of the upper tapered edge of part 3 (Extended Data Fig. 4). Two clusters of small convex facets occur on the upper surface of part 1 (Fig. 4 and Extended Data Fig. 4).

**Extended Data Fig. 5:**
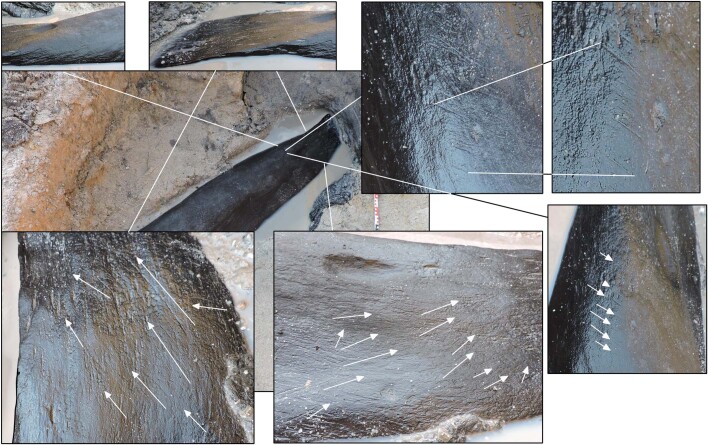
Shaping marks on the treetrunk underlying object 1033 notch, BLB5. Surface modifications on the treetrunk (Fig. 3) beneath and just beyond the notch (Fig. 3., Extended Data Fig. 4). Images described counter clockwise: white arrows highlight direction of striations, some V-shaped in profile, cutting across the grain from the edge toward the middle of the surface; a set of parallel transverse striations/cutmarks, with V-shaped profiles (white arrows) along ridge descending to a flat tapered surface; inset detail of the striations on the ridge crest; overview of the set of striations along the ridge; profile view of the flattened surface below the ridge; profile view of the ridge and break of slope onto the flat tapering surface which extends beyond the notch; plan view of the flat tapering end with transverse striations and cutmarks with V-shaped profiles (white arrows) interpreted as shaping marks.

Surface modification occurs on the underlying trunk at its midpoint and along the narrowed end that passes through and beyond the notch (Fig. 3 and Extended Data Fig. 5). At the midpoint, a small area (approximately 10 cm × 5 cm) preserves multiple short parallel striations transverse to the grain, with V-shaped sections, indicative of scraping^1^ (Extended Data Fig. 1). The log’s end tapers in plan and profile view with two sets of shaping marks visible. Parallel linear V-shaped cutmarks, transverse to the grain occur at the start of a break in slope, increasing in depth and length downslope (Extended Data Fig. 5). A second set of marks occurs on the flat surface which emerges beyond the notch and continues into the section (Extended Data Fig. 5). Multiple linear and intersecting groups of V-shaped fine striations extend across this surface at acute and right angles to the grain.

We interpret the notch as intentional, made by scraping and adzing^1^ to create a join between the log and trunk, forming a construction of two connected parts. Infrared spectroscopy (Supplementary Information Section 5) provides indeterminate evidence for use of fire in shaping the notch. Clark^17^ described a similar find, from the Acheulean in Site B, of comparable length (165 cm long) with a “wide and deepish groove” transverse to the long axis, with tapered ends. He interpreted the groove as anthropogenic and suggested it was part of a structure. The excavation of two interlocking logs in BLB5, with shaped ends on both objects supports this interpretation. We know of no comparable construction in the early archaeological record.

**Extended Data Fig. 6:**
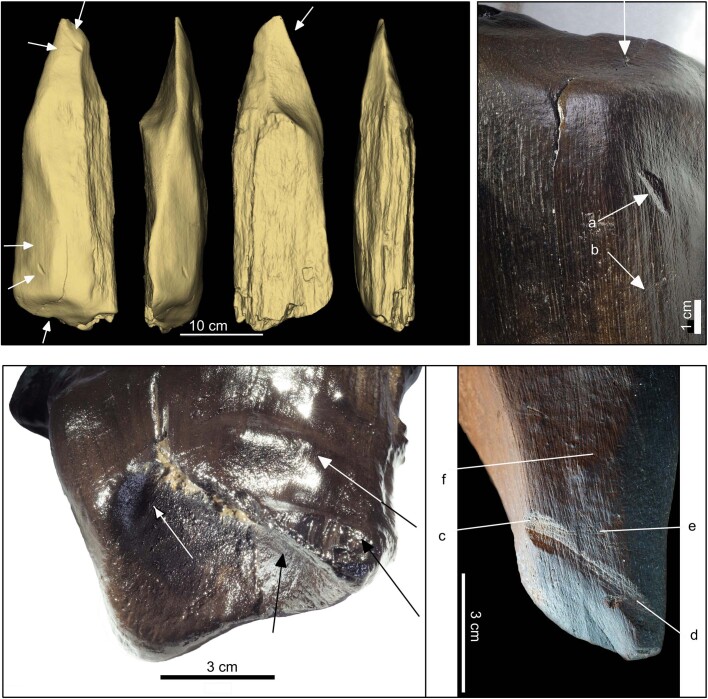
Shaping and possible use marks on object 660, BLB3. Surface modifications on ‘wedge’ (Fig. 2b) shown clockwise: white arrows indicating location of modifications on base and either side of the tip; basal crack (top arrow) and side split, facet ‘a’ intercepts a striation, possible use damage, and ‘b’ indicates a set of faint transverse striations; ‘c’ and ‘d’ intersecting facets above tip, ‘e’ and ‘f’ are sets of parallel faint transvers striations; tip face with convex step terminations, possibly from use.

In BLB3, two wood objects were found among plant material beside a large unmodified treetrunk. Flake tools, cleavers, handaxes and core axes occur in this deposit. Object 661 (*Ficus* spp.) is a V-shaped piece of branch, lenticular in cross-section with no visible shaping marks (not illustrated). Object 660 (*Kigelia africana*), 36.2 cm long, rounded on one side with outer bark on both surfaces, tapers to an offset point cut 60° across the long axis (Fig. 2b and Extended Data Fig. 6). The base is convex with a deep crack initiated in the pith, extending partially down each side (Extended Data Fig 6). Two modified areas occur below and transverse to the grain: a facet entry heel^33^ (a) and faint parallel linear striations of indeterminate profile (b) (Extended Data Fig. 6). Above the tip are four sets of marks: two intercutting convex facets with internal linear V-shaped cross-sections (c and d) and two sets of parallel striations (V-shaped) transverse to the grain (e and f) (Extended Data Fig. 6). The tip ends in a blunt break, subsequently rounded, its other side having three faint step fractures transverse to the grain obscured by the pitted surface (Extended Data Fig. 6).

We interpret this as an intentionally shaped object altered by high-impact compression down its long axis. Possible uses include a wedge, an incompletely processed firewood and a portable work base (Methods). Minimally modified bark wedges are known ethnographically^31^ and short-shaped roundwood wedges from the Mesolithic (Star Carr, UK)^30^. This unusual object has no parallel in the Middle Pleistocene record.

**Extended Data Fig. 7:**
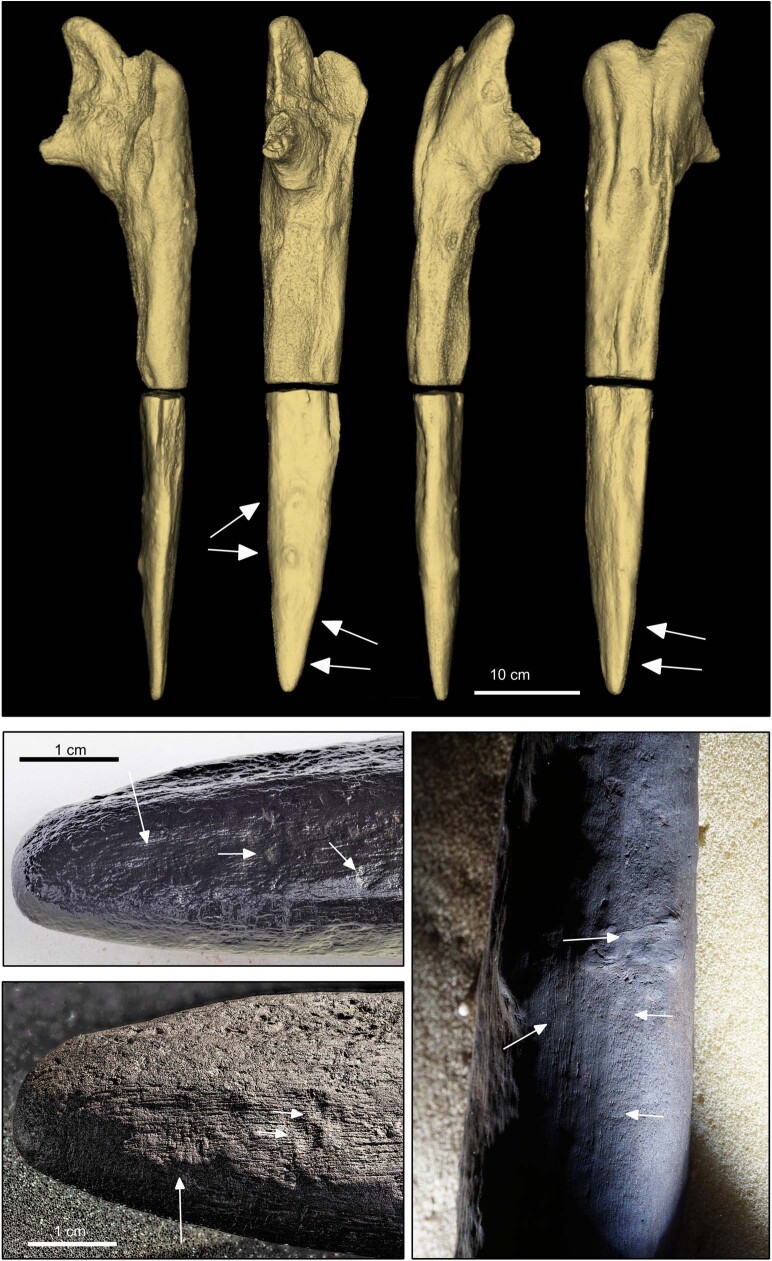
Shaping marks on object 219, BLB2 (Fig. 2c). Upper image shows all views of the object with white arrows indicating location of marks on either side of the tip and above the tip. Both sides of the tip (two images on left) have a faint set of transverse striations and two or more small facets away from the tip. (Scale = 1 cm.) The vertical image to the right shows four groups of faint parallel transverse striations in an area with two knots, no scale. The object is interpreted as a digging-stick.

In BLB2, object 219 (*K. africana*) was recovered in two refitting parts, one preserved in sediment, the other in the river directly below (Extended Data Fig. 7). The combined length is 62.4 cm, a maximum breadth at the base 11.9 cm, midsection breadth 6.1 cm and 1.3 cm at the offset tip (Fig. 2c). No bark occurs on the object; the upper surface is concave from base to tip (Extended Data Fig. 7). Faint linear striations, with indeterminate cross-sections, cut across the grain near the tip on both sides along with small (less than 10 mm) indistinct convex facets (Extended Data Fig. 7). The obverse surface preserves four areas with linear striations above the tip, including around two knots, with indeterminate cross-sections (Extended Data Fig. 7). Tip rounding may be from exposure to the river or use. Mid-Holocene waterlogged deposits at Gwisho, central Zambia preserved four digging sticks. Only one has small facets (less than 20 mm) on both sides of the tip^28^. The Gwisho tools are short by comparison with those used historically by Kalahari foragers^32^ but similar in length to object 219. We interpret object 219 as a digging stick based on its morphology and presence of shaping facets^33,34^.

**Extended Data Fig. 8:**
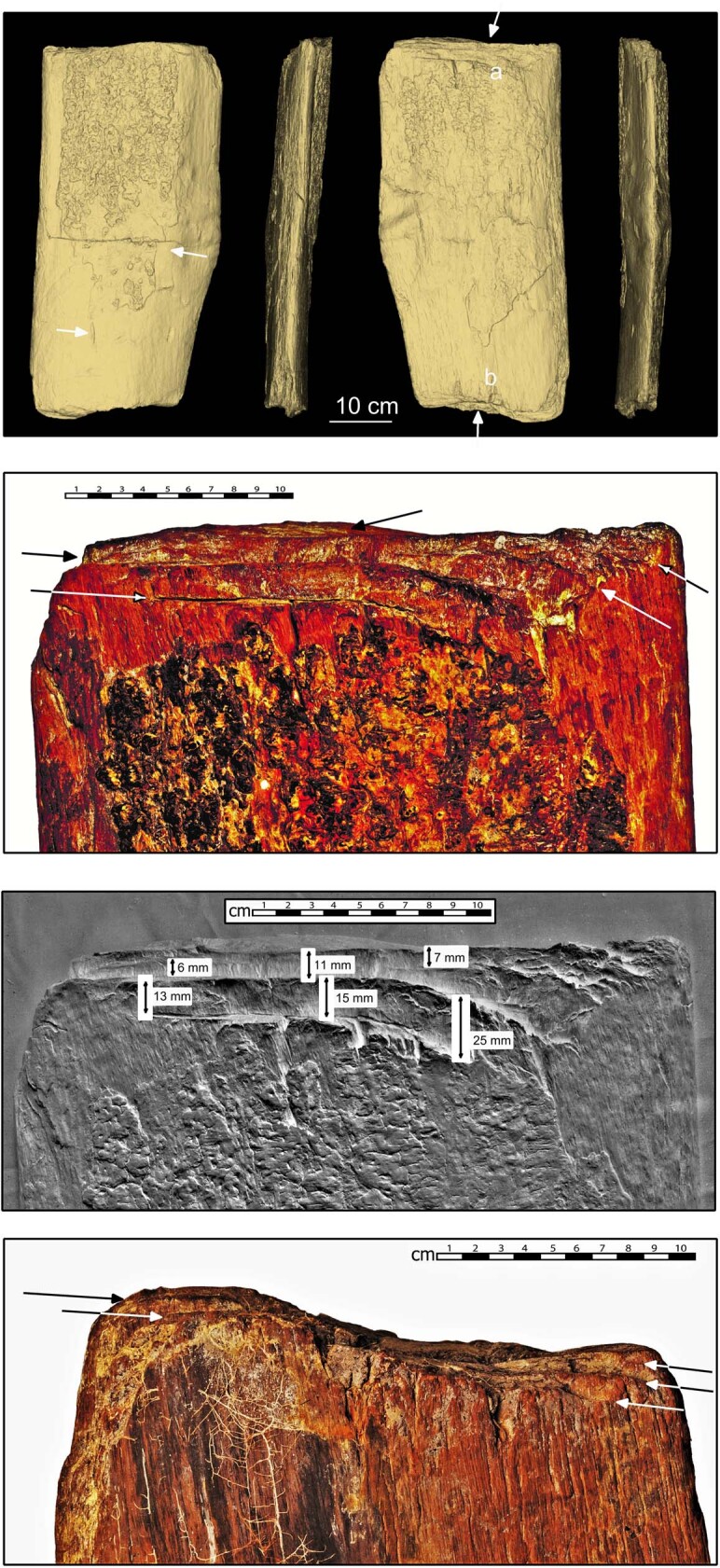
Chop marks on ‘cut log’, BLB4 (Fig. 2d). Upper image shows all views of the object and location of modifications (white arrows) on the ends, ‘a’ and ‘b’, two marks on the underlying surface and the preservation of bark on both surfaces. Image below is ‘a’ with its distinctive stepped chop marks (black arrows), and underlying grey image (denatured RTI) shows the width of the step between the first and second chop-mark expanding, left to right, from 13 to 25 mm. The deep, slightly convex cut of the first chop is visible. The underly step expands left to right from 6 mm to 11 mm. A third step starts with a width of 7 mm. Bottom image is end ‘b’ with black arrows indicating location of chop marks.

In BLB4 two wood objects were preserved in clays, one above the other (Fig. 2d,e). No stone tools were found. The upper object (*Combretum zeyheri*) is rectangular (59.24 cm × 29.34 cm × 7.7 cm) with bark traces and sapwood exposed on longitudinal surfaces (Extended Data Fig. 8). Evidence of radial cellular compression indicates flattening by sediment overburden. The ends bear distinct chop marks across the long axis (Fig. 2d; Extended Data Fig. 8). The obverse surface has one entry facet parallel to the grain and a set of transverse parallel linear striations of indeterminate profile (Extended Data Fig. 8).

Well-preserved chop marks occur at end a where they descend in four steps, with a V-shaped profile, maximum depth of 2.3 cm, and the top step expanding in width from 13 mm to 25 mm (Extended Data Fig. 8). Stepped cuts occur across end b (Extended Data Fig. 8). The length of cuts and slight curvature suggest a broad, sharp, cleaver-like edge, the depth indicating applied direct force, either handheld or hafted (Methods and Supplementary Information Section 7). We interpret this object as a portion of treetrunk cut to size, indicating capacity to work wood at a large scale.

**Extended Data Fig. 9:**
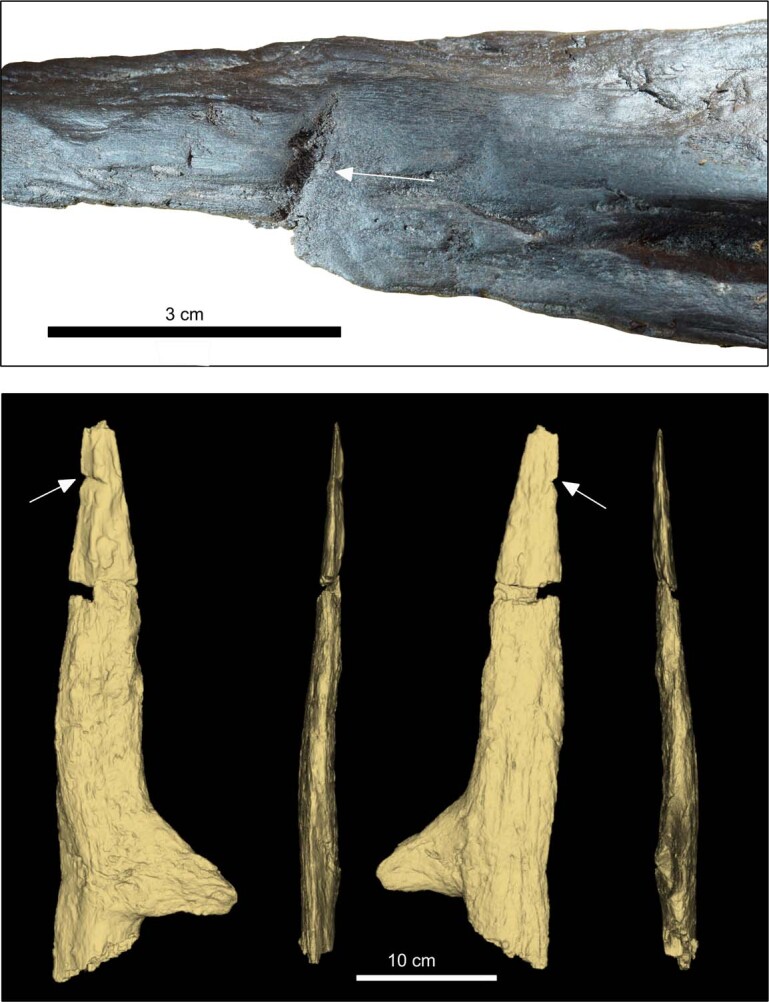
Images of split branch, BLB4 (‘notched stick’; Fig. 2e). Above, close-up of the facet/chop-mark with arrow indicating direction of initiation. Below, all views of split branch with chop (white arrow) and close-up of the entry facet. The object was found cracked in situ.

The lower object (*C. zeyheri*) (Fig. 2e) is from branch wood, with a side-branch attachment, split centrally, length 37.9 cm, tapering from the base (12.3 cm wide) to a broken tip (2.1 cm wide) (Extended Data Fig. 9 and Supplementary Information Section 4). A single chop mark above the tip is transverse to the grain, V-shaped in profile with an irregular termination. We were unable to interpret this object.

The recovery of modified wood with an exceptional level of preservation at Kalambo Falls places woodworking firmly in the Mid-Pleistocene of Africa. Our understanding of innovation among early hominins has been largely inferred from lithic artefacts^35^. Wood from tree trunks enabled humans to construct large objects such as platform foundations that necessitated tools for felling and hewing. The large cutting tools of the Acheulean fulfilled these roles in the long co-evolution of stone- and woodworking^13–15^. Hominin use of the Kalambo River basin coincides with an extended period of forest cover (470–274 kyr)^36,37^. Availability of forest resources and a permanent elevated water table created a habitat conducive to sustained occupation. Life in a periodically wet floodplain would be enhanced by constructing a raised platform, walkway or foundation for dwellings^38^.

Woodworking was also a precursor for the Mid-Pleistocene invention of hafting, which increased efficiency of basic actions such as chopping and scraping^39^. The interlocking logs from BLB5 anticipate hafting’s core concept: the combination of two or more parts to make a construction^40^, enhancing our understanding of the technical cognition of these toolmakers^11^. Exceptional conditions of preservation give us this glimpse of a capacity to create a built environment by hominins hitherto perceived as mobile foragers with limited technological diversity^41^.

## Methods

### Sedimentology and depositional environments

Sediment details were recorded from BLB1, BLB3 and BLB5 (Fig. 1b) which focused on the oldest sediments exposed at the base of the river cliff. BLB3 and BLB5 sampled the lowest 1–2 m of the cliff exposure; BLB1 spanned the full exposure from the base to the cliff top. A single sediment log of the BLB1 succession is shown (Fig. 1c) with only the lowest section reported in detail.

Sediments are dominated by well-sorted sands exhibiting weakly developed cross-bedding up to 50 cm thick. Sands vary between fine/medium to coarse grained. Gravels are rare, occurring as small discrete clasts in sandy units, or as lags associated with erosional horizons. Sediments are consistent with lateral and downstream accretion of point and mid-channel bars within a sand-dominated fluvial channel system. Fine-grained sediments are less abundant but occur as either thin (less than 10 cm) discrete lenses or organic-rich clays and silts, frequently associated with fossil tree trunks or more laterally continuous units of silts and clays, their geometry indicative of accumulation in abandoned channels. The occurrence and arrangement of these differing units indicate a sandbed river that underwent regular low-flow conditions and episodically shifted, or avulsed, across its floodplain. Nothing in these sequences indicates major change in river process or substantial environmental transitions. Particle sizes were analysed using a Malvern Master Sizer 3000 (Supplementary Information Section 1).

### Luminescence dating

Sixteen samples for dating were collected at Site BLB by hammering opaque plastic tubes into the sediment. A combination of field gamma spectrometry, laboratory alpha and beta counting and geochemical analyses were used to determine radionuclide content, and the dose rate and age calculator^42^ was used to calculate radiation dose rate. Sand-sized grains (approximately 150 to 250 µm in diameter) of quartz and potassium-rich feldspar were isolated under red-light conditions for luminescence measurements and measured on Risø TL/OSL instruments^43^ using single-aliquot regenerative dose protocols^44,45^. Single-grain quartz OSL measurements dated sediments younger than around 60 kyr, but beyond this age the OSL signal was saturated. pIR IRSL measurements of aliquots consisting of around 50 grains of potassium-rich feldspars were able to provide ages for all samples collected. The pIR IRSL signal yielded an average value for anomalous fading of 1.46 ± 0.50% per decade. Where quartz OSL and feldspar pIR IRSL were applied to the same samples, the ages were consistent within uncertainties without needing to correct for anomalous fading. The conservative approach taken here has been to use ages without any correction for fading. If a fading correction had been applied then the ages for the wooden artefacts would be older (Supplementary Information Section 2 and Supplementary Tables 2.8 and 2.9).

Using the pIR IRSL ages without any fading correction, values at each of the five sections (BLB1 to BLB5; Extended Data Table 1) are in stratigraphic order (considering the calculated 1*σ* uncertainties on the ages), except for two samples. At BLB2, the central ages for samples KF10 (355 ± 18 kyr) and KF11 (422 ± 28 kyr) are reversed. The reason for this is unknown. The age of this section is estimated by taking the mean of the two ages, giving a value of 383 ± 28 kyr. The ages fall into four groups, containing between three and six samples in each group. The oldest cluster of ages contains three samples found at or below current river level in sections BLB3 (KF14: 492 ± 32 kyr; KF15: 453 ± 27 kyr) and BLB5 (KF01: 487 ± 28 kyr). The second cluster is stratigraphically above the first and contains three samples, one in section BLB5 (KF02: 416 ± 37 kyr) and the two ages at BLB2 that are in reverse order (KF10: 355 ± 18 kyr and KF11: 422 ± 28 kyr). The third cluster is stratigraphically above the second and contains four samples in BLB1 (KF09: 355 ± 33 kyr), BLB3 (KF13: 326 ± 16 kyr) and BLB4 (KF16: 322 ± 19 kyr; KF17: 316 ± 18 kyr). The youngest group contains six samples found in BLB1 (KF04: 11 ± 1 kyr; KF05: 15 ± 2 kyr; KF06: 29 ± 2 kyr; KF07: 38 ± 3 kyr; KF08: 40 ± 2 kyr) and BLB3 (KF12: 62 ± 4 kyr). The ages of the three oldest clusters were calculated by combining ages using the central age model^46^, using only the random errors for weighting (Supplementary Information Section 2 and Supplementary Tables 2.8 and 2.9), and adding in quadrature the systematic errors to determine the total uncertainty on the weighted mean ages. The ages for the clusters are 476 ± 23 kyr, 390 ± 25 kyr and 324 ± 15 kyr. If a correction for anomalous fading of 1.46 ± 0.50% per decade is applied, the ages for these three clusters and the associated artefacts are older (554 ± 32 kyr, 452 ± 29 kyr and 377 ± 21 kyr).

### Wood identification and anatomy

Subsamples of the specimens excavated from BLB2, BLB3, BLB4 and BLB5 were examined in the biological preparation and scanning electron microscope (SEM) laboratories in the Department of Scientific Research, British Museum. Given the three-dimensional (3D) nature of wood anatomy, each wood sample, irrespective of its size, was fractured manually to show transverse, radial longitudinal and tangential longitudinal sections^27^. Each uncoated transverse, radial longitudinal and tangential longitudinal wood section was mounted onto SEM aluminium stubs.

SEM examination of the wood samples and comparative reference specimens (prepared and mounted using the same method) was undertaken in a variable pressure SEM, Hitachi S-3700N using the backscatter electron detector at 15 kV, with the SEM chamber partially evacuated (40 Pa). Magnifications ranged from ×50 to ×1,000. Preferred working distance was approximately 14 mm but was raised or lowered from 12 mm to 16 mm as required. With the backscatter electron detector, 3D mode (rather than compositional) was selected for maximum topographical information and to reveal diagnostic features for identification to genus or species level (Supplementary Information Sections 3 and 4).

### Wood surface analysis

The BLB wood was airfreighted from Zambia to the University of Liverpool for analysis (2019–2022). Specialist advice was followed and the samples kept submerged in cold tap water in the dark with trace chlorine providing protection from fungal contamination. The water was changed regularly and the samples monitored for fungal growth; none was found. Definition of surface shaping marks on pieces from BLB3 (object 660) and BLB5 (object 1033) degraded during storage, but no changes were seen on wood from BLB2 (object 219) and BLB4. The wood from the latter two contexts was recovered from clay and silt deposits with a high organic content, whereas the older contexts of BLB3 and BLB5 were primarily sands. The lower piece from BLB4 was found cracked in situ. Object 1033, BLB5, had two cracks (Fig. 2a) on either side of the central notch. The lower trunk was left in situ; excavation pits were backfilled.

Samples are being conserved at York Archaeological Trust, UK, before being returned to Zambia for curation (Livingstone Museum).

Radiocarbon dating and species identification samples were collected using a Chroma Gesellschaft F3400 Cork borer (5 mm internal diameter). Sample depth ranged from 8 mm to 12 mm. Samples for infrared and Raman spectroscopy (to identify mineralization and burning) were collected from BLB5 (object 1033) and BLB2 (object 219) using a Swann-Morton no. 3 scalpel with 10a blades. Samples were 5 mm × 5 mm × 2 mm or less and freeze dried before analysis to remove moisture. Spectra were recorded using samples larger than attenuated total reflectance diamond crystals. Opus 7.2 software was used to process sample spectra using 30 scans covering a wavelength range of 500–4,000 cm^−1^. An infrared spectrum of modern *C. zeyheri* was provided by Royal Botanic Garden Kew as a comparator, with spectra recorded on three separate regions. Silica peaks occurred in the archaeological samples but were absent in the modern samples, indicating mineralization of the BLB wood. A sample collected from the notch in BLB5 (object 1033) showed a flattened silica peak suggestive of carbonization from burning^47^. No evidence of burning was found using portable X-ray fluorescence (pXRF) and SEM energy-dispersive X-ray spectroscopy analyses of samples from BLB5 (object 1033), BLB3 (object 660) and BLB2 (object 219). Raman spectroscopy was unsuccessful; samples were obliterated by the laser before analyses were completed (Supplementary Information Section 5).

All wood was photographically modelled while submerged to minimize damage from drying (University of Liverpool). RTI was used to produce two-dimensional models/images of complete specimens. RTI models differ from a standard photographic image in being viewable from different angles, allowing all lighting angles to be observed as well as exposing low-relief surface features. RTI photographs were taken using a Sony Alpha 77ii DSLR using manual settings—F stop value F16, with a shutter speed of 1/200— ISO-200. During photography, the wood was submerged and placed close to the surface of the water to minimize distortion. RTI models were built using RTIbuilder v.2.0.2, HSH highlight-based fitter v.1.0.1, and viewed with RTI viewer v.1.1.0. (www.Culturalheritageimaging.org). Measurements from RTI models were taken with imageJ (v.1.53t) and TPsDig2 v.2.31. GIMP v.2.10 was used for postprocessing RTI images (removal of background and insertion of digital scales). Underwater photogrammetry was performed using a custom-built watertight container housing a camera. Two cameras were used depending on the size of object being modelled: Sony A77M2 with a Sigma 30 mm lens for larger objects and Sony A7M3 with a Sony 90 mm macro lens for smaller objects. Photographs were taken in raw format and converted to JPEG using Adobe Lightroom. Three-dimensional photogrammetry models were constructed using Agisoft Metashape (v.1.8.1, build 13915, 64 bit) selecting the highest accuracy settings. All 3D models were refitted and rotated for imaging as.stl files using MorphoDig (v.1.6.7 64 bit). Additional photographs were taken after objects were allowed to dry for up to 15 min to increase visibility of surface features.

### Water modification and saturation experiments

A pilot study was undertaken to assess the effects of flowing water and water saturation on preservation of modified wood surfaces. The preservation of shaping marks on wood at Site BLB contrasts with the reported absence of surface marks on wood recovered 60 m downstream at Site B, in similar late Acheulean deposits^17^. Site B is on the actively eroding outer bend of the Kalambo River, whereas BLB lies on a straight stretch of the main channel (Fig. 1b). Controlled experimentation was undertaken involving modification of standardized wood samples using stone tools and a steel knife, with the surfaces recorded before and after a period of exposure to abrasive flows. Thirty-two specimens of soft wood (*Abies alba*, silver fir) of standardized size were hand-stripped of their outer bark, eight specimens were left unmodified as a control sample, four were allowed to dry before placing in the flume tank and four were kept wet. The specimens analysed were branches taken from trees of similar ages and growth. A soft wood was chosen for its low-density fibre structure which increases the likelihood of modification by an abrasive flow regime and swelling from saturation. The 24 worked specimens were split into three groups of eight: one group was worked with a steel knife, one group with a bifacially worked flint tool and one using a unifacially retouched flake tool. A steel knife was incorporated to increase the contrast in edge morphologies and marks produced. All specimens were cut to similar sizes, with lengths ranging from 85.6 mm to 118 mm, and were scraped unidirectionally for 10–15 min until surfaces were flat to enhance the identification of minor modifications caused by abrasive flow and those made by intentional shaping. Analysis was primarily visual, including inspection with the naked eye, SEM analysis and RTI. The combination of analytical techniques provided functional and morphological information. Blind tests were undertaken to assess the analyst’s ability to identify working methods and tools using RTI and SEM.

A GUNT HM166 flume tank was used which gives a flow velocity of up to 1.5 m s^−1^. The speed was set at approximately 1 m s^−1^ (0 to 2.0 Knots) with water depth of 80 mm to cover the sticks and a channel width of 50 mm, resulting in a proportional flow rate equivalent to an average river flow^48^. The experimental channel was filled with coarse- and fine-grained sand, like those from the basal levels of Site BLB. The wood specimens were secured in a wooden rig and periodically rotated to ensure equal exposure of surface to the path of the flowing sediments. The flow velocity allowed for the greatest possible effect on the sticks, which were left in the water for 2 weeks, 70 hours of which involved exposure to the flow, and the remainder in still water overnight and weekends. In addition, four of each group of eight sticks were kept in water after exposure to the flume tank and before analysis to assess the effect that water absorption had on surface modification visibility. Once the 2-week period was completed, half the sticks were allowed to dry and were re-analysed using the methods above. The results from each set of analyses were compared, to assess the differences before and after exposure to flowing water and the effects of saturation on the preservation of surface morphology.

The experiments showed that (1) abrasive flows smoothed facet edges and ridges, but tool marks remained visible; (2) saturated specimens lost definition of facet junctions from swelling, and minor surface irregularities from shaping were smoothed or disappeared altogether; and (3) fluvial exposure did not create marks mimicking intentional shaping marks. Differences between the four dry control samples and the four saturated samples were only visible under SEM examination (Supplementary Information Section 6).

### Woodworking experiments

To characterize the patterns of wear left on wood from working and shaping with stone tools at Kalambo Falls, four transverse scraping experiments were performed at the University of Liverpool and a chopping experiment undertaken at the University of Liège, TraceoLab. Quartzite was used because of its prevalence as a raw material of choice for making small and large tools^17^. The quartzite was sourced in the UK and from Kalambo Falls. Modern reference samples of wood found at Kalambo could not be sourced. Two European woods of similar dry density were selected for their comparability to the two species that feature in the Kalambo sample, *C. zeyheri* (0.58 g cm^−^^3^)^49^ (BLB4, BLB5) and *K. Africana* (0.58–0.64 g cm^−^^3^) (BLB2)^50^. The European species were ash (*Fraxinus excelsior*) (0.56–0.64 g cm^−^^3^) and elm (*Ulmus procera* and *Ulmus minor*) (0.55−0.60 g cm^−^^3^) (https://www.engineeringtoolbox.com/wood-density-d_40.html). Elm has long, intertwined fibres that give strength, making it a less tractable hardwood for working with stone. We do not have comparable fibre length data for the Zambia woods. Retouched and un-retouched flakes were used unidirectionally, handheld, following the grain for 20 min each to scrape bark-covered green ash and green elm. A quartzite biface was hafted (juxtaposed)^51^ to a wood handle made by a traditional craftsman from Chiungu Village, Kalambo Falls and used in a scraping motion for 20 min.

A single wedge experiment was undertaken using a tapered cut section of hazel to laterally split open a larger section of birch, resulting in perpendicular compression damage on the active face of the wedge in contact with the birch. The wedge tip, which was initially not perpendicular, snapped during use leaving a perpendicular break. The struck end of the wedge displays a series of small radial cracks. The asymmetric and undulating morphology of the tapered end of BLB3 (object 660) (Fig. 2b and Extended Data Fig. 6) was replicated in experimental burning and scraping of hazel wedge tips using flint flakes. No other evidence exists for burning on object 660. The deep crack on the base of object 660 (Extended Data Fig. 6) was replicated by a splitting experiment using a flint handaxe placed point down on the butt of a hazel pole and struck using a wood baton. A crack propagated from the point impact and spread one-third down the pole. The cracking of object 660 may have resulted from intentional splitting to create firewood, or from use as a portable work surface (‘stake anvil’), with a stone working edge inserted into the base enabling bimanual processing of organic materials (stake set into firm ground and gripped between knees for support).

The chop marks visible at end a of BLB4 upper object (compressed log) (Extended Data Fig. 8) were replicated by chopping fresh elm using a quartzite cleaver hafted in a socketed wooden handle (elm). The tool proved effective and succeeded in producing the deep cuts seen on a, and the distinctive steps on a were produced automatically during chopping. Depth of the cuts varies between 5 mm and 8 mm, and their width corresponds to the width of cleaver used (77 mm). The cleaver remained sharp and effective for about 15–20 min of work, after which the edge became blunt and tore fibres more than it was cutting them. A blunter tool edge also results in a more fibrous appearance of the chopped wood. The depth of cuts reproduced experimentally is slightly shallower than the 13–25 mm visible on the BLB4 log. However, the tool used was lighter than archaeological examples found at the site (experimental cleaver: 402 g, with handle 1,020 g; which is well below the 1,787 g of an archaeological example from BLB5), its spine plane angle more obtuse (30–35° compared with the 29–31° of the archaeological cleaver) and its edge already showed some removals before use. Cuts of 13–15 mm in depth, as on the BLB4 log, are easily attainable with a heavier cleaver with a more acute edge angle (Supplementary Information Section 7).

### Ethnographic observations in Zambia

A traditional woodworker, John Mukopa, Mungwi area (Kasama), Northern Province, participated in ethnographic research undertaken 26–28 July 2022 by Moto Moto Museum, and University of Liverpool staff were supported by the Endangered Material Knowledge Programme (https://www.emkp.org) (EMKP2020SG01). Ethics approval was obtained from the University of Zambia (HSSREC-20220-APR-022), the University of Liverpool (REF: 11408) and the British Museum/EMKP. Observations were made on the trees selected, tools used in felling and shaping, and marks left on wood surfaces from shaping. Inferences were drawn for interpreting the production sequence in making object 1033 (BLB5) and possible uses of the log with chopped ends (BLB4) (Supplementary Information Section 8).

### Reporting summary

Further information on research design is available in the Nature Portfolio Reporting Summary linked to this article.

## Online content

Any methods, additional references, Nature Portfolio reporting summaries, source data, extended data, supplementary information, acknowledgements, peer review information; details of author contributions and competing interests; and statements of data and code availability are available at 10.1038/s41586-023-06557-9.

### Supplementary information

Supplementary Information Supplementary Sections 1–8, figures, tables and references.

Reporting Summary

## Figures and Tables

**Extended Data Table 1:**
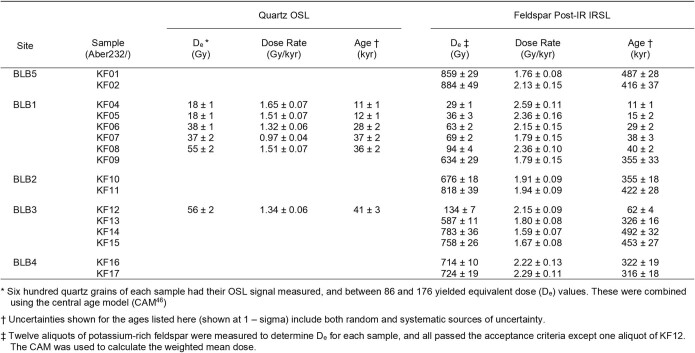
Luminescence ages at Site BLB, Kalambo Falls. Six samples could be dated using the quartz optically stimulated luminescence signal (OSL). All 16 samples were dated using the post-infrared infrared stimulated luminescence (post-IR IRSL) signal from potassium-rich feldspar grains

## Data Availability

Photographic data (wood objects, RTI and photogrammetry models) supporting this research are openly available via Liverpool Research Data Catalogue (10.17638/datacat.liverpool.ac.uk/2200).
