# Supplementary material for: Evidence for the earliest structural use of wood at least 476,000 years ago
Source: Nature. 2023 Sep 20;622(7981):107–11. doi: 10.1038/s41586-023-06557-9 (PMC10550827; doi:10.1038/s41586-023-06557-9)
Supplement: Supplementary file 1 — Supplementary Sections 1–8, figures, tables and references. [file 41586_2023_6557_MOESM1_ESM.pdf]

nature portfolio

https://doi.org/10.1038/s41586-023-06557-9

Supplementary information

Evidence for the earliest structural use of wood at least 476,000 years ago

In the format provided by the authors and unedited

Directory of Supplementary Information:

1. Sedimentology and depositional environments of Kalambo Falls, Zambia

2. Luminescence dating at Kalambo Falls, Zambia

3. Identification of wood samples from Site BLB Kalambo Falls, Zambia

4. Wood anatomy of modified objects from Site BLB Kalambo Falls, Zambia

5. Infrared (IR) spectroscopy investigation of waterlogged woods from Site BLB, Kalambo Falls, Zambia

6: Taphonomic experiments on wood surface preservation

7: Wood working experiments

8: Ethnographic observations of woodworking, Zambia

1. Sedimentology, and depositional environments of Kalambo Falls, Zambia

The following sections describe the sedimentology of BLB 1, 3 and 5 (Figure 1.1) and then use these sequences to present a depositional model for the site. Part 1b concludes by discussing the implications of this model for the archaeology of the site. Part 1c shows the contexts (plans and sections) of the wood object recovered from BLB5, BLB3, BLB2 and BLB4, (see Figure 1b in the main article for the location of all excavation blocks).

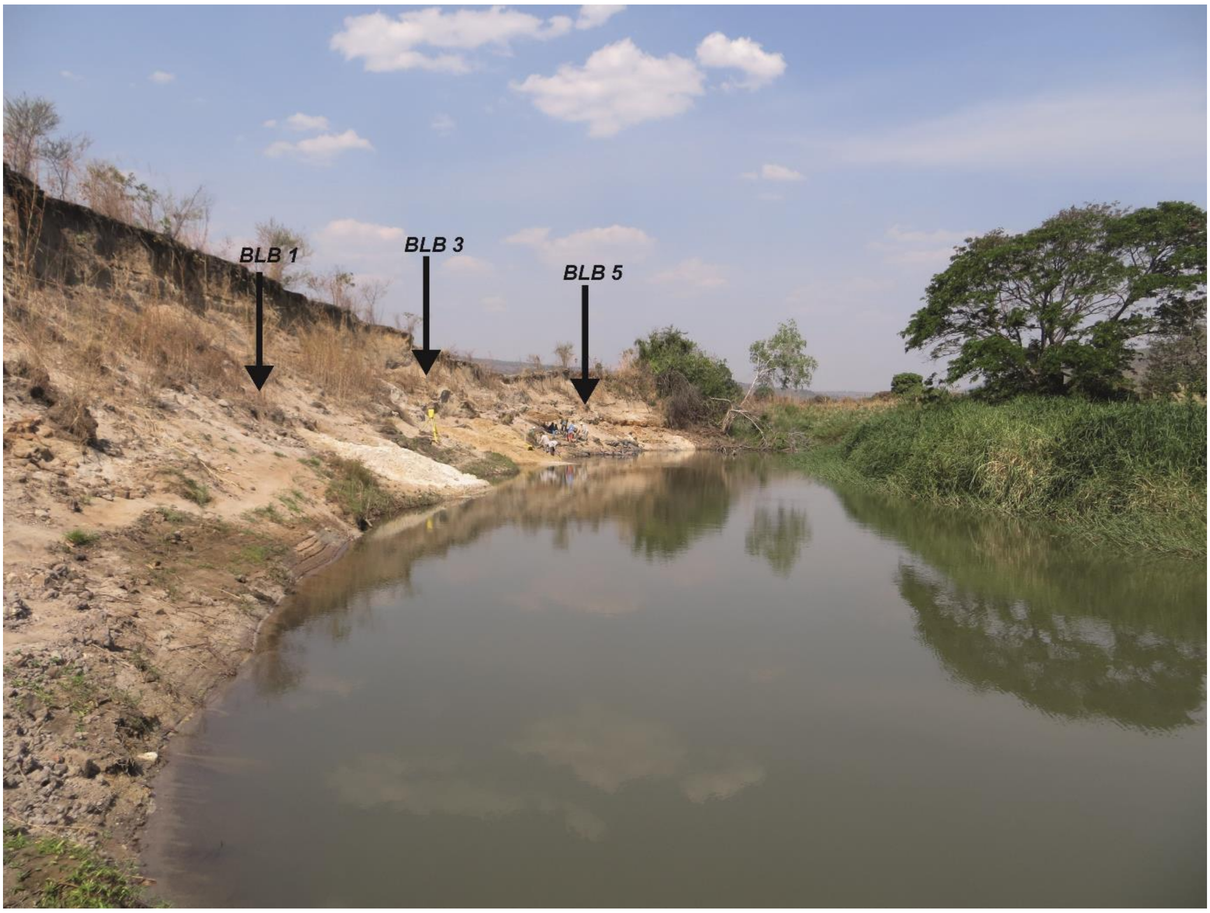

Figure 1.1 – Overview upstream of site BLB showing the location of the three main localities in relation to the Kalambo River and the river-cut cliff face.

BLB 1

The full succession of BLB 1, a geological section, is shown in Figure 1.2 but only the lowermost section (BLB 1 section j), equating to the bottom 1.4 metres, is discussed in detail (Figures 1.3, 1.4). BLB 1 section j is dominated by sands with the lowermost unit being a very well-sorted fine-sand light grey in colour (A). The top of this unit is truncated by a horizontal erosional contact over which a sequence of coarse-sand with occasional cross-bedding and pebble lags and crudely-developed horizontal stratification (B) occurs. The pebble lags in this unit, shown in Figure 1.2 are discontinuous as can be seen in Figure 1.3, at depths of 80 and 100 cm (pebble and granular horizons). It is important to note, therefore,

that these gravel horizons are not stratigraphic markers and vary in height and thickness depending upon which wall of the excavation is being studied/analysed. The lowermost 40 cm of unit B contains rip-up clasts of clay (homogeneous in structure and pale brown in colour) that represent the erosion of pre-existing clay beds and the incorporation of the derived fragments into the resulting sand. The uppermost part of this unit is crosscut by cross-bedded coarse-grained sands which reach thicknesses of up to 50 cm in height (C). These sand units are truncated by a horizontal erosional contact overlain by a pebble dominated gravel lag (D). Within this lag are fragments of a white/light grey consolidated material that has previously been described as “calcrete” (Figure 1.4), but laboratory analysis shows very limited reaction with 10% HCl. It is likely that these “calcrete” fragments are in fact highly consolidated fragments of pre-existing silt-rich fluvial sediments that have been “ripped up” and incorporated into the fluvial sediments. The grey/white appearance of these fragments, which makes them appear carbonate rich, is probably a function of the fact that they have been severely dried out.

The sediment characteristics of BLB 1 section j indicates that it was dominated by deposition by flowing water currents. The range of particle sizes indicates that flow speed varied during the accumulation of the sequence but that the degree of variation was low as most units are characterised by sand-sized sediments. The sedimentary structures present are typical of the movement of sand bar bedforms with the horizontal stratification with pebble lags (B) being typical of the downstream pro-gradation of mid-channel bars whilst the cross-bedded sediments are more typical of the lateral migration of point bars (C). The sediments are, therefore, typical of a sand dominated river system that is undergoing lateral migration. The occurrence of clay rip-up clasts indicates that the channel migrated across an exposed clay bed but the concentration of these intra-clasts within the lowermost part of the sequence implies that this exposure of fine-grained materials was rapidly buried.

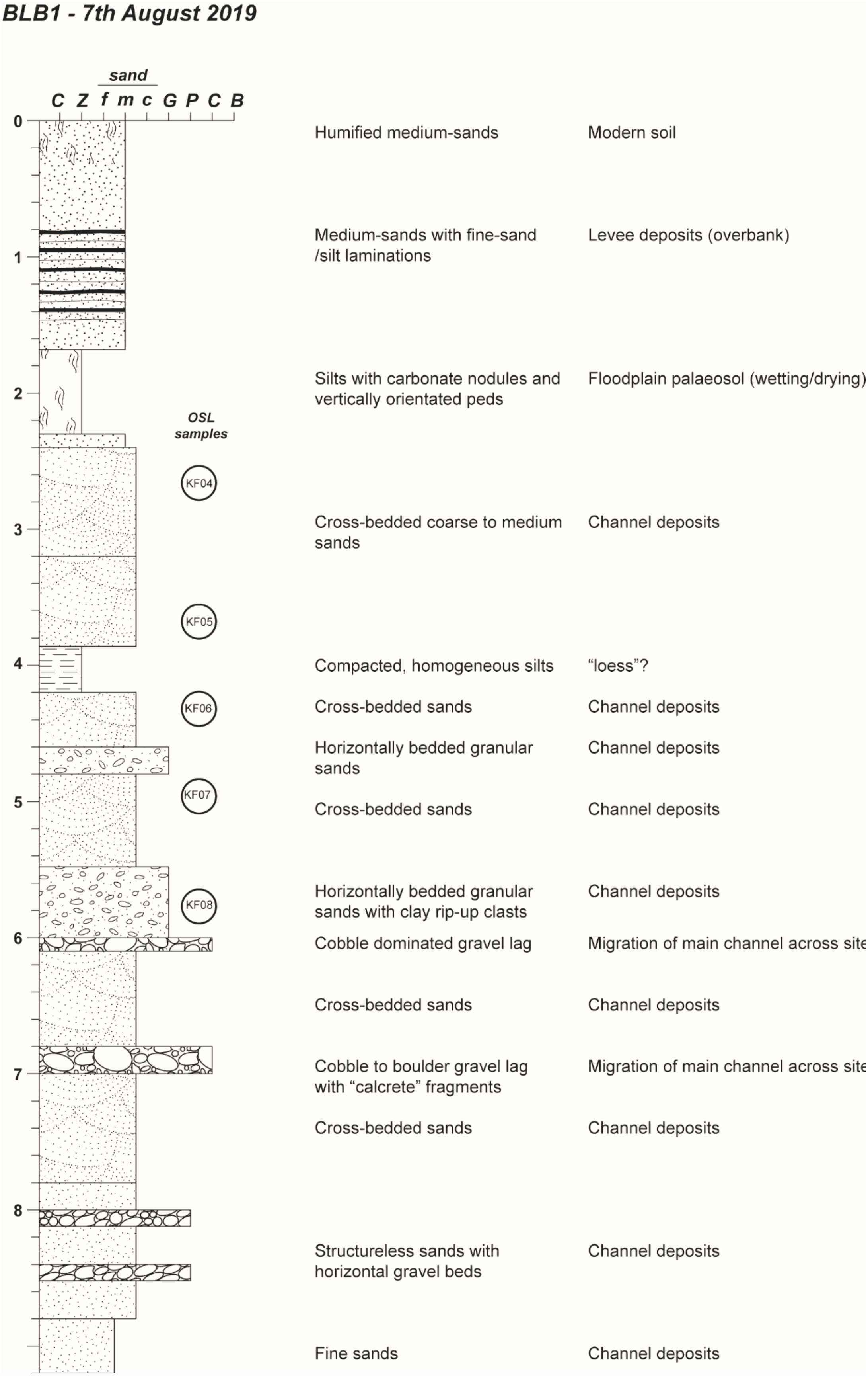

Figure 1.2 – Sedimentary log summarising the Pleistocene succession in BLB 1.

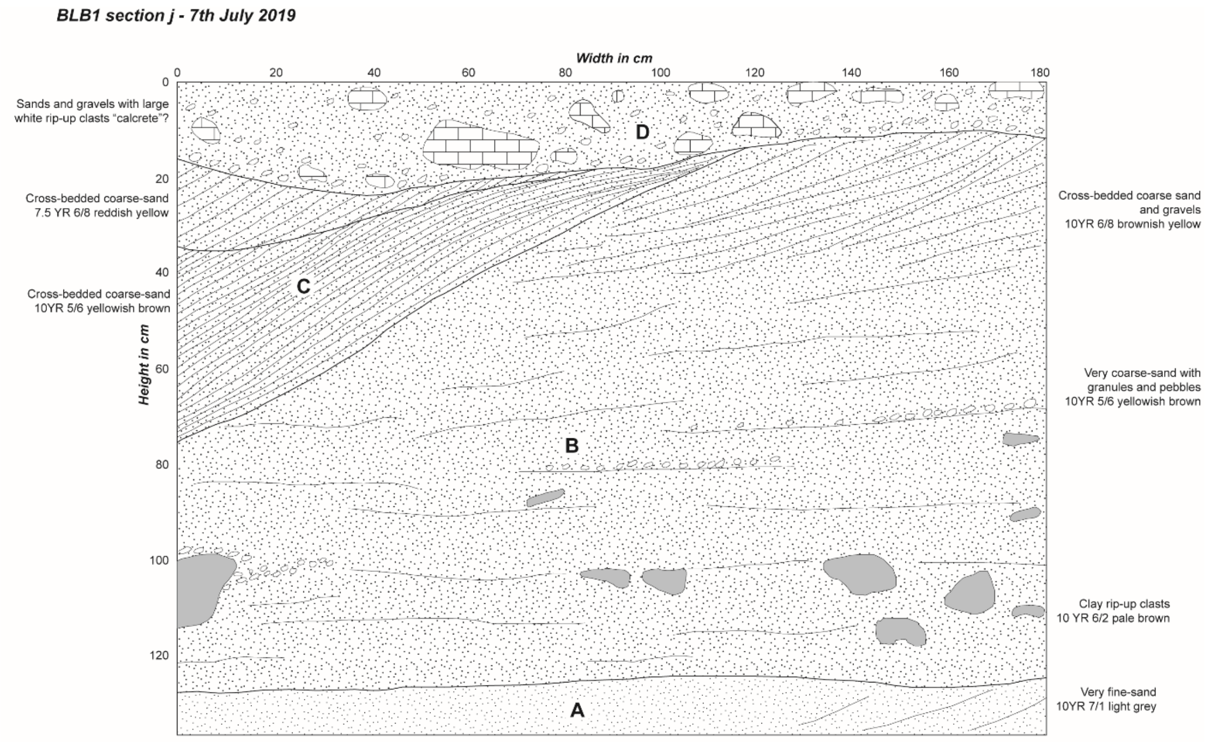

Figure 1.3 – Sediment section of the lowest section (j) in the BLB 1 sequence.

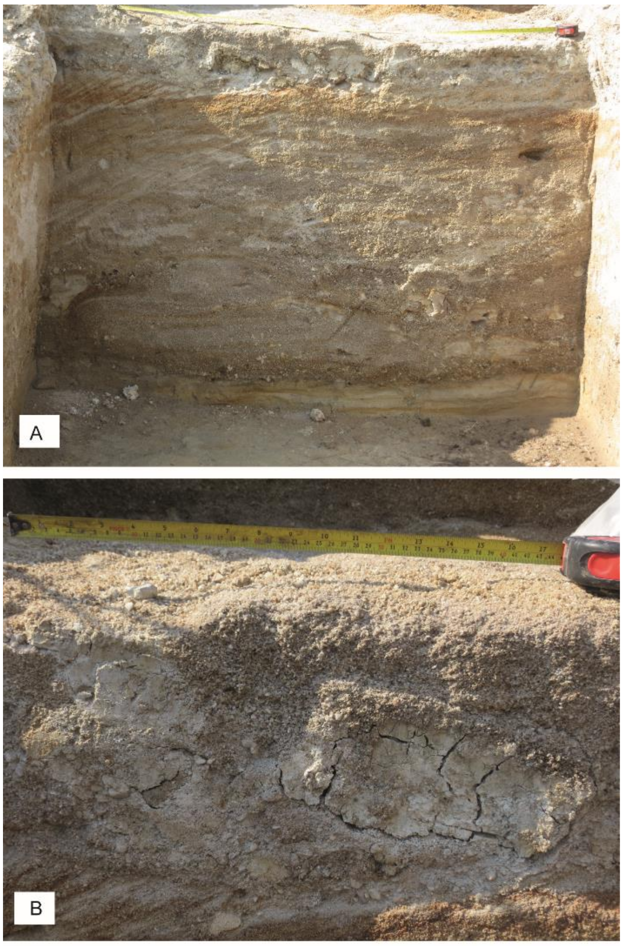

Figure 1.4 – Sediment sequence of BLB 1 section j (A). Detailed photographs of "calcrete" fragments in the uppermost part of BLB 1 (B).

BLB 3

The sediments exposed in BLB 3 can be divided into 5 main phases of deposition (Figure 1.5 and 6). The first is deposition of a coarse sand which contains granular material and occasional pebbles (not represented in Figure 1.5). This is overlain by fine/medium sand with well-developed horizontal and occasional crossbedding (B). At the contact between these two occurs a well-preserved fossil tree trunk, associated with which are smaller fragments of woods and discrete dark grey lenses of silt and clay, rich in matted organic material (A). The fine-medium sands are overlain by a dipping, laterally discontinuous bed of clays that has undergone deformation and erosion (C). This fine-grained bed is overlain by cross-bedded units of sands and gravels with well-developed stratified units dominated by coarse-sands and pebbles (D).

The BLB 3 sequence again contains evidence for deposition by flowing water currents with the sand and the sand/gravel dominated units being typical of the migration of fluvial bars/bedforms within an active channel. The cross-bedded, stratified sands and gravels that cap the sequence are typical of the lateral aggradation of point bars in association with the active migration of a sand dominated river channel (D). The sequence also shows, however, two episodes of slack, or stagnant, water deposition (units A and C). The first of these is seen in the lenses of clays/silt material that reflects the ponding of fine-grained sediments and associated organics around a fossil tree trunk from which objects 660 and 661 were recovered (A). This fine-grained deposit occurs at the boundary between a coarse-grained sand unit and a fine/medium-grained sand unit (Fig. 1.5). The relatively thin and discontinuous nature of these lenses implies that this period of low energy/still water deposition was relatively short-lived, possibly occurring during a single dry season/spell between flood events. During the fall in river water levels large floating objects, such as tree trunks, would become snagged on the surface of sand bars with the effect of ponding water either side of the obstruction. It is within this ponded water zone that fine-grained sediments and organics would accumulate, including object 660 which may have been transported by the river or left by hominins on this floodplain surface (Fig. 1.6).

The second episode of slack water deposition is represented by the clay unit (C) which is continuous across much of section *C-D* but absent from section *A-B*. The clay unit is up to 15 cm in thickness and represents a more extended period of still water conditions than the lower organic lenses. This is suggested because clay sedimentation requires almost completely still water conditions to accumulate and is, therefore, typically a relatively slow process. The deposition of this unit, therefore, most likely represents sedimentation in association with a body of standing water on the floodplain during an extended period when channel activity at this location was minimal. This most likely reflects a period where the channel has migrated or avulsed to a different part of the floodplain. The deposits which overly the clay unit reflect a return to higher energy deposition with the sediments recording the transport and deposition of coarse-grained sands and gravels. The stratified units with relatively steeply dipping bedding are suggested to reflect the lateral migration of point bars in an active channel system. The abundance of clay rip-up clasts within the lower part of this unit suggests that the

active channel has eroded, and locally deformed, the underlying clay unit with fragments of this fine-grained unit being remobilised into the overlying sediments.

BLB 3 was intensively sampled for particle size analysis with four samples coming from Units A and B (below the fine-grained sediments of Unit C) and 3 samples coming from Unit D (overlying Unit C). A single sample was taken from Unit C itself (Figure 1.5). Except for the sample from Unit C all sediments were well to moderately sorted with sand being the dominant size fraction but with varying amounts of gravel being present. This is consistent with deposition by moderate energy current flow processes that experienced variable energy.

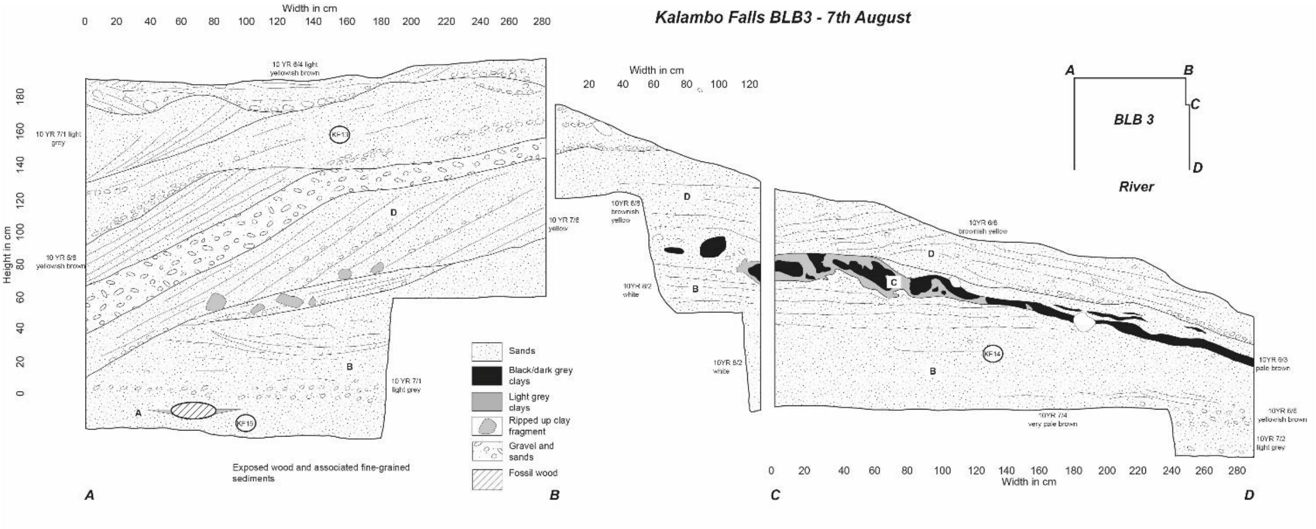

Figure 1.5 – Sediment exposures in BLB 3 and location of luminescence samples (KF15, KF14 and KF13).

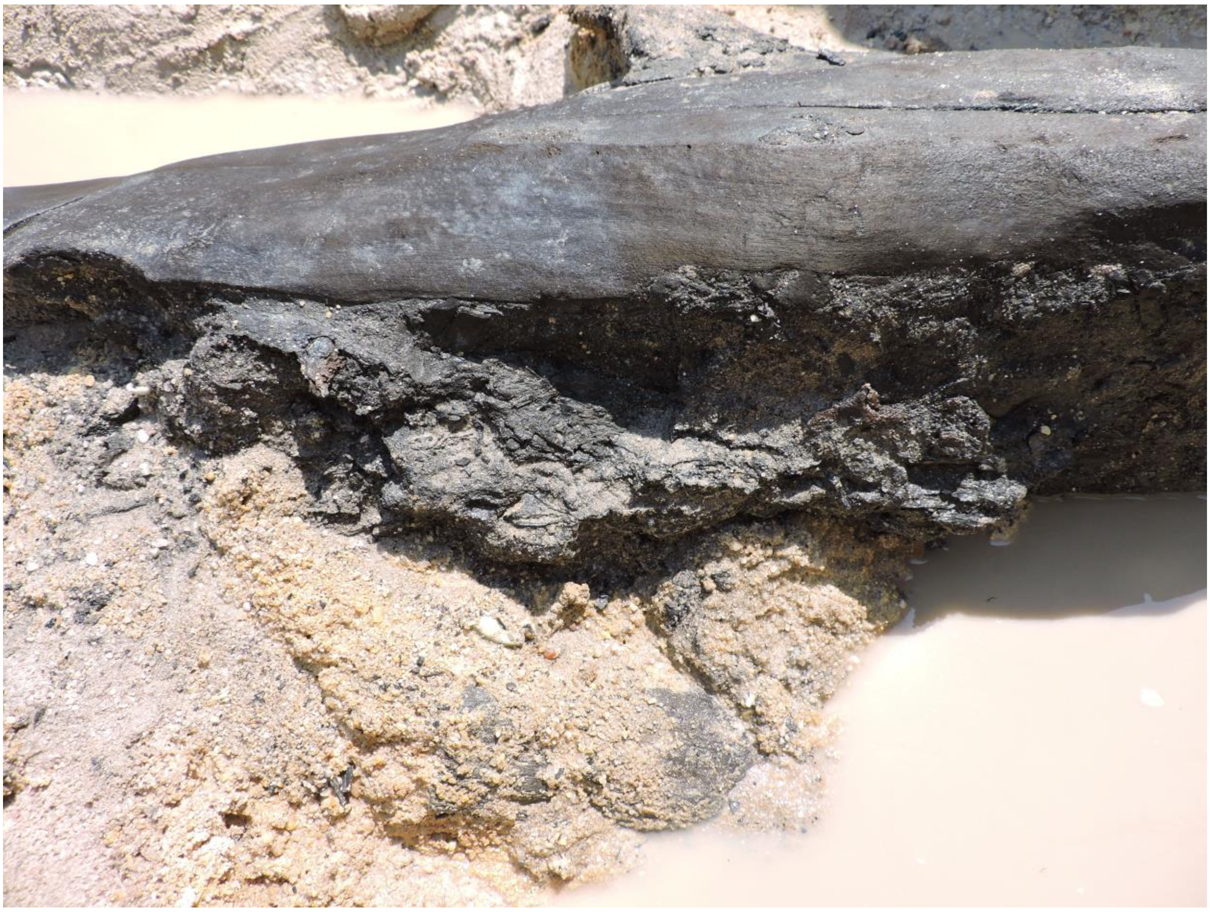

Figure 1.6 – Photograph of BLB 3 showing the location of the tree trunk and the relationship of the fine-grained, organic rich sediment lenses beneath and to its side. This context in which the ‘wedge’ (object 660) and a V-shaped branch of Ficus sp., (object 661) were found.

The coarsest sediments, and, therefore, the highest energy flow conditions, occur at the base (where sands = 53% and gravels = 42%) but for much of the sequence sands vary between 67-90% of the sediment. Unit C is the finest-grained unit present consisting of 55% fine sand and ca 30% silt/clay (Figure 1.7). This is consistent with this unit being deposited under still water conditions, however, it is likely that the persistent action of low energy flows still occurred during the accumulation of this unit, as witnessed by the presence of a significant sand fraction.

BLB 5

The sediment sequence in BLB 5 North (Figures 1.8 - 1.10) has a stratigraphy that is broadly similar to that of BLB 3, consisting of a lower unit of thin and discontinuous organic-rich silt/clay lenses associated with large fossil tree trunks (A), and an upper, thicker bed of fine-grained sediments (C). These two fine-grained units are separated by relatively coarse-sands with occasional small pebbles and granular material (B). The lower unit of thin fine-grained lenses are underlain by, and the tree trunk is set into, coarse-grained sands (see 1c, Figure 1.16). As such the suggested mode of accumulation for BLB 5 is like that of BLB 3, characterised by phases of active channel activity, represented by the sand units, interspersed by stagnant/still water deposition, represented by the lower discontinuous fine-grained lenses

and the upper thicker and more continuous fine-grained beds. As with BLB 3 the fact that the lower fine-grained lenses are thin and discontinuous indicates that they probably reflect relatively short-lived periods of still water conditions, e.g. deposited during low flow intervals between wet seasons. Furthermore, the occurrence of the lenses in association with large tree trunks is, again, consistent with the idea that these fine-grained sediments accumulate within sluggish water bodies ponded up and downstream of a tree trunk snagged on a sand bar or emplaced by the occupants of the site at a time of still water in the case of BLB5. The uppermost fine-grained unit (C) is different from that seen in BLB 3, in that it comprises massive silts and clays within which discrete sand laminations and lenses occur.

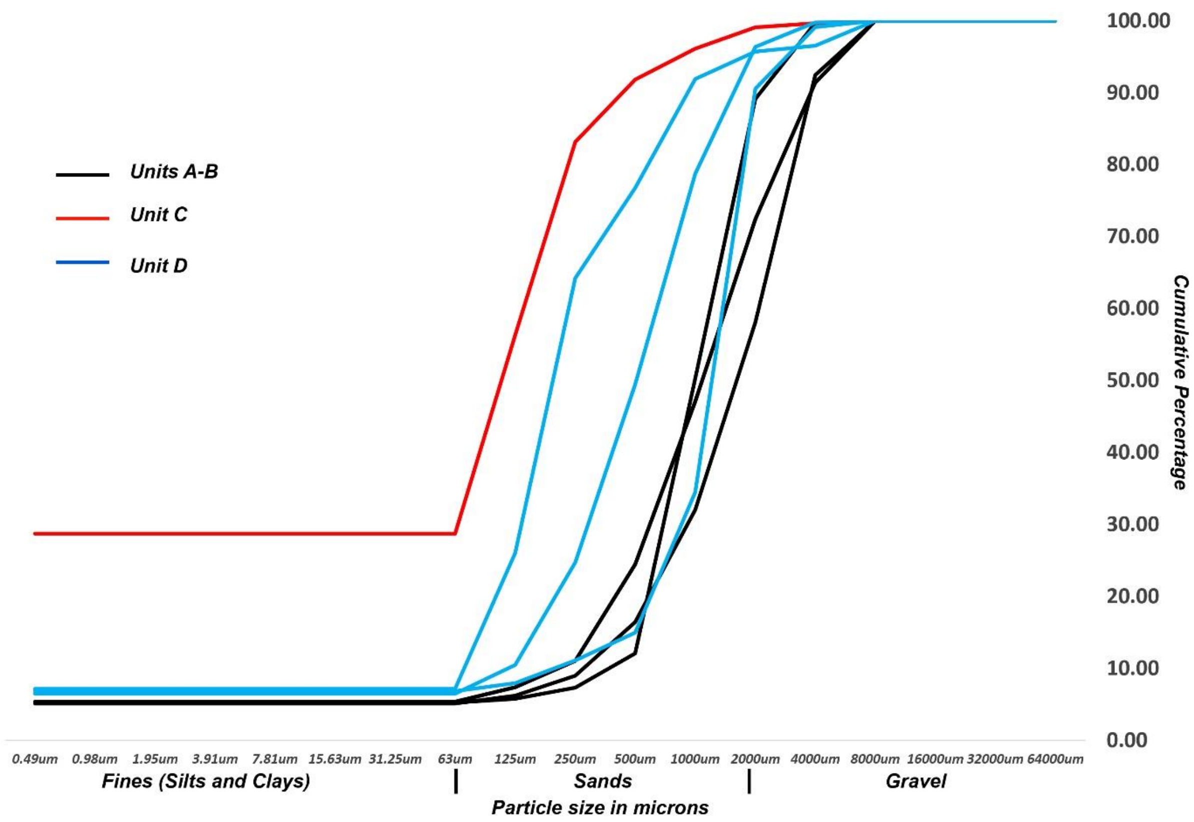

Figure 1.7 – Particle size data from BLB 3. The particle size distribution of each sample is estimated from the diffraction properties of the sediment in suspension.

This unit is interpreted as reflecting sedimentation within an abandoned channel on an active floodplain where the primary mechanism of deposition is fine-grained sediments falling out of suspension from still water. Episodic large floods from the still active channel system, located elsewhere on the floodplain cause the system to break its banks washing small pulses of coarser sand into the abandoned channel.

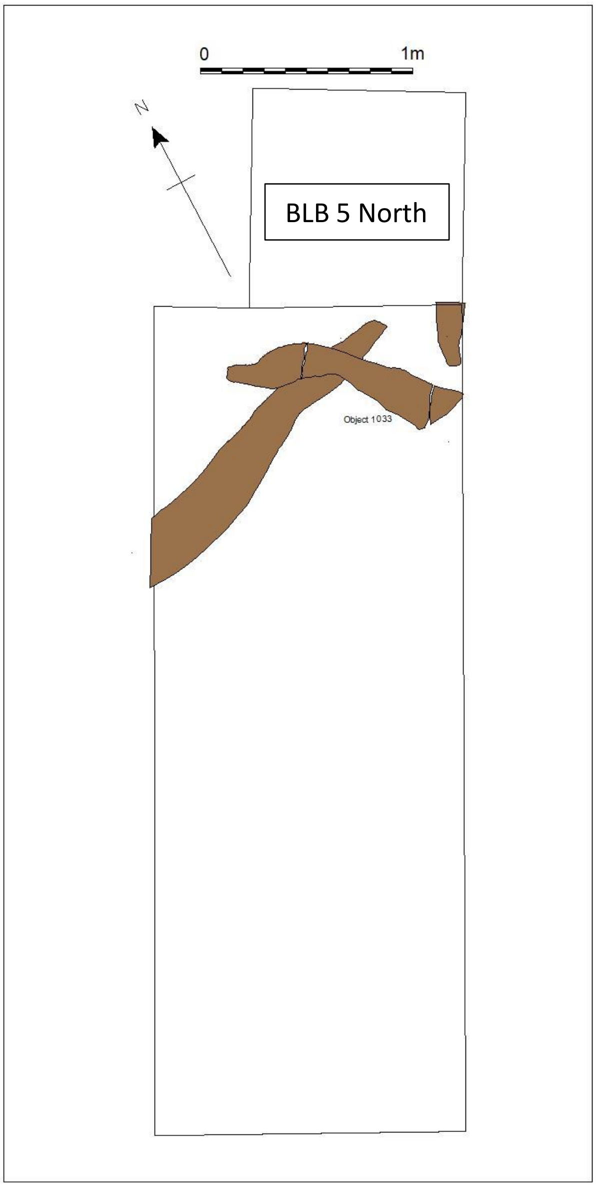

Figure 1.8 Plan view of BLB 5 showing the overlapping tree trunks (object 1033 on top) and the location of the northern extension (BLB 5 North) beyond the overlapping trunks.

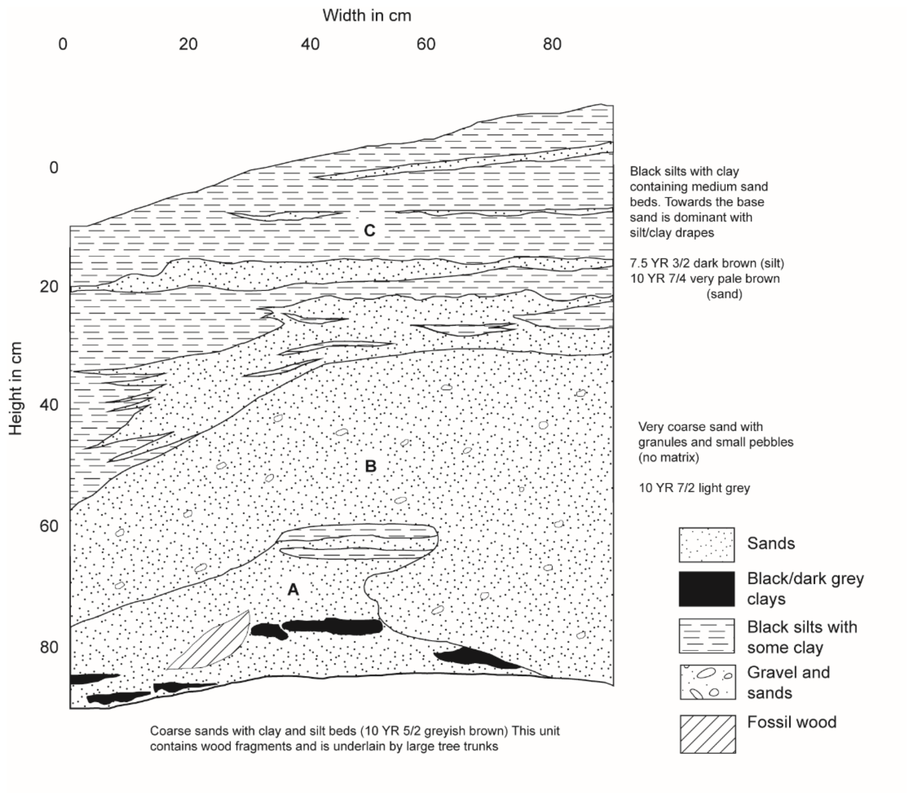

Figure 1.9 – Sediment section of BLB 5 North.

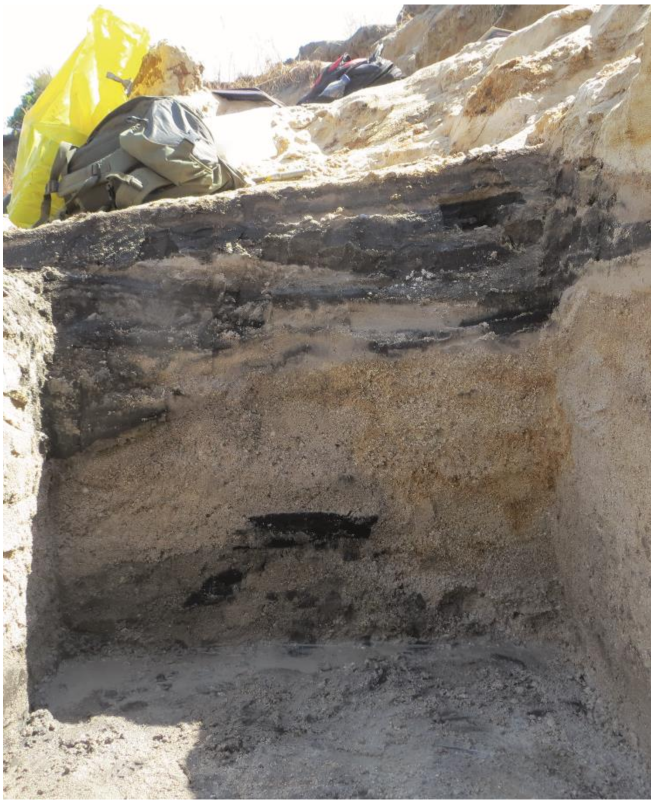

Figure 1.10 – Photograph of the sediment section of BLB 5 North.

Depositional model of the Kalambo Falls sequence

The studied sequence at Kalambo Falls is consistent with the action of a sand-dominated river channel undergoing lateral migration on its floodplain (Figure 1.11A). The sand and sand/gravel units, which make up the majority of the sequences, represent the aggradation of sand bars, both mid-channel and point bars, under high-flow/flood conditions (Figure 1.10B - 1, -3 and -5). Phases of lower energy river activity are represented by fine-grained sedimentation. The sediment sequence in BLB3 and BLB5 indicate that an earlier short-lived phase of channel stability (Figure 1.11B -2) and a later more prolonged period of stable conditions (Figure 1.11B-4) occurred during the accumulation of this sequence. Neither of these phases of fine-grained sedimentation are recorded in BLB 1. This absence presumably reflects the fact that: 1) low-flow/still water sedimentation occurred in localised topographic lows that are not conducive to producing extensive, laterally continuous units across a wide area, and 2) the nature of the high-energy channel flow means that it is likely that many low energy deposits will be eroded and removed from the sediment record. The latter suggestion is supported by the abundance, within BLB 1, of clay rip-up clasts testifying to the removal and remobilisation of pre-existing fine-grained sediments.

Significance of the Kalambo Falls sediment sequence for early human occupation

Four main observations can be made from the Kalambo Falls sediment sequence which are pertinent to understanding the human occupation of the site. First, that all of the sediment variations within the sequence can be explained by internal variability with a constant fluvial system (i.e. migration of a single channel back and forth across a floodplain). None of the variability requires the occurrence of significant environmental or climatic change. Consequently, there is no reason to assume that human occupation at the site occurred during a period of environmental or climatic instability. Second, that evidence for human occupation (stone tools, worked wood) occur in association with periods of landscape stability. Evidence for human occupation is found in association with fine-grained units when channel flow was diminished or when channel process had abandoned this site. Third, that the stratigraphy of the site implies that there are at least two distinct phases of occupation recorded here. An earlier one, with worked wood and stone tools associated with the lower discontinuous organic lenses and tree trunks, and a later one, associated with the fine-grained sediments infilling the abandoned channel. Whilst there is no way of showing whether humans abandoned the site between the deposition of these two units, the stratigraphic succession shows that the upper and lower fine-grained units are separated by a phase of renewed channel activity and, therefore, by a time interval. Finally, in the above interpretation the occurrence of large tree trunks within the sequence is ascribed primarily to natural processes, as in the case of the BLB 3 trunk. In rivers affected by seasonal variation in flow height it is common for wood, floating on the river surface, to become beached or snagged on exposed bedform surfaces as the flow level gradually drops. Whilst such a process is common in such settings it does not discount the possibility that these features were either re-arranged or entirely emplaced by human agency as in the case of the BLB 5 construction.

1b – Preservation of the Kalambo Falls sediment sequence

Two aspects of the preservation of the Kalambo falls sequence are relatively unusual. First, the preservation of sediments of such antiquity at the level of the modern river. It is clear that the river is relatively active and, in the absence of uplift to raise ancient sediments above the level of the present channel, the lowermost parts of the sequence, which contain the fossil wood, have been preserved in this setting for several hundred thousand years despite the high potential for erosion. Secondly, the preservation of the wood itself. In tropical settings the hot and humid climate frequently means that organic material undergoes rapid degradation resulting in a relatively low preservation potential. The quality of preservation of wood that is many hundreds of thousands of years in age within the Kalambo sequence is, therefore, both rare and surprising.

We ascribe these unusual preservation conditions to the unique setting afforded by the waterfall itself (Clark 2001). Waterfalls act as local base levels to which the catchment upstream of this point grades, meaning that the river cannot incise below the level of this feature. In earlier excavations an attempt was made to excavate the Pleistocene sediments below the river level to assess their depth to bedrock, but only 2 m below river level could be managed and the depth of the deposits remains unknown (Clark 1969:118 – 119). It is likely that this reflects the presence of a localised zone of tectonic subsidence. In this setting the preservation of older sediments is explainable by; 1) the lowering of their position in the landscape by subsidence, and 2) the inability of the river to incise below the level of the modern channel because of the local base level provided by the waterfall. Waterfalls also produce major constrictions in the catchment meaning that all water that flows through the upper part of the system is routed through a narrow gorge. This means that the area directly upstream of the waterfall frequently has elevated water tables because of this restriction. Furthermore, because the waterfall acts as a focal point for all the water in the catchment it is likely that in Pleistocene times this area was a site of permanently high ground water levels even during more arid phases. The combination of the fact that ground water levels were permanently elevated in this area and the fact that fossil wood occurs at relatively low levels in the sequence probably explains the exceptional preservation of this material. Waterlogged conditions rapidly become anoxic restricting biological activity and organic matter decay. The waterfall, and its impact on the fluvial environments directly upstream is likely to have played a major role in attracting early humans to this location. As outlined in this section it is also likely that these features played an important role in the preservation of both the Pleistocene sediments and the rich assemblage of fossil wood that they contain.

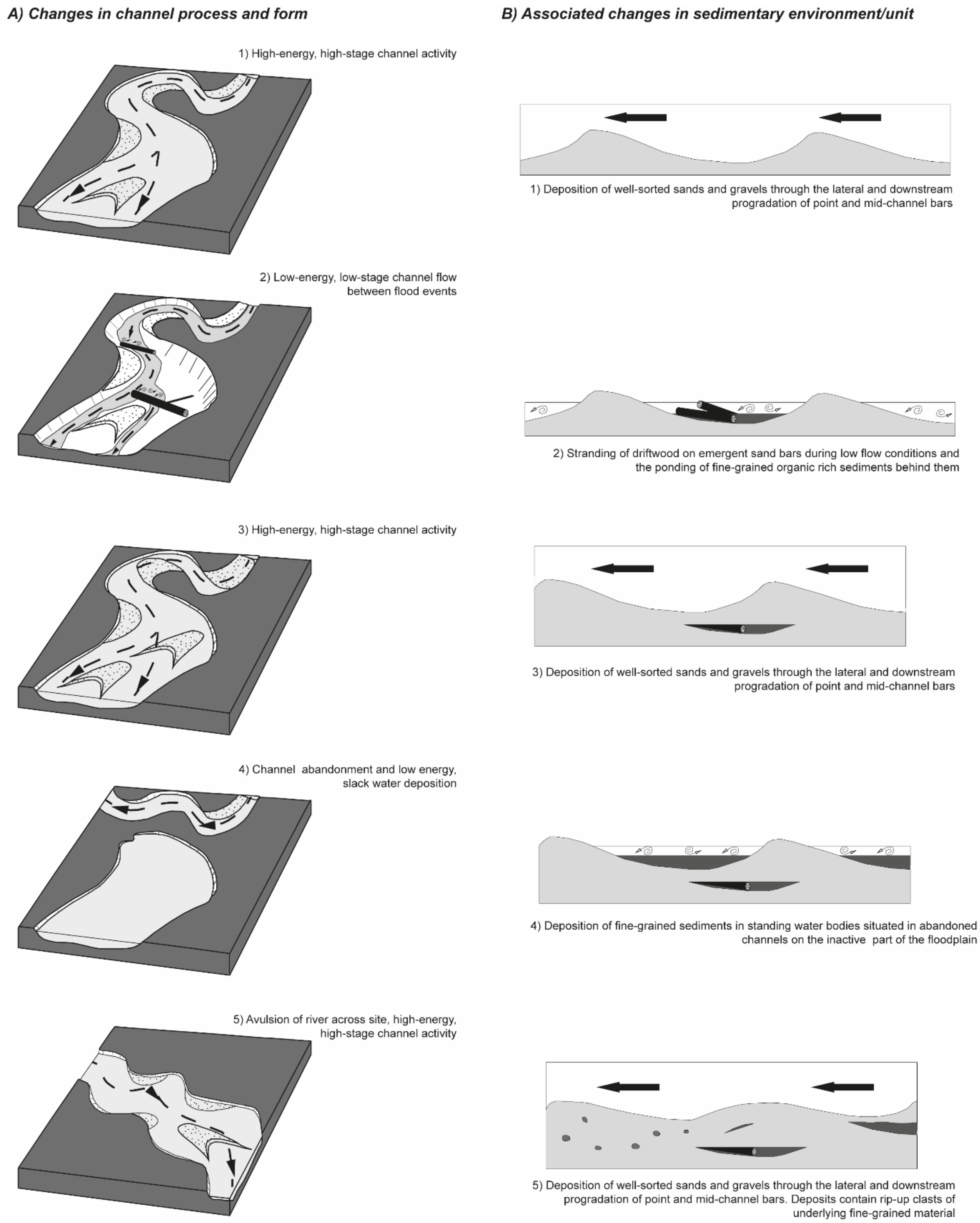

Figure 1.11 – A block diagram showing the changes in river process (A) that are implied by the observed changes in sedimentology (B) seen in the BLB sequences.

References

Clark, J.D. (Ed.) Kalambo Falls, Vol. I. Cambridge: Cambridge University Press. (1969)

Clark, J.D. (Ed.) Kalambo Falls, Vol. III. Cambridge: Cambridge University Press. (2001)

1c. Additional excavation plans and sections (see Figure 1b, main article for the location of all excavation blocks)

BLB 3

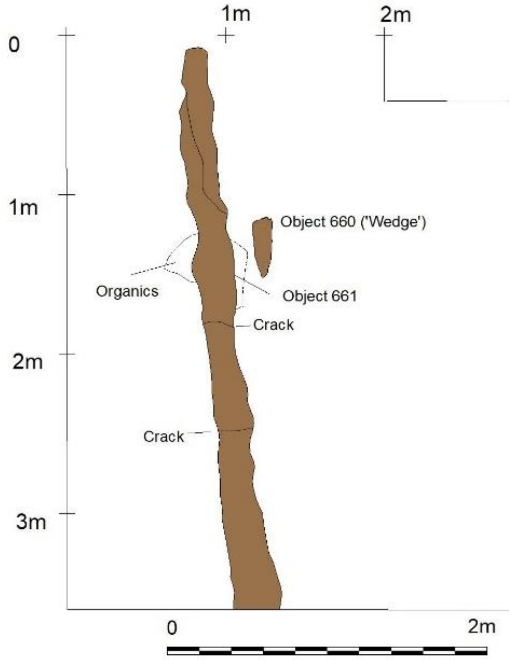

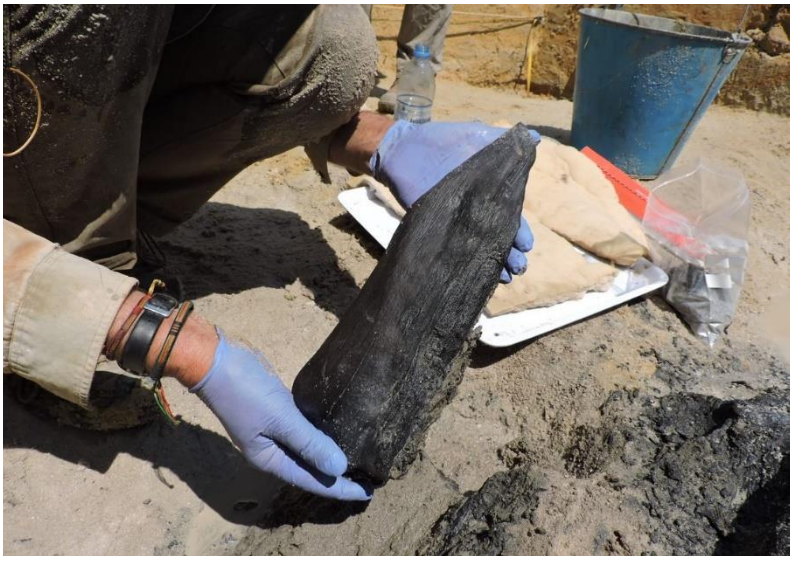

Figure 1.12. BLB 3 excavation plan (left) and recovery of object 660 (‘wedge’, right). Area of vegetal deposits shown in relation to the tree trunk. Object 661 lies partially beneath the tree trunk (see Figure 1.6).

BLB 2

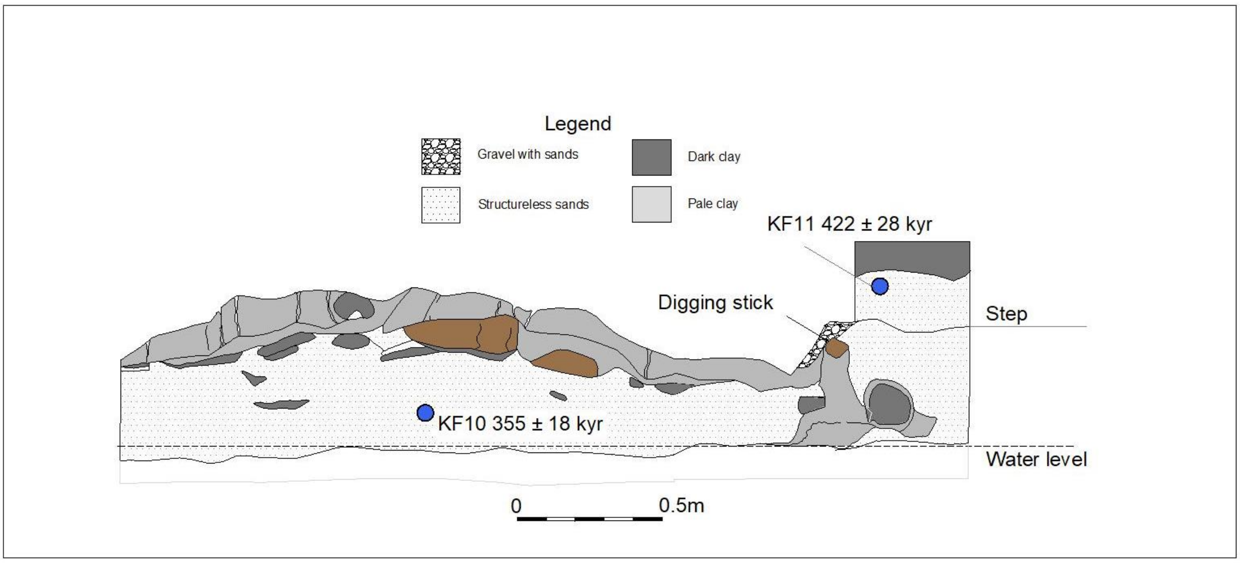

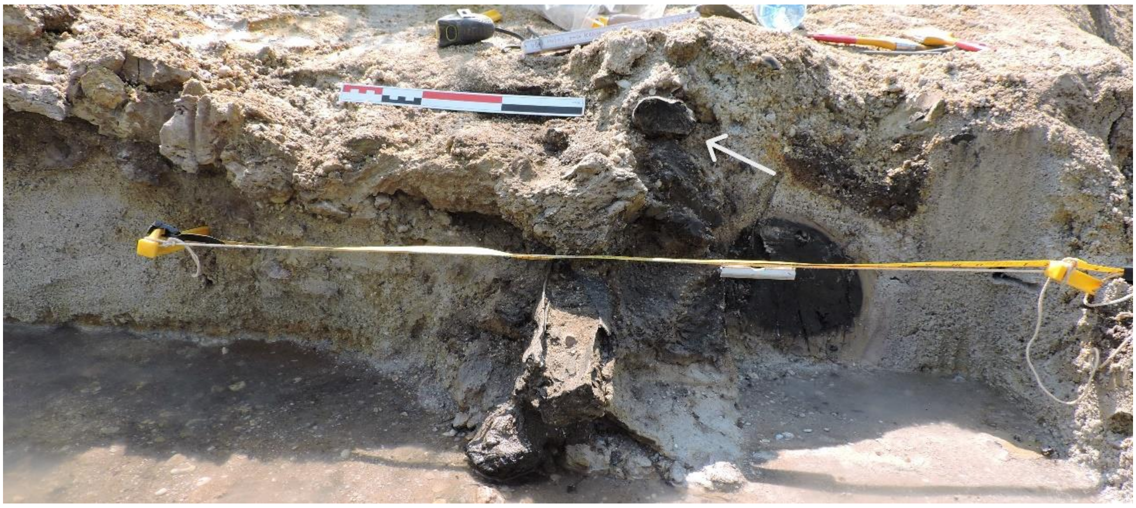

Figure 1.13 BLB 2 Section drawing (upper) and section on excavation (above). An extensive band of pale clay caps the deposit and incorporates object 219 ('digging stick') (white arrow on photograph), and two pieces of unmodified wood (in brown on section). The section drawing shows the location of dating samples (KF10, KF11).

BLB 4

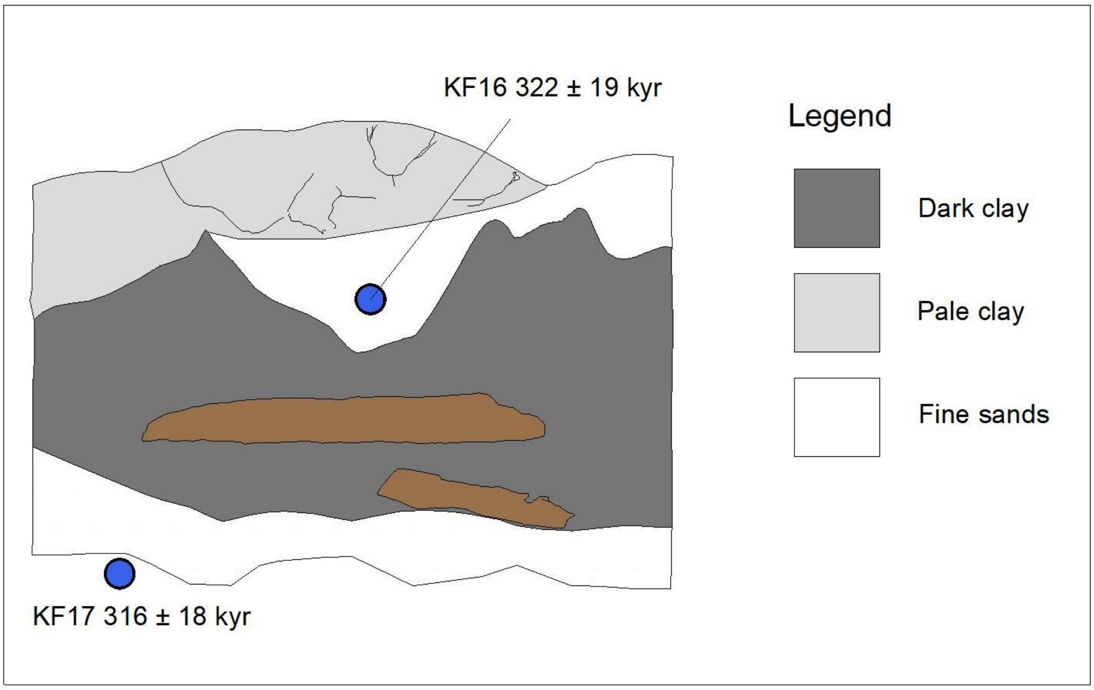

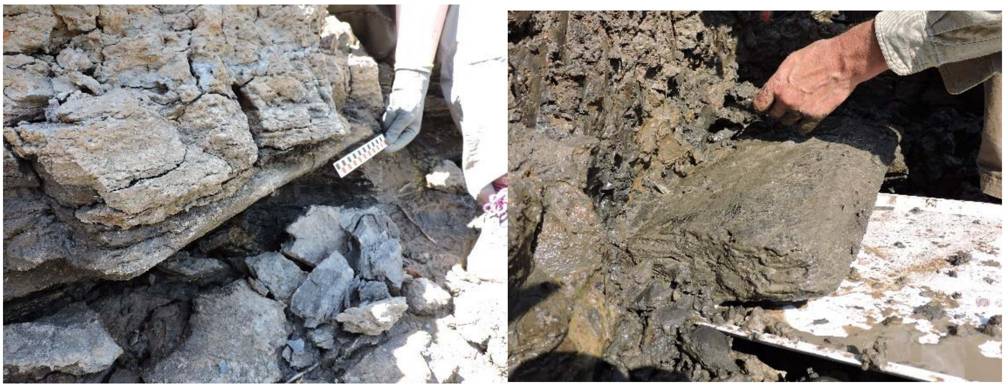

Figure 1.14 BLB 4 Section drawing (top) and deposit before and during excavation (bottom left and right). The section drawing shows the location of the dating samples in relation to the dark organic clays preserving the two objects (cut log and 'notched stick'). The photograph shows the compressed cut log as discovered eroding from the clays (left) and its excavation (right).

BLB 5

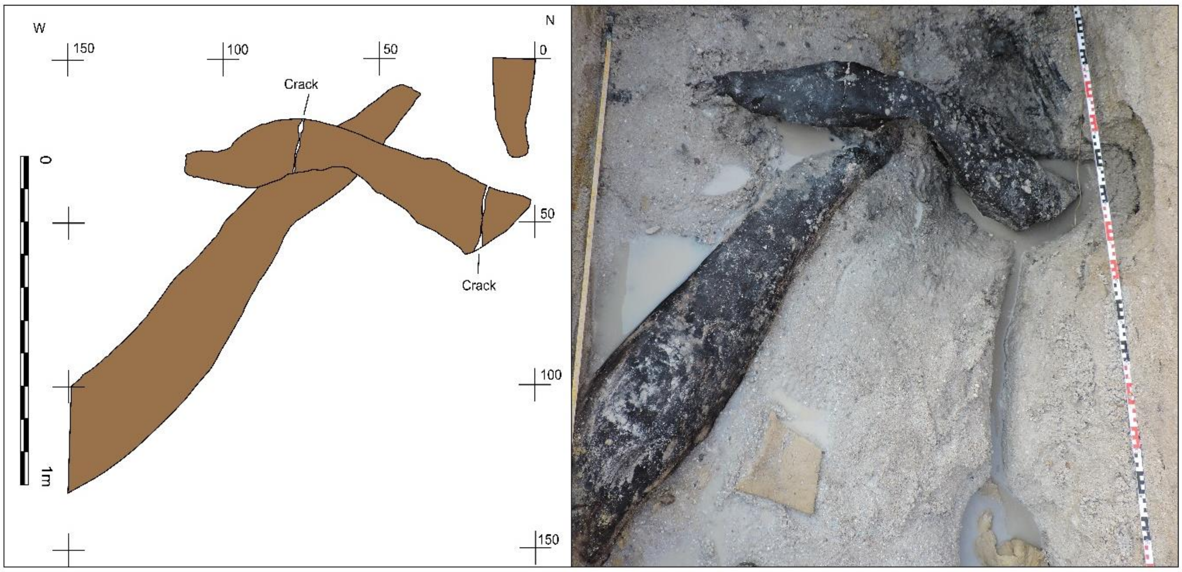

Figure 1.15 BLB 5; plan view and photograph of overlapping logs (object 1033) during excavation.

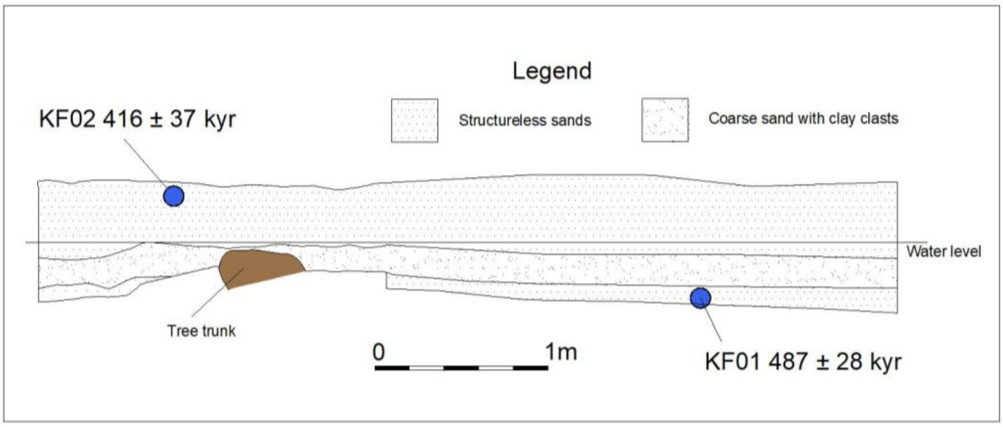

Figure 1.16. BLB5 west wall section showing the underlying tree trunk in relation to the water level, sediments and dating samples KF02 and KF01. The tree trunk lies in structureless sands and is overlain by coarse sands with clay clasts. The deposit below the tree trunk is unexcavated.

2. Luminescence Dating at Kalambo Falls, Zambia

To provide a chronology for the deposition of sediments exposed at Site BLB at Kalambo Falls, a suite of 16 samples from 5 sections (BLB1, BLB2, BLB3, BLB4 and BLB5) was collected for luminescence dating during excavations in 2019. Site BLB is only 100 m to the south of Site C North that was excavated in 2005 (Barham et al. 2015) and that was also dated using luminescence methods. Luminescence dating of the samples from Site C North used a combination of single grain quartz optically stimulated luminescence (OSL) measurements for the younger samples (less than ~50 ka), and thermally transferred OSL (TT-OSL) measurements for older samples (Duller et al. 2015). These results demonstrated that the oldest sediments at the site were ~300-500 ka. In this study of Site BLB, the younger samples are dated using single grain quartz OSL, but for the older samples, the post-infrared infrared stimulated luminescence (post-IR IRSL) signal from potassium rich feldspars was used. The post-IR IRSL approach has been used extensively in recent years (Li and Li 2011; Buylaert et al. 2012; Roberts 2012) and does not suffer from the same problems associated with TT-OSL (Duller and Wintle 2012).

1. Sample collection and processing

Samples for luminescence dating were collected by hammering opaque plastic tubes into the sediment. The ends of the tubes were sealed in the field to avoid light exposure and to retain moisture, and then returned to the laboratory for analysis. Sediment from the inner part of the tubes was isolated and further processed for luminescence dating analyses. Two of the samples, KF16 and KF17, which had been collected in close proximity to a thick organic-rich unit, were opened by cutting the sample tube in half lengthways so that the contents could be better observed before material from the inner part of the tube was isolated for processing. The plastic tube for sample KF16 was found to have passed through a sedimentary unit boundary during sampling as the front half of the tube consisted of the sandy material visible at the exposure during sampling, whilst the rear half of the tube consisted of black organic-rich material. The boundary between these units within the sample tube was clear and only the sandy material from the front half of the tube that was at least 1 cm away from the visible boundary was isolated for further processing. Sample KF17 was found to be sandy material throughout.

All sample processing and analysis was undertaken at Aberystwyth Luminescence Research Laboratory in red light conditions. Prior to chemical treatments, the coarsest samples were wet sieved to isolate grains ranging from 90-1000 µm diameter, whilst other samples were immediately subjected to chemical reagents. Carbonates were removed by treating the samples with 10% HCl for at least 3 days or until no continued reaction was observed. Initial reactions with HCl were mild or unobservable for all samples. After rinsing three times with distilled water, the sediment was put in to 20 vol H_2O_2 to dissolve organic matter before the samples were again rinsed three times and dried. Samples were then dry sieved using a stack of sieves ranging from 90 to 355 µm. Most of the collected sediment samples were dominated by grains >300 µm in diameter. However, sufficient masses of grains 90-250 µm in diameter were obtained to proceed with sodium polytungstate density separations. Specific grain size fractions

of all samples were separated to densities of 2.62-2.70 and 2.53-2.58 g/cm^3. Grains with a density of 2.62-2.70 g/cm^3 were then etched in 40% hydrofluoric (HF) acid for 45 min to dissolve any remaining feldspar grains and etch away the outer ~8-10 µm of the quartz grains which had been affected by alpha irradiation. After rinsing in concentrated HCl to ensure that no fluorides were precipitated, the grains were re-sieved to isolate a narrower grain size fraction and remove any remnant feldspar fragments. This sieved, HF treated sediment fraction of density 2.62-2.70 g/cm^3 is referred to as the quartz fraction of each sample. Grains with a density of 2.53-2.58 g/cm^3 were not treated with HF but were re-sieved to isolate a narrower grain size fraction and are hereafter referred to as potassium-rich feldspar fractions.

Eight samples from a previous study (Duller et al. 2015) on Kalambo Falls Site C North were also re-processed to obtain potassium-rich feldspar fractions. The ages reported in Duller et al. (2015) were measured using quartz OSL and thermally-transferred optically stimulated luminescence (TT-OSL) signals. The re-processing of these older samples started with sediment fractions of <2.62 g/cm^3 that had already been dry-sieved and treated with HCl and H_2O_2. The re-processing of these samples, for this study, therefore consisted only of sodium polytungstate density separations to obtain the 2.53-2.58 g/cm^3 potassium-rich feldspar fractions.

2. Dosimetry measurements

Four sets of measurements were made to determine the dose rate from the sediments collected at Kalambo Falls. At the time of collection, an Ametek DigiDart portable gamma spectrometer with a 2-inch diameter NaI detector was inserted into the sample holes left after collection of the luminescence samples. All samples were at least 30 cm below the current ground surface ensuring that a complete gamma field was present. A gamma spectrum was collected for an hour at each sampling site and an assessment of the total gamma dose was calculated using the threshold method (Mercier and Falguères 2007). The response of the portable gamma spectrometer detector was calibrated using artificially doped concrete blocks in Oxford, UK, (Rhodes and Schwenninger 2007) and checked for consistency using an internal standard housed in the Aberystwyth Luminescence Research Laboratory. Two sets of dosimetry measurements were undertaken within the laboratory. A dried subsample of the material collected from the end of each sample tube was ground in a TEMA mill to obtain a homogenised powder. This powdered dosimetry sample was then measured using two emission counting methods, thick source alpha counting (TSAC, using 582 and 583 alpha counters manufactured by Daybreak) and thick source beta counting (using a GM-25 beta counter, Bøtter-Jensen and Mejdahl 1988). Results from the portable gamma spectrometer and thick-source alpha and beta counting measurements are shown in Table 2.1 along with the radionuclide concentrations calculated from the measurements. The fourth set of measurements were to determine the chemical composition of the sediments and was also undertaken on the milled and homogenised powdered samples. Milled material was sent to SGS laboratories in Canada where 0.1g of sample was fused with a Na_2O_2 flux at 550°C before being dissolved in concentrated HNO_3 for measurement by ICP-MS. The concentration of U, Th and K were determined. As part of quality control, a sub-sample of the loess from Volkegem characterised

by De Corte et al. (2007) was also submitted at the same time and returned values consistent with those previously published.

To compare the dosimetry data from emission counting and from ICP-MS, the two data sets were used to independently calculate the infinite matrix beta dose rate (Fig 2.1). A straight line fit through this data has a slope of 1.01 and an R^2 value of 0.998 indicating the excellent agreement between the two sets of measurements. The agreement between the measurements also implies that there is no significant U-series disequilibrium in these sediments.

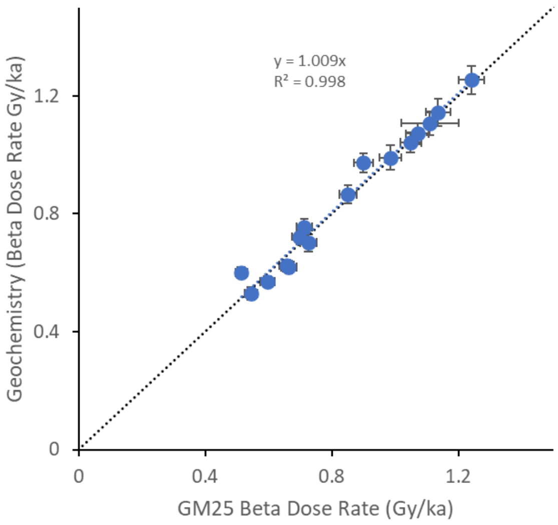

Figure 2.1**:** Bivariate plot comparing the infinite matrix beta dose rate measured using the GM-25 beta counter with that derived from concentrations of K, U and Th measured using ICP-MS at SGS laboratories. The dotted line is the line of equivalence. Beta dose rates were measured on three replicate sub-samples, and the error bar is calculated by combining in quadrature the standard deviation of those measurements with a 3% uncertainty estimated from the replicate measurements of the standard. The geochemical measurements were made on a single sub-sample. Uncertainties on the concentration of U, Th and K from the geochemical measurements is estimated at 5% based on replicate measurements of standards, and these uncertainties have been propagated in DRAC (Duncan et al. 2015) to derive the beta dose rate. All error bars are at 1σ. Error bars are included for all samples but are often within the symbols.

Dose rates for the samples were calculated using the Dose Rate and Age Calculator DRAC (Durcan et al. 2015) version 1.2. Cosmic dose rates were calculated using the equations in Prescott and Hutton (1994) based on longitude (31.24°E), latitude (8.59°S), and altitude (1110 m). The Kalambo River is actively eroding into the sediment making up the riverbank. For calculating the cosmic dose rate the overburden thickness was based on sample depth below the current ground surface into which the river is eroding. An overburden density of 1.8 g/cm^2 was assumed. Our geomorphological model for the area in which sites BLB and Site C North are found is one of continuous lateral channel change leading to erosion and subsequent

deposition of sediments (Duller et al. 2015). The base level for these changes is controlled by the rock lip of the Kalambo Falls and is unlikely to have changed dramatically through the period studied here. It is possible that the burial depth may have varied through time as successive episodes of lateral movement of the river channel occurred, but the ages are rather insensitive to the thickness of the overburden since the cosmic dose rate only makes up about 5% of the total dose rate. The impact of possible changes in overburden thickness through time can be illustrated by looking at one of the oldest samples (KF15). If the burial depth were 7 m instead of the value of 9.1 m used in Table 2.2, the age would fall by 1.1% from 453 ± 27 ka to 448 ± 26 ka. Reducing the burial depth to only 5 m would decrease the age by 2.5% (442 ± 26 ka) compared with the value used here. Whenever possible, gamma dose rates were calculated from field gamma spectrometry measurements, beta dose rates were calculated from thick-source beta counting measurements, and alpha dose rates were calculated from thick-source alpha counting measurements (Table 2.2).

The water content of all samples collected for luminescence were measured in the laboratory (Table 2.1) and varied from 2% to 24%. These measured values are likely to be underestimates of the long-term average water content during burial because of de-watering as a result of erosion and exposure of the sediments by lateral channel movement, and potential loss of water during sampling. Field evidence along with preservation of the wooden artefacts at site BLB support the idea that sediments at the base of the sections would be saturated, and a value of 25 ± 5% has been used for these. Lower average values are likely higher in the succession, and values varying from 20 ± 5% to 10 ± 5% have been used, informed by their burial depth and measured water content.

The sensitivity of the luminescence ages to water content can be illustrated for sample KF13. A pIR-IRSL age of 326 ± 16 ka is calculated with a water content of 20 ± 5%. This increases by 2.6% to 335 ± 17 ka for a water content of 25 ± 5%, and decreases by 2.8% to 317 ± 16 ka for a water content of 15 ± 5%. The exceptional preservation of wood at the base of site BLB, suggests that the basal sediments are likely to have remained saturated with water throughout their burial. Changes in water content for sediments higher in the sedimentary sequence are possible but would only impact the younger ages. Furthermore, since the water table upstream of the Kalambo Falls is controlled by the elevation of the rock lip of the waterfall, it is unlikely that major changes in water table level have occurred.

Table 2.1: Results of field gamma spectrometry and emission counting in the laboratory (TSAC and GM-25 beta counting) for samples collected at Site BLB, Kalambo Falls in 2019.

Sample	H_2O Content		Field Gamma (Gy/ka)	Infinite Matrix Beta (Gy/ka)	Alpha Activity (cts/ks/cm^2)	Calculated Radionuclide Concentrations		
	Meas	Used				K (%)	U (ppm)	Th (ppm)
KF01	10%	25%	0.43 ± 0.03	0.66 ± 0.02	0.40 ± 0.01	0.37 ± 0.06	0.95 ± 0.25	8.16 ± 0.83
KF02	12%	25%	0.43 ± 0.03	1.05 ± 0.03	0.69 ± 0.01	0.47 ± 0.09	2.45 ± 0.38	11.40 ± 1.26
KF04	3%	10%	0.55 ± 0.04	1.24 ± 0.04	0.45 ± 0.01	0.98 ± 0.07	1.94 ± 0.23	6.22 ± 0.48
KF05	3%	10%	0.61 ± 0.04	0.98 ± 0.03	0.27 ± 0.01	0.92 ± 0.05	0.80 ± 0.15	4.90 ± 0.50
KF06	2%	10%	0.64 ± 0.05	0.73 ± 0.03	0.21 ± 0.00	0.66 ± 0.04	0.75 ± 0.11	3.30 ± 0.37
KF07	2%	10%	0.43 ± 0.03	0.54 ± 0.02	0.21 ± 0.00	0.42 ± 0.04	0.90 ± 0.11	2.89 ± 0.36
KF08	3%	15%	0.65 ± 0.05	1.07 ± 0.04	0.67 ± 0.01	0.55 ± 0.09	2.03 ± 0.38	12.13 ± 1.27
KF09	3%	20%	0.54 ± 0.04	0.51 ± 0.02	0.31 ± 0.01	0.25 ± 0.04	1.28 ± 0.17	4.57 ± 0.55
KF10	15%	25%	0.44 ± 0.03	0.85 ± 0.03	0.40 ± 0.01	0.57 ± 0.06	1.52 ± 0.21	6.25 ± 0.69
KF11	15%	20%	0.52 ± 0.04	0.70 ± 0.03	0.28 ± 0.01	0.51 ± 0.04	1.25 ± 0.14	3.84 ± 0.47
KF12	3%	15%	0.60 ± 0.04	0.90 ± 0.03	0.44 ± 0.01	0.57 ± 0.06	2.00 ± 0.22	5.62 ± 0.71
KF13	2%	20%	0.56 ± 0.04	0.60 ± 0.02	0.36 ± 0.01	0.34 ± 0.06	0.79 ± 0.22	7.54 ± 0.74
KF14	13%	25%	0.37 ± 0.03	0.66 ± 0.02	0.31 ± 0.01	0.46 ± 0.05	1.01 ± 0.18	5.44 ± 0.58
KF15	14%	25%	n.d.	0.71 ± 0.03	0.35 ± 0.01	0.46 ± 0.05	1.35 ± 0.19	5.38 ± 0.63
[a]KF16-s	2%	25%	n.d.	1.14 ± 0.04	0.33 ± 0.01	1.00 ± 0.06	1.45 ± 0.17	4.40 ± 0.56
[a]KF16-o	24%	65%	n.d.	2.51 ± 0.08	1.56 ± 0.03	1.21 ± 0.17	6.21 ± 0.67	23.14 ± 2.23
KF17	18%	25%	0.73 ± 0.04	1.11 ± 0.09	0.52 ± 0.01	0.78 ± 0.13	1.49 ± 0.31	9.85 ± 1.04

[a] KF16-s and KF16-o refer to the two distinct sedimentary units occupying either end of the KF16 sampling tube (see Section 1). Only the sandy unit (KF16-s) was used for dating, but the organic rich unit (KF16-o) influences the gamma dose rate of the sample.

Field gamma dose rates were attenuated for water content in a two-step process by first un-attenuating the in-situ measurement based on the water content measured at the time of sampling to obtain a ‘dry’ gamma dose rate and then secondly attenuating this dry dose rate for the assumed average burial water content for the sample (which in all cases was higher than the water content measured during sampling). For two samples, gamma dose rates were calculated by other means than using the field gamma spectrometry data. Sample KF15 was too wet at the time of collection to use the gamma spectrometer, so the gamma dose rate was the average value derived from using the radionuclide concentrations calculated from the thick source alpha and beta counting measurements, and from the ICP-MS data. The gamma dose rate estimated for KF15 from laboratory measurements is statistically indistinguishable from

the gamma dose rate measured in situ for KF14, which was collected from the same unit at BLB3 (Table 2.2).

Another sample, KF16, has a more complicated gamma dose rate geometry. This sample was collected in a thin beige sand unit with the bottom of the sample tube ~5 cm above a clear contact with a black organic-rich horizon. However, the contact between the two units was not horizontal and instead rose diagonally into the sediment deposit increasing ~5 cm vertically after about 8 cm horizontally, such that the sample tube was filled with the sand unit in the front end and the organic-rich unit in the back end. The contact within the 5 cm diameter sample tube started about 8 cm in at the bottom and continued diagonally to about 16 cm in at the top. Although a field gamma spectrometry measurement was taken for this sample, the detector of the instrument is at the end of the device and was completely within the organic rich unit at the back of the sampling hole, whilst the sample used for dating was the sand unit at the front of the tube (staying at least 1 cm away from the contact). The gamma dose rate for sample KF16 can be modelled by assuming a planar boundary between the overlying sand and the underlying peat and applying a simple layer model as outlined in Appendix H of Aitken (1985). The implementation of this approach by Riedesel and Autzen (2020) was used, with the concentrations of U, Th and K for the two subsamples derived from ICP-MS measurements, and assuming a water content of 25% for the sand and 65% for the peat (a water content of 61% was measured for a sample collected from a fresh face of the organic-rich unit), and an average distance of the sample from the interface of 6 cm. This yields a gamma dose rate of 0.61 ± 0.10 Gy/ka for this sample and is used for age calculation.

Measured beta dose rates were corrected for attenuation due to grain size using the attenuation factors of Guérin et al. (2012) as well as corrected for attenuation by the assumed water content. Beta dose rates for the quartz fractions were also adjusted for removal of 8-10 µm of the outer surface of the grains by HF etching treatment using the etch depth attenuation factors of Bell (1979). The external alpha dose to quartz fractions were assumed to have been removed by the HF etching process. Many studies have shown that traces of uranium and thorium can be found within quartz grains, and an internal alpha dose rate of 0.030 ± 0.005 Gy/ka was added to the quartz dose rates, based on taking the mean of values measured by Jacobs et al. (2008), Clarkson et al. (2017) and Tooth et al. (2022).

Potassium rich feldspar fractions of the samples were not etched with HF since Duller (1992) suggested that the acid preferentially etches along twinning planes, rather than simply removing the alpha-irradiated outer surface of the grains. External alpha dose rates for these samples were calculated from the uranium and thorium concentrations derived from TSAC and attenuated for grain size (Brennan et al. 1991), water content and luminescence efficiency (a-value). Unfortunately, the a-value of coarse grain feldspar is poorly constrained. Several of the coarse grain feldspar a-values that are reported in published literature (e.g. 0.1 Aitken 1998; 0.2 Kolstrup and Mejdahl 1986) are only assumed values without underlying data measurements. The a-value used in this study is 0.11 ± 0.03 from Balescu and Lamothe (1993). Although the underlying data is not provided for this value and it is based on an IRSL protocol (not post-IR IRSL), it is reportedly based upon a-value measurements for two samples using

^{244}Cm and ^{60}Co as alpha and gamma sources and also agrees well with fine-grained polymineral a-value estimates reported for the post-IR IRSL signal (e.g. Kreutzer et al. 2014).

In addition to the external alpha dose rate, the potassium rich feldspar fractions also have an internal beta dose rate component due to the potassium content of the mineral grains. Many of the samples dated in this study have limited mass of separated K-feldspar material, but the internal potassium content for two of the samples was measured using the GM-25 beta counter, calibrated using the NIST 70a feldspar standard. The separated K-feldspar for sample KF16 was measured to have 11.5 ± 1.9% potassium and sample KF17 had 12.4 ± 1.2% potassium. Both measurements are consistent with the 12.5 ± 0.5% internal potassium content estimate from Huntley and Baril (1997), which is frequently used for coarse grain potassium rich feldspar fractions with unknown internal potassium content and is the assumed value used for calculating dose rates of all feldspar samples in this study.

Dose rates for the potassium-rich feldspar fractions of the eight sediment samples re-processed from the Duller et al. (2015) study were calculated using the same dosimetry measurements reported in the original publication. The external beta dose rate was calculated for the situation of no HF etching, and the external alpha and internal beta dose rate contributions were added based on the same assumptions regarding the a-value and K content as were made for the samples collected for this new Kalambo Falls site (Table 2.3). These new dose rate calculations were also computed in DRAC version 1.2 (Durcan et al. 2015).

Table 2.2: Calculated dose rate data for samples collected at Site BLB, Kalambo Falls, in 2019.

					Feldspar				Quartz	
Sample[a] (Aber232/)	Depth (m)	Grain Size (µm)	Cosmic (Gy/ka)	Gamma (Gy/ka)	External Beta (Gy/ka)	External Alpha (Gy/ka)	Internal Beta (Gy/ka)	Total Dose Rate (Gy/ka)	External Beta (Gy/ka)	Total Dose Rate (Gy/ka)[b]
KF01	9.3 ± 0.1	180-212	0.08 ± 0.01	0.48 ± 0.03	0.44 ± 0.03	0.06 ± 0.02	0.70 ± 0.07	1.76 ± 0.08		
KF02	8.7 ± 0.1	180-250	0.09 ± 0.01	0.47 ± 0.03	0.70 ± 0.04	0.10 ± 0.03	0.77 ± 0.14	2.13 ± 0.15		
KF04	2.6 ± 0.1	212-250	0.17 ± 0.02	0.57 ± 0.04	0.95 ± 0.06	0.07 ± 0.02	0.84 ± 0.08	2.59 ± 0.11	0.92 ± 0.06	1.68 ± 0.07
KF05	3.8 ± 0.1	180-250	0.15 ± 0.02	0.63 ± 0.05	0.76 ± 0.05	0.05 ± 0.01	0.77 ± 0.14	2.36 ± 0.16	0.73 ± 0.05	1.54 ± 0.07
KF06	4.7 ± 0.1	180-250	0.13 ± 0.01	0.65 ± 0.05	0.56 ± 0.04	0.04 ± 0.01	0.77 ± 0.14	2.15 ± 0.15	0.54 ± 0.04	1.35 ± 0.06
KF07	5.4 ± 0.1	180-250	0.12 ± 0.01	0.44 ± 0.03	0.42 ± 0.03	0.04 ± 0.01	0.77 ± 0.14	1.79 ± 0.15	0.41 ± 0.03	1.00 ± 0.04
KF08	6.4 ± 0.1	180-212	0.11 ± 0.01	0.64 ± 0.05	0.79 ± 0.05	0.12 ± 0.03	0.70 ± 0.07	2.36 ± 0.10	0.76 ± 0.05	1.54 ± 0.07
KF09	7.1 ± 0.1	180-250	0.10 ± 0.01	0.51 ± 0.03	0.36 ± 0.02	0.05 ± 0.01	0.77 ± 0.14	1.79 ± 0.15		
KF10	8.8 ± 0.1	180-212	0.09 ± 0.01	0.49 ± 0.03	0.57 ± 0.03	0.06 ± 0.01	0.70 ± 0.07	1.91 ± 0.09		
KF11	8.5 ± 0.1	180-212	0.09 ± 0.01	0.61 ± 0.04	0.49 ± 0.03	0.05 ± 0.01	0.70 ± 0.07	1.94 ± 0.09		
KF12	6.3 ± 0.1	180-212	0.11 ± 0.01	0.59 ± 0.04	0.67 ± 0.04	0.08 ± 0.02	0.70 ± 0.07	2.15 ± 0.09	0.64 ± 0.04	1.37 ± 0.06
KF13	7.6 ± 0.1	180-212	0.10 ± 0.01	0.52 ± 0.03	0.42 ± 0.03	0.06 ± 0.02	0.70 ± 0.07	1.80 ± 0.08		
KF14	8.5 ± 0.1	150-180	0.09 ± 0.01	0.41 ± 0.03	0.45 ± 0.03	0.06 ± 0.01	0.59 ± 0.06	1.59 ± 0.07		
KF15	9.1 ± 0.1	180-212	0.08 ± 0.01	0.37 ± 0.03[c]	0.46 ± 0.03	0.05 ± 0.01	0.70 ± 0.07	1.67 ± 0.08		
KF16	8.1 ± 0.1	180-212	0.09 ± 0.01	0.61 ± 0.10[d]	0.76 ± 0.05	0.05 ± 0.01	0.70 ± 0.07	2.22 ± 0.13[d]		
KF17	8.7 ± 0.1	180-212	0.09 ± 0.01	0.68 ± 0.04	0.74 ± 0.07	0.08 ± 0.02	0.70 ± 0.07	2.29 ± 0.11		

[a] Samples collected in 2019 all used a laboratory prefix of Aber232, so the full sample name is Aber232/KF01, Aber232/KF02 etc.

[b] Quartz dose rates are only reported for the six samples that were dated with OSL (the remaining 10 samples are older than OSL age range). Dose rates include an internal alpha dose of 0.030±0.005 Gy/ka.

[c] Sample KF15 had no field gamma measurement, the gamma dose rate is the average of the values calculated from concentrations of U, Th and K derived from alpha and beta counting data, and that calculated from concentrations of U, Th and K from ICP-MS.

[d] Sample KF16 cut across a significant unit boundary and the gamma dose rate was modelled using U, Th and K concentrations derived ICP-MS data for both units as described in the text

Table 2.3: Calculated dose rate data for potassium-feldspar fractions separated from samples collected at Site C North, Kalambo Falls, in 2005.

Sample [a] (Aber105/)	Grain Size (µm)	Cosmic (Gy/ka)	Gamma (Gy/ka)	External Beta (Gy/ka)	External Alpha (Gy/ka)	Internal Beta (Gy/ka)	Total Dose Rate (Gy/ka)
KB15	180-250	0.14 ± 0.01	0.43 ± 0.03	0.55 ± 0.04	0.04 ± 0.01	0.77 ± 0.14	1.94 ± 0.15
KB16	180-250	0.13 ± 0.01	0.39 ± 0.02	0.40 ± 0.03	0.05 ± 0.01	0.77 ± 0.14	1.73 ± 0.14
KB3	180-212	0.14 ± 0.01	0.78 ± 0.05	0.93 ± 0.06	0.10 ± 0.02	0.70 ± 0.07	2.66 ± 0.11
KB6	180-250	0.11 ± 0.01	0.53 ± 0.03	0.49 ± 0.03	0.08 ± 0.02	0.77 ± 0.14	1.98 ± 0.15
KB9	180-212	0.12 ± 0.01	0.56 ± 0.03	0.76 ± 0.05	0.07 ± 0.02	0.70 ± 0.07	2.21 ± 0.09
KB7	180-212	0.11 ± 0.01	0.49 ± 0.03	0.79 ± 0.04	0.08 ± 0.02	0.70 ± 0.07	2.17 ± 0.09
KB2	180-212	0.17 ± 0.01	0.61 ± 0.04	0.65 ± 0.04	0.07 ± 0.02	0.70 ± 0.07	2.20 ± 0.09
KB11	180-250	0.12 ± 0.01	0.35 ± 0.03	0.41 ± 0.03	0.04 ± 0.01	0.77 ± 0.14	1.70 ± 0.14

[a] Samples collected in 2005 all used a laboratory prefix of Aber105, so the full sample name is Aber105/KB2, Aber105/KB15 etc.

3. Equipment for Luminescence Measurements

Luminescence measurements were made on four Risø TL/OSL DA15 or DA20 readers (named Risø 1, Risø 3, Risø 4 and Risø 9) at Aberystwyth Luminescence Research Laboratory. These readers are equipped with Sr/Y beta sources delivering dose rates of 0.036, 0.067, 0.030 and 0.085 Gy/s, respectively. Beta source calibration is against a secondary gamma source at the Danish Technical University, Denmark. Each source has been calibrated between 6 and 8 times, over between 2 and 11 years, and all data used to determine the source strength. The factors recommended by Autzen et al. (2022) were used to reconcile measurements from calibration batches 17 to 200.

For luminescence measurements of quartz, grains were mounted in single grain discs containing 100 holes, and the OSL was stimulated using a single grain attachment based around a 10 mW Nd:YVO_4 laser emitting at 532 nm (Bøtter-Jensen et al., 2003). The light produced by this stimulation was filtered through 7.5mm U340 glass and measured with an EMI9635QA PMT. The beta source on the instrument used for measurement of single grains of quartz was selected for its uniformity. Gafchromic EBT2 film was used to measure the spatial variation in beta dose across a single grain disc and yielded a coefficient of variation across the 100 grains holes of 6%. Lapp et al (2012) used the same film to map two beta sources, one with a coefficient of variation of 25% and the other a value of 6%. They showed that for the source with 25% variation they could reduce the overdispersion of their equivalent dose (D_e) data by applying a grain-specific source calibration. However, for the source with 6% variation they saw no significant improvement in overdispersion. Since the source used here for quartz single

grain measurements also has a coefficient of variation of 6% we have not applied any grain-specific correction to the dose rate.

For potassium-rich feldspar fraction measurements, grains were mounted in small aliquots (~50 grains per aliquot) on flat aluminium discs using Silkospray silicone oil and the IRSL was stimulated using LEDs with power densities of 145 $mW.cm^{-2}$ (870 nm), 130 $mW.cm^{-2}$ (870 nm), and 270 $mW.cm^{-2}$ (850 nm), for readers Risø 1, Risø 3, and Risø 9 respectively. The duration of the IRSL stimulation was constant between readers regardless of LED power intensity. The resulting IRSL signal was filtered through a Schott BG-39 filter to reject the infrared stimulation, and either a Schott BG3 or a Corning 7-59 filter used to isolate the blue emission (~400 nm), and measured with a PMT. For a subset consisting of the 4 youngest samples from site BLB, single grain pIR-IRSL measurements were made of the potassium-rich feldspar fraction. Grains were mounted in single grain discs containing 100 holes, and IRSL and pIR-IRSL signals were stimulated using a 150 mW 830 nm laser (Bøtter-Jensen et al., 2003) coupled with an RG-780 filter to remove lower wavelength emissions. The optically stimulated luminescence produced by this stimulation was filtered through a combination of Schott BG-39 and Corning 7-59 filters and measured with an EMI9635QA PMT. The beta source on the instrument used for measurement of single grains of feldspar was selected for its uniformity, and measurement using Gafchromic EBT2 film yielded a coefficient of variation in dose rate across the 100 grains holes of 2%. Because of the uniformity of this source no grain-specific correction was applied (cf. Lapp et al. 2012).

4. Single grain quartz luminescence dating

Of the sixteen samples collected from Site BLB, only six are young enough to be dated using the quartz OSL signal. The quartz single grain measurements of this study follow the methods of Duller et al. (2015). The same single aliquot regenerative dose (SAR) protocol was used as in that study (Table 2.4) and the same acceptance criteria were applied, including recycling and IR depletion ratios (Duller 2003) within one sigma of 0.90 – 1.10 and a natural test dose signal that was at least three times greater than the standard deviation of the background signal. Six single grain discs were measured for each sample, each with a 10 by 10 grid of 300 µm diameter holes. As the grain sizes of the quartz used in this study were 180-250 µm it is possible that there was more than a single grain in some of these grain positions. Data from only 16-30% of the grain positions passed the acceptance criteria. The proportion of grains providing light is consistent with the study of Duller et al. (2015) which found ~24% of the quartz grains from Kalambo Falls yielded an OSL signal suitable for constructing a dose response curve.

Between 97 and 177 grains passed the rejection criteria for each sample (Table 2.5). For a small percentage of these grains, it was not possible to calculate an equivalent dose (D_e) because they were in saturation. Characteristic doses (D_0) for the quartz grains measured in this study ranged from 6.5 to 186 Gy, similar to previous observations on quartz from Kalambo (Duller 2012), so it was possible for grains to be saturated (estimated D_e value greater than $2D_0$) in even the youngest sample. Equivalent doses were calculated using the central age model (CAM; Galbraith et al. 1999) and overdispersion values ranged from 32-42% (Table 2.5). These

overdispersion values are similar to the 30-33% values of the single grain quartz samples that were interpreted to have been well bleached in the study by Duller et al. (2015).

Table 2.4: Protocol for equivalent dose determination from single grains of quartz. The experimental conditions listed in this table were chosen on the basis of the results of dose recovery tests conducted on sample 105/KB15 by Duller (2012).

Step	Procedure
1	Dose (X Gy)
2	Preheat 240°C at 5°C/s, and hold for 10s
3	OSL (532 nm stimulation for 4s, 25% full power) at 125°C. Sample heated at 5°C/s and held at 125°C for 15s prior to optical stimulation
4	Test Dose (5.6 Gy)
5	Preheat 220°C at 5°C/s and immediately cool
6	OSL (532 nm stimulation for 4s, 25% full power) at 125°C. Sample heated at 5°C/s and held at 125°C for 15s prior to optical stimulation
7	Return to step 1 and repeat for a series of doses

Table 2.5: Single grain luminescence dating results for quartz fractions from Site BLB.

Sample (Aber232/)	Measured	Number of grains Pass Criteria	Give D_e[a]	OD (%)	CAM D_e (Gy)	Dose Rate (Gy/ka)	Age (ka)[b]
KF04	600	177	176 (99%)	32	17.9 ± 0.5	1.68 ± 0.07	11 ± 1
KF05	600	149	149 (100%)	32	17.6 ± 0.5	1.54 ± 0.07	12 ± 1
KF06	600	152	147 (97%)	38	38.3 ± 1.4	1.35 ± 0.06	28 ± 2
KF07	600	123	118 (96%)	42	37.3 ± 1.6	1.00 ± 0.04	37 ± 2
KF08	600	170	159 (94%)	36	55.3 ± 1.8	1.54 ± 0.07	36 ± 2
KF12	600	97	86 (89%)	36	55.8 ± 2.4	1.37 ± 0.06	41 ± 3

[a] The number of accepted grains that yielded a D_e value. The value in parentheses is the proportion of the accepted grains that gave a D_e. The most common reason why grains passed the acceptance criteria but did not give a D_e is that their OSL signal was saturated, but as can be seen in the table, this was rare.

[b] Uncertainties (at 1 – sigma) shown for the ages listed here include both random and systematic sources of uncertainty

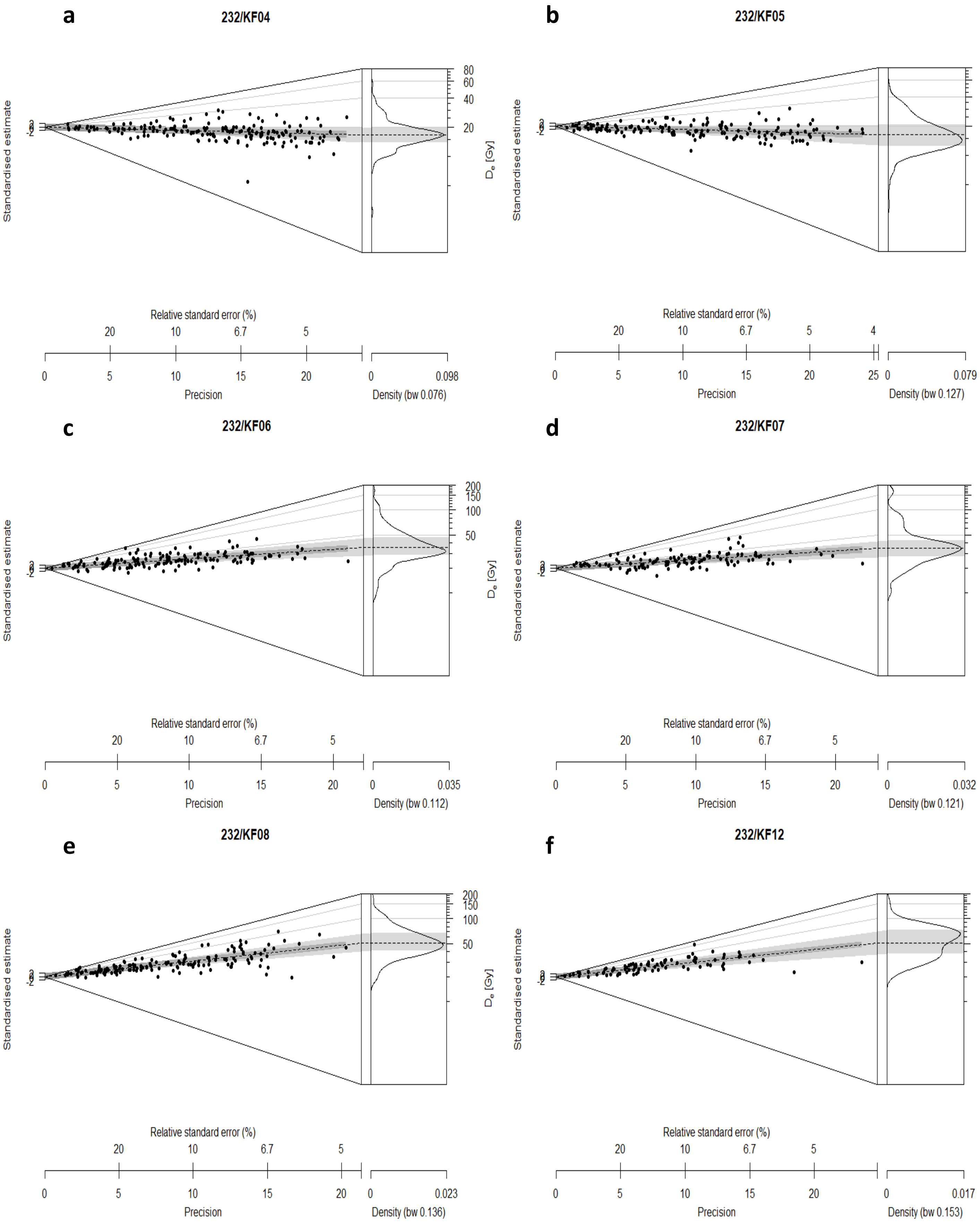

Figure 2.2: a – f, Abanico plots (Dietze et al., 2016) of the single grain quartz equivalent dose data for each of the six samples from Site BLB that were within quartz dating range. The grey band of each plot is centred on the weighted mean equivalent dose calculated using the CAM.

5. Feldspar Luminescence Dating

Feldspar luminescence dating has the potential to date older samples than quartz OSL dating but the phenomenon known as anomalous fading (Wintle 1973) may lead to age underestimations. However, the post-IR IRSL feldspar luminescence dating protocol (Thomsen et al. 2008) has been repeatedly demonstrated to be more stable with significantly reduced or even negligible fading rates when compared to the previous dating protocol in which IRSL measurements were made at 50°C (Li and Li 2011; Buylaert et al. 2012; Roberts 2012; Li et al. 2014).

5.1 post-IR IRSL Parameter Selection

The post-IR IRSL protocol has several parameters that are frequently varied between studies. These parameters include the temperatures used for the pre-heat, initial IR stimulation and the secondary IR stimulation, as well as the magnitude of the test dose. In this study we used a protocol with temperatures of 250 °C, 50 °C, and 225 °C for the preheat, initial IR stimulation, and secondary IR stimulation, respectively (Table 2.6). We also included long (30s) pauses at the stimulation temperature before the IR diodes were switched on in order to ensure more reproducible heating (Jain et al. 2007; Duller et al. 2020). Long post-IR IRSL stimulations (500s) were used for both L_x and T_x measurements to ensure sufficient removal of signal (Colarossi et al. 2018). The D_e value was first determined using one range-finder aliquot with a test dose of ~50 Gy and then all future measurements were made with a test dose of about 30% of the D_e measured for that range-finder aliquot as studies suggest test dose magnitudes of 15-80% of the D_e value are optimal for passing dose recovery tests and producing ages in agreement with independent age control (Yi et al. 2016). The initial range-finder aliquot was not used in final D_e calculations.

5.2 Post-IR IRSL Dose Recovery

To test the suitability of the post-IR IRSL protocol at this site, five samples with a range of equivalent doses from 26 to 760 Gy were selected for dose recovery tests using the protocol in Table 2.6. These dose recovery tests involved bleaching 6 newly made aliquots of a sample for 24-48 hrs in a SOL2 solar simulator and then giving 3 of the aliquots a known dose approximately equal to the sample-specific D_e. The remaining 3 aliquots were not given a dose and were used to measure the residual signal left after the SOL2 bleaching. This residual signal was then subtracted from the D_e values measured during the dose recovery tests. For the post-IR IRSL data, the signal was summed over the first 5 channels of stimulation (2.5 seconds), and a background calculated from the last 100 channels (50s) was subtracted. All the 5 samples passed the dose recovery tests within 5% of unity (Table 2.7).

Table 2.6: Post-IR IRSL protocol for equivalent dose determination using multiple grain aliquots of feldspar.

Step	Procedure
1	Dose (X Gy)
2	Preheat at 250°C, heating at 5°C/s and holding for 60s
3	IRSL measurement for 200s at 50°C
**4**	**IRSL measurement for 500s at 225°C**
5	Test Dose (~30% of D_e)
6	Preheat at 250°C, heating at 5°C/s and holding for 60s
7	IRSL measurement for 200s at 50°C
**8**	**IRSL measurement for 500s at 225°C**
9	Return to step 1 and repeat for a series of doses

Table 2.7: Post-IR IRSL dose recovery results for selected samples from Site BLB.

Sample (Aber232/)	Given dose (Gy)	Measured dose (Gy)	Residual dose (Gy)	Net Recovered dose (Gy)	Dose recovery ratio
KF02	760	742 ± 38	4.7 ± 0.4	737 ± 38	0.97 ± 0.05
KF04	26	28 ± 2	0.9 ± 0.2	27 ± 1	1.03 ± 0.06
KF11	681	660 ± 6	4.8 ± 0.3	655 ± 6	0.96 ± 0.01
KF12	114	115 ± 1	2.1 ± 0.1	113 ± 1	0.99 ± 0.01
KF14	681	666 ± 7	4.7 ± 0.1	661 ± 7	0.97 ± 0.04

5.3 Post-IR IRSL Fading Rate Measurements

Fading rates in this study were measured using the single aliquot approach described in Auclair and Lamothe (2003) with a dose of ~30 Gy for all samples regardless of the sample's equivalent dose. Fading rates were measured for 3 aliquots of each sample and included measurements after pauses of up to 2 days, including multiple measurements of the prompt signal and at least one other repeat point.

The fading rate of the post-IR IRSL signal measured for the 3 aliquots of each sample is given in Tables 2.8 and 2.9, and a summary of the fading rates measured for all 16 of the samples collected in 2019 (n=48 aliquots) and the 8 samples collected in 2005 (n=24 aliquots) is shown in Figure 2.3. There is no reason to think that there has been any change in the source material for the sediments at Kalambo, and so the variation in fading rates observed in Figure 2.3 is thought to reflect the difficulty of measuring this parameter. The best estimate of the fading rate for this material is the average value of all 72 aliquots, 1.46 ± 0.50 %/decade.

Thiel et al. (2011) suggested that below a certain fading rate, the values obtained may be laboratory artefacts. In their study, they observed that a field saturated polymineral sample (from below the Brunhes/Matuyama palaeomagnetic boundary) had a natural signal (L_n/T_n) that was in saturation with the laboratory dose response curve even though they measured a

fading rate of 1.1 ± 0.3 %/decade for the sample (for a dose of ~200 Gy). As a comparison, they also measured the fading rate of 3 aliquots of fine-grained quartz and obtained a measurement of 1.3 ± 0.3 %/decade (given dose not stated in their paper), and this observation has also been used to support the idea that low values of fading may be a laboratory artefact. The Thiel et al. (2011) study has since been referred to by many researchers as a reason for not correcting for fading where rates of less than 1.5 %/decade are observed. The average fading rate for the post-IR IRSL signal in the potassium-rich feldspar of samples from Kalambo Falls measured for a dose of 30 Gy is 1.46 ± 0.50 %/decade (Figure 2.3), which is just at the threshold suggested by Thiel et al. (2011). A discussion of whether the data should be corrected for fading or not is given in section 6.3.

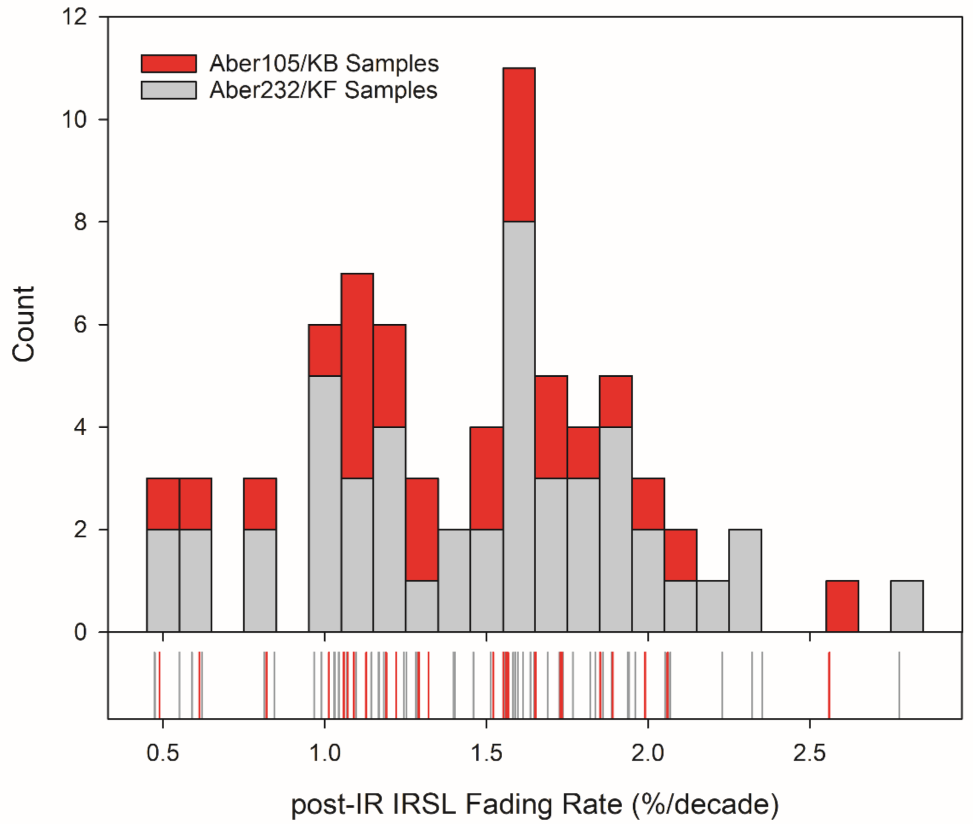

Figure 2.3:TT-TT Fading rates measured for the post-IR IRSL protocol (for a dose of ~30 Gy) for 72 aliquots in this study (3 aliquots per sample). Data are shown as a histogram and a rug plot for feldspars separated from 8 samples collected in 2005 (Aber105/KB samples) and 16 samples collected in 2019 (Aber232/KF samples). The average of all these measurements is 1.46 ± 0.50 %/decade.

5.4 Multiple Grain Post-IR IRSL Dating Results

To obtain D_e values for age determination, 12 small aliquots (~1 to 2 mm diameter coverage of the sample disc, containing ~50 grains per aliquot (Duller 2008)) were measured for each sample using the protocol in Table 2.6. The test dose magnitude was ~30% of the D_e value for each sample. A typical dose response curve is shown in Figure 2.4. The dose response curves were highly reproducible between aliquots, and the 12 replicate D_e values for each sample showed very little scatter, as illustrated by the low values of overdispersion (3-27%, Table 2.8). Abanico plots of the D_e values from each sample are shown in Figure 2.5 and 2.6. The Central Age Model (CAM) was used to combine the 12 replicate D_e measurements and obtain a single estimate used for age calculation.

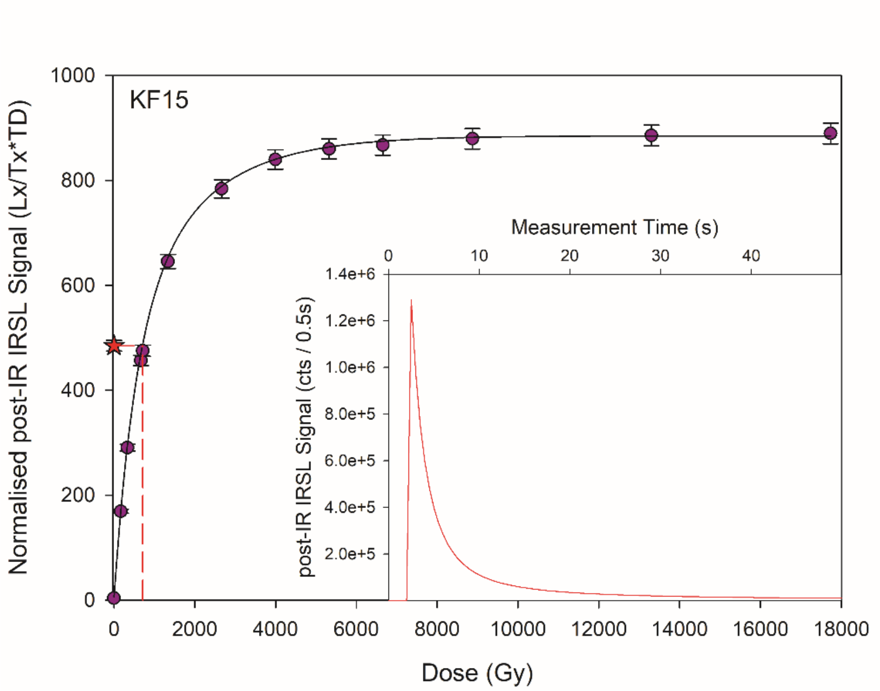

Figure 2.4: Representative dose response curve that was built up to very high doses using a small aliquot of sample KF15. The D_e of this aliquot was 738 ± 28 Gy and the L_n/T_n*T_D value is shown as a red star and interpolated on to the fitted function (sum of two exponentials). Each data point represents a single measurement. Uncertainty on each point is derived by combining in quadrature the uncertainty from counting statistics on the IRSL signal, with an instrumental uncertainty of 1.5%. Error bars are shown at 1σ. The inset shows the first 50 s of the post-IR IRSL natural signal of this aliquot (note that this measurement continued for an additional 450 seconds).

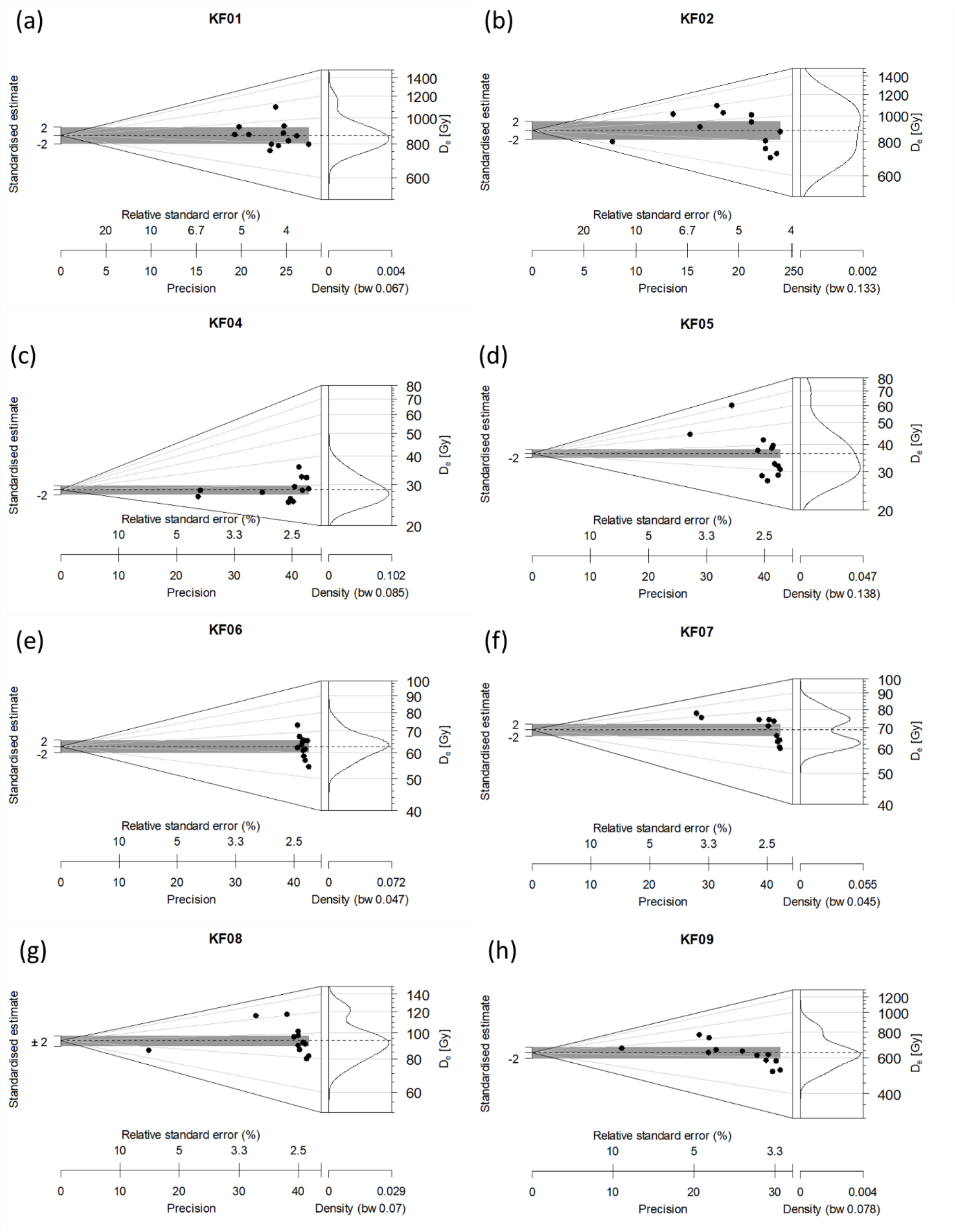
(a)
KF01
Standardised estimate
Relative standard error (%)
Precision
Density (bw 0.067)
De [Gy]
(b)
KF02
Standardised estimate
Relative standard error (%)
Precision
Density (bw 0.133)
De [Gy]
(c)
KF04
Standardised estimate
Relative standard error (%)
Precision
Density (bw 0.085)
De [Gy]
(d)
KF05
Standardised estimate
Relative standard error (%)
Precision
Density (bw 0.138)
De [Gy]
(e)
KF06
Standardised estimate
Relative standard error (%)
Precision
Density (bw 0.047)
De [Gy]
(f)
KF07
Standardised estimate
Relative standard error (%)
Precision
Density (bw 0.045)
De [Gy]
(g)
KF08
Standardised estimate
Relative standard error (%)
Precision
Density (bw 0.07)
De [Gy]
(h)
KF09
Standardised estimate
Relative standard error (%)
Precision
Density (bw 0.078)
De [Gy]

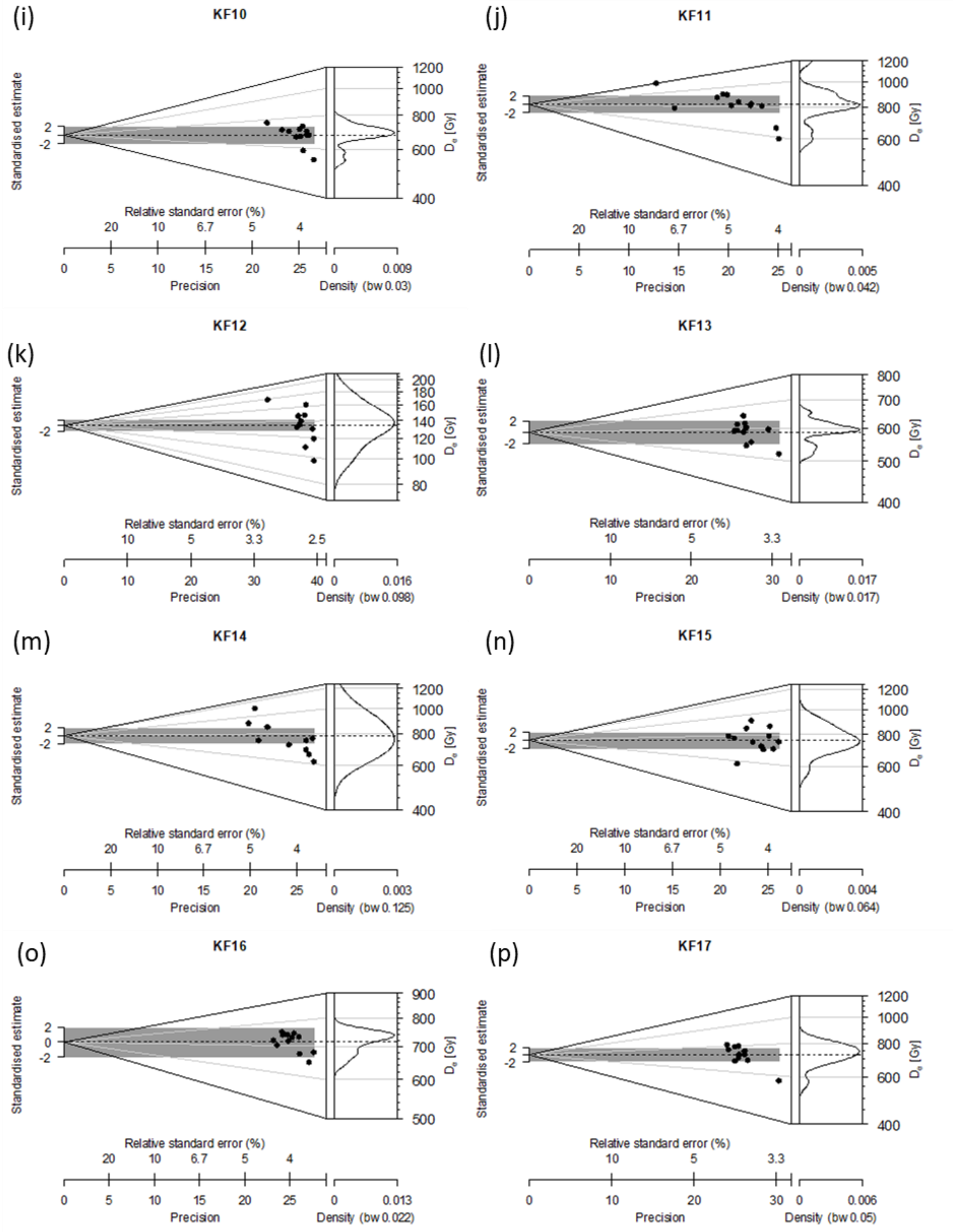

Figure 2.5: a – p, Abanico plots showing the distribution of post-IR IRSL D_e values for all samples collected from site BLB, Kalambo Falls, in 2019. The grey band of each plot is centred on the weighted mean equivalent dose calculated using the CAM.

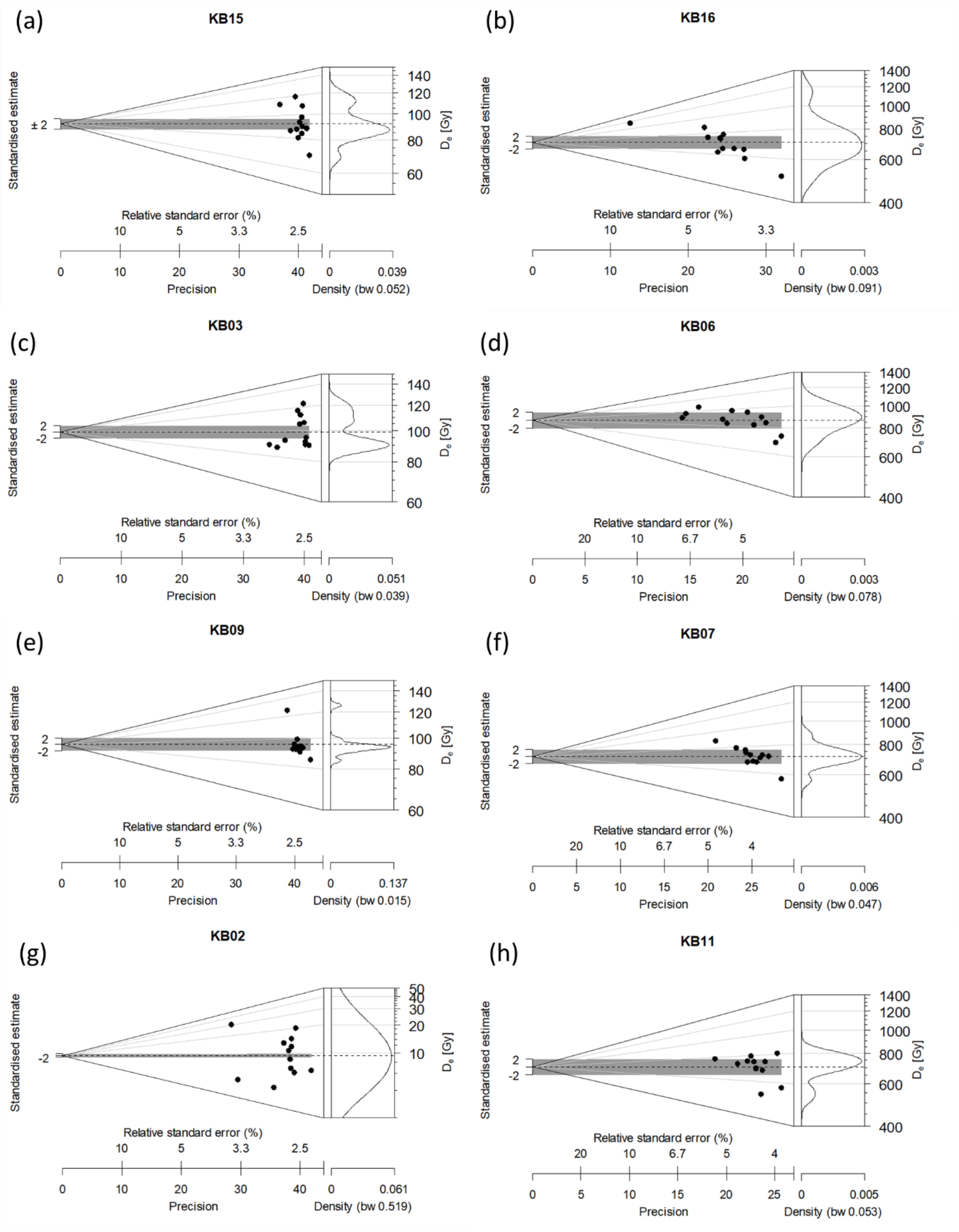

Figure 2.6: a – h, Abanico plots showing the distribution of post-IR IRSL D_e values for samples collected from site C North, Kalambo Falls, in 2005. The grey band of each plot is centred on the weighted mean equivalent dose calculated using the CAM.

Table 2.8: post-IR IRSL ages from the potassium-rich feldspar fractions extracted from samples collected at Site BLB, Kalambo Falls, in 2019.

Site	Sample (Aber232/)	Grain Size (µm)	Fading [a] %/decade	OD [b, c] (%)	CAM D_e [b] (Gy)	Dose Rate (Gy/ka)	Age [d] (ka)	Fading corrected [d, e] Age (ka)
BLB5	KF01	180-212	1.5 ± 0.2	11	859 ± 29	1.76 ± 0.08	487 ± 28 (20)	566 ± 44 (38)
	KF02	180-250	1.7 ± 0.1	18	884 ± 49	2.13 ± 0.15	416 ± 37 (34)	483 ± 52 (49)
BLB1	KF04	212-250	1.9 ± 0.3	11	29 ± 1	2.59 ± 0.11	11 ± 1 (1)	12 ± 1 (1)
	KF05	180-250	1.0 ± 0.3	27	36 ± 3	2.36 ± 0.16	15 ± 2 (1)	17 ± 2 (2)
	KF06	180-250	1.0 ± 0.0	8	63 ± 2	2.15 ± 0.15	29 ± 2 (2)	33 ± 3 (3)
	KF07	180-250	1.7 ± 0.2	10	69 ± 2	1.79 ± 0.15	38 ± 3 (3)	44 ± 5 (4)
	KF08	180-212	2.5 ± 0.3	15	94 ± 4	2.36 ± 0.10	40 ± 2 (2)	45 ± 4 (3)
	KF09	180-250	1.4 ± 0.5	15	634 ± 29	1.79 ± 0.15	355 ± 33 (30)	412 ± 45 (42)
BLB2	KF10	180-212	1.9 ± 0.1	8	676 ± 18	1.91 ± 0.09	355 ± 18 (12)	411 ± 32 (27)
	KF11	180-212	1.4 ± 0.3	15	818 ± 39	1.94 ± 0.09	422 ± 28 (22)	489 ± 43 (38)
BLB3	KF12	180-212	1.9 ± 0.3	16	134 ± 7	2.15 ± 0.09	62 ± 4 (3)	71 ± 6 (5)
	KF13	180-212	1.8 ± 0.2	5	587 ± 11	1.80 ± 0.08	326 ± 16 (10)	378 ± 29 (25)
	KF14	150-180	0.9 ± 0.6	15	783 ± 36	1.59 ± 0.07	492 ± 32 (25)	571 ± 51 (46)
	KF15	180-212	1.0 ± 0.5	11	758 ± 26	1.67 ± 0.08	453 ± 27 (20)	526 ± 44 (39)
BLB4	KF16	180-212	1.2 ± 0.1	3	714 ± 10	2.22 ± 0.13	322 ± 19 (15)	373 ± 31 (27)
	KF17	180-212	1.0 ± 0.3	8	724 ± 19	2.29 ± 0.11	316 ± 18 (12)	366 ± 28 (24)

[a] The fading rate is the g-value normalised to 2-days based on measurement of 3 aliquots per sample

[b] Twelve aliquots were measured to determine D_e for each sample, and all passed the acceptance criteria except one aliquot of KF12. The CAM was used to calculate the weighted mean and overdispersion

[c] Overdispersion (OD) is expressed as a percentage of the CAM D_e value.

[d] Uncertainties in brackets arise only from random sources, while those shown after the ± symbol combine both random and systematic sources. The systematic sources of uncertainty include 2% associated with laboratory beta source calibration, and additional sources associated with calibration of emission counting methods (alpha, beta and field gamma counting). All uncertainties shown at 1 – sigma.

[e] Ages corrected for anomalous fading using the average measured g-value of 1.46 ± 0.50 % per decade and using the approach of Huntley and Lamothe (2001).

Table 2.9: post-IR IRSL ages for the potassium-rich feldspar fractions separated from samples collected at Kalambo Falls Site C North in 2005.

Unit	Sample (Aber105/)	Grain Size (µm)	Fading [a] %/decade	OD [b, c] (%)	CAM D_e [b] (Gy)	Dose Rate (Gy/ka)	Age [d] (ka)	Fading corrected [d, e] Age (ka)
C	KB15	180-250	1.7 ± 0.1	14	92 ± 4	1.94 ± 0.15	47 ± 4 (4)	54 ± 6 (5)
	KB16	180-250	1.0 ± 0.3	19	706 ± 40	1.73 ± 0.14	408 ± 40 (37)	474 ± 53 (50)
C1	KB3	180-212	1.6 ± 0.4	12	99 ± 3	2.66 ± 0.11	37 ± 2 (1)	43 ± 3 (3)
	KB6	180-250	1.8 ± 0.9	11	865 ± 30	1.98 ± 0.15	437 ± 36 (32)	507 ± 51 (47)
C2	KB9	180-212	1.2 ± 0.3	9	95 ± 3	2.21 ± 0.09	43 ± 2 (1)	49 ± 4 (3)
	KB7	180-212	1.1 ± 0.1	10	716 ± 21	2.17 ± 0.09	330 ± 17 (10)	382 ± 29 (24)
C3	KB2	180-212	1.5 ± 0.2	62	9 ± 2	2.20 ± 0.09	4 ± 1 (1)	5 ± 1 (1)
	KB11	180-250	1.3 ± 0.7	12	702 ± 26	1.70 ± 0.14	413 ± 37 (33)	479 ± 51 (48)

[a] The fading rate is the g-value normalised to 2-days based on measurement of 3 aliquots per sample

[b] Twelve aliquots were measured to determine D_e for each sample with all passing acceptance criteria. The CAM was used to calculate the weighted mean and overdispersion.

[c] Overdispersion (OD) is expressed as a percentage of the CAM D_e value.

[d] Uncertainties in brackets arise only from random sources, while those shown after the ± symbol combine both random and systematic sources. The systematic sources of uncertainty include 2% associated with laboratory beta source calibration, and additional sources associated with calibration of emission counting methods (alpha, beta and field gamma counting). All uncertainties shown at 1 – sigma.

[e] Ages corrected for anomalous fading using the average measured g-value of 1.46 ± 0.50 % per decade and using the approach of Huntley and Lamothe (2001).

5.5 Single Grain Post-IR IRSL Measurements

Single grain measurements of quartz (section 4) demonstrated that the OSL signal had been well bleached at deposition. The pIR-IRSL signal from feldspars is reset by exposure to daylight more slowly than the OSL signal from quartz (Colarossi et al., 2015), so it is conceivable that the quartz OSL signal may be reset while some residual remains for the pIR-IRSL. To test whether this difference in resetting is observed at site BLB and a residual signal remained at deposition a set of single grain pIR-IRSL measurements were made on the four youngest samples. The same preheat and test dose parameters used for multiple grain measurements (Table 2.6) were used for single grain measurements (Table 2.10).

Data was screened to only accept those grains where the recycling ratio was within 10% of unity within uncertainties, the uncertainty on the test dose was less than or equal to 10%, the signal was more than three times the standard deviation of the background, and the dose response curve grew monotonically. This resulted in between 31 and 110 grains being accepted for each sample (Table 2.11). The central age model (Galbraith et al. 1999) was used to calculate the weighted mean D_e value. The ratio of the CAM value obtained using single grains to that using multiple grain measurements (Table 2.11) averages 1.02 ± 0.02. As expected, the overdispersion of the single grain pIR-IRSL D_e values (25 to 36%, Table 2.11)

are larger than the corresponding values from multiple grain measurements (8 to 27%, Table 2.8) because of the effects of averaging. However, the overdispersion values for the single grain pIR-IRSL measurements are lower than those from quartz (Table 2.5), and the distributions of D_e values derived from single feldspar grains (Figure 2.7) yield a single peak and do not exhibit the asymmetric pattern typical of incompletely bleached samples. The single grain pIR-IRSL D_e distributions imply that this signal was well bleached at deposition, meaning that either single grain or multiple grain measurements could be used. The multiple grain pIR-IRSL D_e values (Table 2.8 and 2.9) and the ages derived from these multiple grain measurements are used in subsequent discussions.

Table 2.10: Post-IR IRSL protocol for equivalent dose determination using single grains of feldspar.

Step	Procedure
1	Dose (X Gy)
2	Preheat at 250°C, heating at 5°C/s and holding for 60s
3	Single grain IRSL measurement for 2s at 50°C
**4**	**Single grain IRSL measurement for 2s at 225°C**
5	Test Dose (~30% of D_e)
6	Preheat at 250°C, heating at 5°C/s and holding for 60s
7	Single grain IRSL measurement for 2s at 50°C
**8**	**Single grain IRSL measurement for 2s at 225°C**
9	Return to step 1 and repeat for a series of doses

Table 2.11: Single grain luminescence dating results for feldspar fractions from Site BLB.

Sample	Number of grains		OD	CAM D_e	Ratio SG/MG [a]	Dose Rate	Age [b]
(Aber232/)	Measured	Pass Criteria	(%)	(Gy)	CAM D_e	(Gy/ka)	(ka)
KF04	300	50	29	29 ± 1	1.03 ± 0.06	2.59 ± 0.11	11 ± 1
KF05	300	31	36	32 ± 2	0.89 ± 0.09	2.36 ± 0.16	14 ± 1
KF06	300	113	25	65 ± 2	1.03 ± 0.04	2.15 ± 0.15	30 ± 2
KF07	300	110	25	69 ± 2	1.01 ± 0.04	1.79 ± 0.15	39 ± 3

[a] The ratio of the central age model D_e value based on single grain measurements to that based on multiple grain measurements.

[b] Uncertainties (at 1- sigma) shown for the ages listed here combine both random and systematic sources of uncertainty

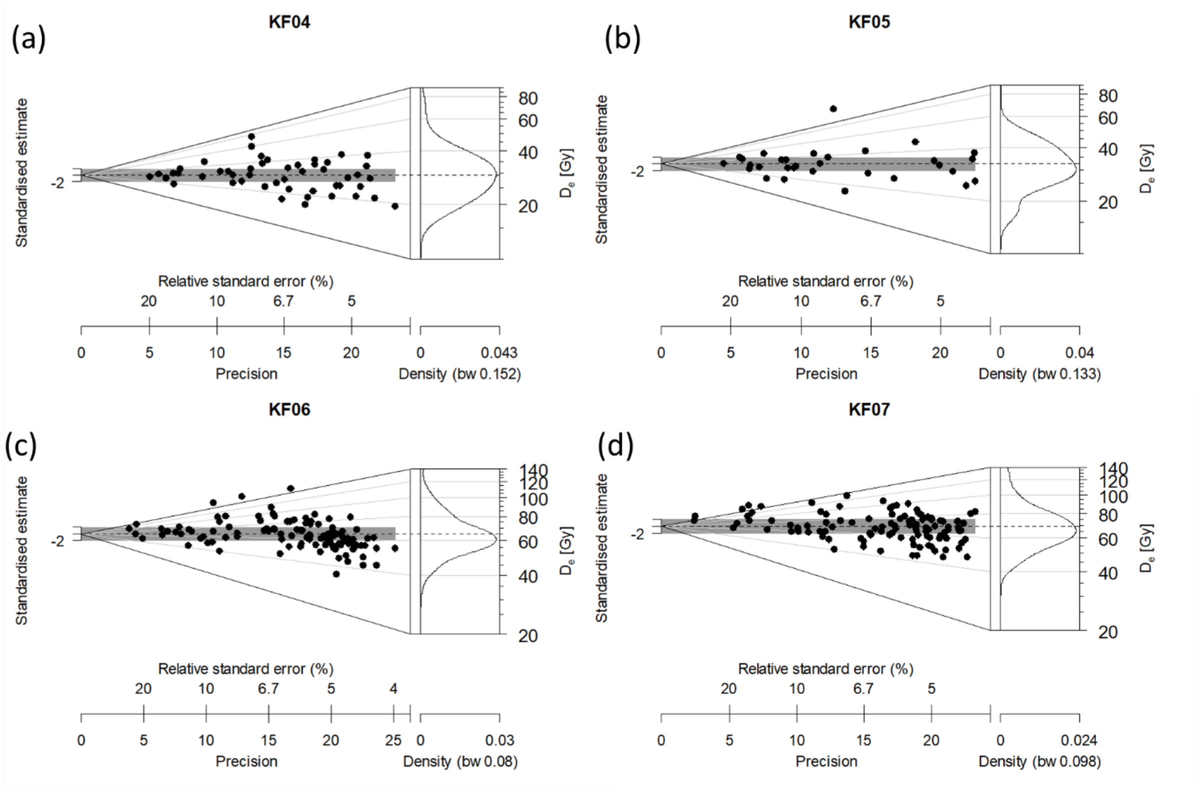

Figure 2.7: a – d, Abanico plots of single grain pIR-IRSL D_e values for the four youngest samples from site BLB. The grey band of each plot is centred on the weighted mean equivalent dose calculated using the CAM.

6. Discussion

Duller et al. (2015) provided a chronological framework for sediments collected in 2005 from Site C North, some ~100 m north (upstream) of Site BLB, based on single grain quartz OSL measurements for samples less than ~50 ka, and thermally-transferred OSL (TT-OSL) for older samples (up to ~300-500 ka). The use of single grain quartz OSL methods for dating sediments of 50 ka or less is well established (e.g. Jacobs et al. 2008), but the use of TT-OSL for dating the older sediments was challenging for two reasons. First the TT-OSL signal appeared not to have been fully reset at the time of deposition, and so a substantial residual signal (111 ± 5.6 Gy) had to be subtracted, with the assumption that this residual was the same for all samples. Second, the charge stored in the quartz grains that gives rise to the TT-OSL signal is thought to have a lifetime of only 4.5 million years at 10°C (Adamiec et al. 2010), and because of the high ambient temperature at this low latitude site, it was necessary to correct the apparent TT-OSL ages for this lifetime effect. Uncertainties in past temperatures at the site, and the antiquity of the samples, meant that the resulting TT-OSL ages had large uncertainties (typically ~20% of the age). For example, the oldest sample (KB11) gave an age of 532 ± 133 ka.

Recent developments in the use of feldspars for luminescence dating led to the decision to date the samples from Site BLB using the post-infrared infrared stimulated luminescence

(post-IR IRSL) signal. It is therefore important to assess whether luminescence ages calculated using single grain quartz OSL, quartz TT-OSL and feldspar post-IR IRSL are consistent with each other. To achieve this, single grain quartz OSL measurements were made for the 6 young samples from Site BLB where the OSL signal is under the limit of saturation and post-IR IRSL measurements were made for all 16 samples from Site BLB and a subset of 8 samples from Site C North. The comparison of these different approaches is discussed in the next sections.

6.1 Comparing single grain quartz OSL and feldspar post-IR IRSL ages

At Site BLB, the six samples with ages in the dating range of quartz OSL were dated using both the quartz single grain OSL approach and the feldspar post-IR IRSL method (Table 2.12). Additionally, four samples dated with quartz single grain OSL by Duller et al. (2015) at Site C North have been re-dated with post-IR IRSL in this study (Table 2.12). The agreement between the two sets of ages is excellent (Figure 2.8), with only a single sample from Site BLB (KF12 from unit BLB3) giving a post-IR IRSL age that is significantly older (62 ± 4 ka) than the corresponding quartz single grain OSL age (41 ± 3 ka). The single grain quartz analysis of KF12 gave the highest proportion of saturated grains (11%) of any of the samples from Site BLB, and so it is possible that this is causing the age to be underestimated. The post-IR IRSL age (4.2 ± 0.8 ka) for sample KB2 from Site C North is also older than its reported quartz age (0.76 ± 0.03 ka), however this sample was interpreted to be incompletely reset (Duller et al. 2015) and its quartz age was calculated using a minimum age model (Galbraith et al. 1999) on single grains of quartz while the post-IR IRSL age was calculated using the CAM on small aliquots of feldspar.

Table 2.12: Comparison of post-IR IRSL ages, with and without fading correction, with the quartz ages calculated for the same samples.

Unit	Sample	Grain Size (µm)	Post-IR IRSL Age (ka)	Fading Corr. Age [a]	Quartz Age (ka)[b]	Quartz OSL Method
BLB1	KF04	212-250	11 ± 1	12 ± 1	11 ± 1	SG OSL, this study
	KF05	180-250	15 ± 2	17 ± 2	12 ± 1	SG OSL, this study
	KF06	180-250	29 ± 2	33 ± 3	28 ± 2	SG OSL, this study
	KF07	180-250	38 ± 3	44 ± 5	37 ± 2	SG OSL, this study
	KF08	180-212	40 ± 2	45 ± 4	36 ± 2	SG OSL, this study
BLB3	KF12	180-212	62 ± 4	71 ± 6	41 ± 3	SG OSL, this study
C	KB15	180-250	47 ± 4	54 ± 6	43 ± 2	SG OSL, Duller et al. (2015)
	KB16	180-250	408 ± 40	474 ± 53	493 ± 117	TT-OSL, Duller et al. (2015)
C1	KB3	180-212	37 ± 2	43 ± 3	36 ± 2	SG OSL, Duller et al. (2015)
	KB6	180-250	437 ± 36	507 ± 51	416 ± 78	TT-OSL, Duller et al. (2015)
C2	KB9	180-212	43 ± 2	49 ± 4	47 ± 2	SG OSL, Duller et al. (2015)
	KB7	180-212	330 ± 17	382 ± 29	367 ± 53	TT-OSL, Duller et al. (2015)
C3	KB2	180-212	4 ± 1	5 ± 1	1 ± 0[c]	SG OSL, Duller et al. (2015)
	KB11	180-250	413 ± 37	479 ± 51	576 ± 144	TT-OSL, Duller et al. (2015)

[a] Fading corrections were made using the model of Huntley and Lamothe (2001) and the site average fading rate of 1.46 ± 0.50 %/decade

[b] Quartz ages from Duller et al. (2015) have been increased by 8.25% from the original publication due to an update to the secondary gamma source used for calibration (Autzen et al., 2022) and to make them comparable with other ages reported in this paper

[c] KB2 quartz age was calculated using the Minimum Age Model (Galbraith et al. 1999)

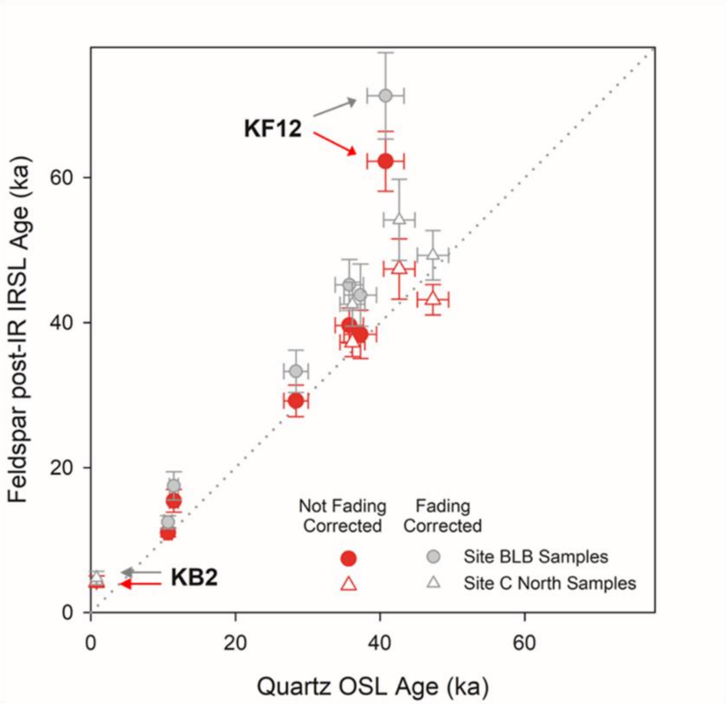

Figure 2.8: Comparison of single grain quartz OSL and feldspar post-IR IRSL ages. Samples from Site BLB are shown as circles, and those from Site C North as triangles. Grey symbols show fading corrected ages based on the site average fading rate of 1.46 ± 0.50 %/decade using the model of Huntley and Lamothe (2001). Each data point is one sample. For the quartz OSL ages between 97 and 177 grains were measured (Table 2.5), while 12 aliquots of each sample were measured for post-IR IRSL. Error bars on both sets of ages are at 1σ. Dotted line is the line of equivalence.

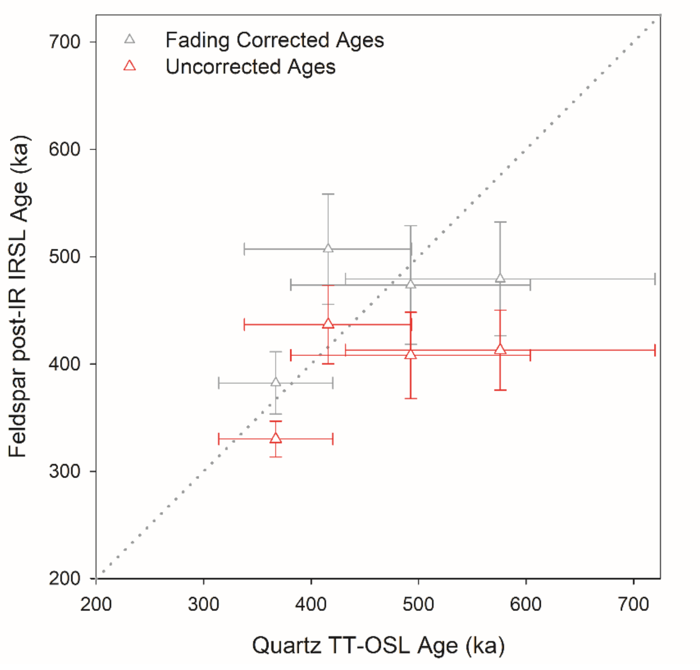

Figure 2.9: Comparison of feldspar post-IR IRSL ages measured in this study with quartz TT-OSL ages reported in Duller et al. (2015). Red symbols show feldspar post-IR IRSL ages without any fading correction while grey symbols show fading corrected ages based on the site average fading rate of 1.46 ± 0.50 %/decade using the model of Huntley and Lamothe (2001). Each data point is one sample. For the quartz TT - OSL ages 8 aliquots were measured while 12 aliquots of each sample were measured for post-IR IRSL. Error bars on both sets of ages are at 1σ. Dotted line is the line of equivalence.

6.2 Comparing quartz TT-OSL and feldspar post-IR IRSL ages

A set of 4 samples from Site C North (KB6, KB7, KB11, KB16) that were dated using the TT-OSL signal from quartz by Duller et al. (2015) have been re-dated using the post-IR IRSL signal from feldspars (Table 2.12). Figure 2.9 compares the ages obtained using these different chronometers. Given the large uncertainties on the TT-OSL ages (typically ~20%), the two sets of ages are consistent with each other. It is notable that the new post-IR IRSL ages for KB6, KB11 and KB16 give much more precise ages for deposition of the lowermost sediments exposed there than did the TT-OSL results.

6.3 Fading correction for post-IR IRSL ages

As discussed previously, luminescence from feldspars may be affected by anomalous fading (Wintle 1973), and one of the major recent innovations in the use of feldspars for dating is development of the post-IR IRSL method to reduce, and potentially eliminate, the impact of fading. Laboratory measurements of the fading rate (the ‘g-value’) described in Section 5.3 gave an average for the site of 1.46 ± 0.50 % per decade, almost identical to the threshold value

of 1.5 % per decade given by Thiel et al. (2011) above which some correction for fading should be undertaken.

Laboratory measurements of the g-value are hard to make and tend to be scattered. An alternative approach to assessing whether a correction is needed for fading is to compare the results obtained with another chronometer, one not affected by anomalous fading. In this study the quartz single grain OSL data set provides an independent dataset for comparison (Figure 2.8). When the post-IR IRSL ages are not corrected for fading, the average ratio of the feldspar age to quartz CAM age is 1.13 ± 0.06 (1.04 ± 0.03 if one excludes KF12). In contrast, when the post-IR IRSL ages were corrected for fading using the site average g-value of 1.46 ± 0.50 %/decade and the correction model of Huntley and Lamothe (2001) implemented in R (Kreutzer 2020), the fading corrected ages systematically lie above the 1:1 line, and the ratio of feldspar to quartz CAM ages is 1.28 ± 0.07 (1.19 ± 0.04 if one excludes KF12). It is conceivable that for these young samples (< ~60 ka), the post-IR IRSL D_e values may contain some residual signal if they were not completely bleached at deposition. However, single grain post-IR IRSL measurements of the youngest samples at site BLB (section 5.5 and Figure 2.7) do not exhibit any evidence for such incomplete bleaching. The closer agreement between quartz single grain OSL ages and post-IR IRSL without any fading correction suggest that no fading correction should be applied at this site.

The TT-OSL ages also provide an independent chronometer against which to compare the post-IR IRSL data and hence assess whether fading correction should be applied or not. Figure 2.9 shows the result of correcting ages for anomalous fading. However, the result of this comparison is not as clear as for the younger samples because of the large scatter on the TT-OSL ages, the dependence of the TT-OSL ages on a complex correction for lifetime of the TT-OSL signal and the subtraction of a large residual dose.

It is hard to conclusively decide whether the post-IR IRSL ages for Kalambo Falls should be corrected for fading or not. Ages both prior to correction for fading and after fading correction are presented in Table 2.8 and Table 2.9. The ages without fading correction have been used through the main text as this is a conservative estimate of the age. If a correction for fading were to be applied to these post-IR IRSL ages then they would be older than those given here, thus reinforcing the antiquity of the artefacts found at Kalambo Falls.

6.4 Age of sediments at Site BLB and Site C North, Kalambo Falls

The luminescence ages at Site BLB provide a means for understanding the sedimentary record preserved, and for placing the artefacts into a solid chronological framework. The consistency between the single grain OSL and the post-IR IRSL ages is excellent and the uncertainties on the post-IR IRSL ages are significantly lower than the TT-OSL ages published in 2015 (Figure 2.9). The ages are also less dependent on modelling and assumptions about long-term burial conditions, highlighting the advantages of using the potassium-rich feldspar post-IR IRSL dating protocol over the quartz TT-OSL protocol at this site.

References

Adamiec G., Duller G.A.T., Roberts H.M. & Wintle A.G. Improving the TT-OSL SAR protocol through source trap characterisation. *Radiation Measurements* **45**: 768-777. (2010)

Aitken M. J. Thermoluminescence Dating. London Academic Press, 359pp. (1985)

Aitken M. J. An Introduction to Optical Dating: The Dating of Quaternary Sediments by the Use of Photon-Stimulated Luminescence. Oxford University Press, 267pp. (1998)

Auclair M., Lamothe M. and Huot S. Measurement of anomalous fading for feldspar IRSL using SAR. *Radiation Measurements* **37**(4-5): 487-492. (2003)

Autzen M., Andersen C.E., Bailey M. and Murray A.S. Calibration quartz: An update on dose calculations for luminescence dating. *Radiation Measurements* **157**: 106828. (2022)

Balescu S. and Lamothe M. Thermoluminescence dating of the Holsteinian marine formation of Herzeele, northern France. *Journal of Quaternary Science* **8**: 117-124. (1993)

Barham L., Tooth S., Duller G.A.T., Plater A.J. and Turner S. Excavations at Site C North, Kalambo Falls, Zambia: new insights on the Mode 2/3 transition in south-central Africa. *African Journal of Archaeology* **13**(2): 187-214. (2015)

Bell, W.T. Attenuation factors for the absorbed radiation dose in quartz inclusions for thermoluminescence dating. *Ancient TL* **8**: 1-12. (1979)

Bøtter-Jensen L. and Mejdahl V. Assessment of beta dose-rate using a GM multicounter system. *Nuclear Tracks and Radiation Measurements* **14**: 187-191. (1988)

Bøtter-Jensen L., Andersen C.E., Duller G.A.T. and Murray A.S. Developments in radiation, stimulation and observation facilities in luminescence measurements. *Radiation Measurements* **37**: 535-541. (2003)

Brennan B.J., Lyons R.G. and Phillips S.W. Attenuation of alpha particle track dose for spherical grains. International Journal of Radiation Applications and Instrumentation. Part D. *Nuclear Tracks and Radiation Measurements* **18**: 249-253. (1991)

Buylaert J-P., Jain M., Murray A.S., Thomsen K.J., Thiel C. and Sohbati R. A robust feldspar luminescence dating method for Middle and Late Pleistocene sediments. *Boreas* **41**(3): 435-451. (2012)

Clarkson C., Jacobs Z., Marwick B., Fullagar R., Wallis L., Smith M., Roberts R.G., Hayes E., Lowe K., Carah X., Florin S.A., McNeil J., Cox D., Arnold L.J., Hua Q., Huntley J., Brand H.E.A., Manne T., Fairbairn A., Shulmeister J., Lyle L., Salinas M., Page M., Connell K., Park G., Norman K., Murphy T. and Pardoe C. Human occupation of northern Australia by 65,000 years ago. *Nature* **547**: 306-310. (2017)

Colarossi D., Duller G.A.T., Roberts H.M., Tooth S. and Lyons R. Comparison of paired quartz OSL and feldspar post-IR IRSL dose distributions in poorly bleached fluvial sediments from South Africa. *Quaternary Geochronology* **30**: 233-238. (2015)

Colarossi D., Duller G.A.T. and Roberts H.M. Exploring the behaviour of luminescence signals from feldspars: Implications for the single aliquot regenerative dose protocol. *Radiation Measurements* **109**: 35-44. (2018)

De Corte F., Vandenberghe D., Hossain S.F., De Wispelaere A., Buylaert J.-P. & Van den Haute P. Preparation and characterization of loess sediment for use as a reference material in the annual radiation dose determination for luminescence dating. *Journal of Radioanalytical and Nuclear Chemistry* 272: 311-319. (2007)

Dietze M., Kreutzer S., Burow C., Fuchs MC., Fischer M. and Schmidt C. The Abanico plot: Visualising chronometric data with individual standard errors. *Quaternary Geochronology* **31**: 12-18. (2016)

Duller G.A.T. Luminescence chronology of raised marine terraces, south-west North Island, New Zealand. Unpublished PhD thesis, University of Wales, Aberystwyth. (1992)

Duller G.A.T. Distinguishing quartz and feldspar in single grain luminescence measurements. *Radiation Measurements* **37**: 161-165. (2003)

Duller G.A.T. Single grain optical dating of Quaternary sediments: why aliquot size matters in luminescence dating. *Boreas* **37**: 589-612. (2008)

Duller G.A.T. Improving the accuracy and precision of equivalent doses determined using the optically stimulated luminescence signal from single grains of quartz. *Radiation Measurements* **47**: 770-777. (2012)

Duller G.A.T. and Wintle A.G. The potential of the thermally transferred optically stimulated luminescence signal from quartz for dating sediments. *Quaternary Geochronology* **7**: 6-20. (2012)

Duller G.A.T., Tooth S., Barham L. and Tsukamoto S. New investigations at Kalambo Falls, Zambia: Luminescence chronology, site formation, and archaeological significance. *Journal of Human Evolution* **85**: 111-125. (2015)

Duller G.A.T., Roberts H.M. and Pinder R.C. A method for routinely monitoring the reproducibility of thermal pretreatment prior to optically stimulated luminescence measurements. *Radiation Measurements* **13**0: 106210. (2020)

Durcan J.A., King G.E. and Duller G.A.T. DRAC: dose rate and age calculator for trapped charge dating. *Quaternary Geochronology* **28**: 54-61. (2015)

Galbraith R.F., Roberts R.G., Laslett G.M., Yoshida H. and Olley J.M. Optical dating of single and multiple grains of quartz from Jinmium rock shelter, northern Australia: Part I, Experimental design and statistical models. *Archaeometry* **41**: 339-364. (1999).

Guérin G., Mercier N., Nathan R., Adamiec G. and Lefrais Y. On the use of the infinite matrix assumption and associated concepts: A critical review. *Radiation Measurements* **47**: 778-785. (2012)

Hansen V., Murray A., Buylaert J-P., Yeo E-Y. and Thomsen K. A new irradiated quartz for beta source calibration. *Radiation Measurements* **81**: 123-127. (2015)

Huntley D.J. and Baril M.R. The K-content of the K-feldspars being measured in optical dating or in thermoluminescence dating. *Ancient TL* **15**: 11-13. (1997)

Huntley D.J. and Lamothe M. Ubiquity of anomalous fading in K-feldspars and the measurement and correction for it in optical dating. *Canadian Journal of Earth Sciences* **38**: 1093-1106. (2001)

Jacobs Z., Roberts R.G., Galbraith R.F., Deacon H.J., Grun R., Mackay A.W., Mitchell P., Vogelsang R. and Wadley L. Ages for the Middle Stone Age of Southern Africa: Implications for human behavior and dispersal. *Science* **322**: 733-735. (2008)

Jain M., Bøtter-Jensen L., Murray A.S. and Essery R. A peak structure in isothermal luminescence signals in quartz: Origin and implications. *Journal of Luminescence* **127**(2): 678-688. (2007)

Kolstrup E. and Mejdahl V. Three frost wedge casts from Jutland (Denmark) and TL dating of their infill. *Boreas* **15**: 311-321. (1986)

Kreutzer S., Schmidt C., DeWitt R. and Fuchs M. The a-value of polymineral fine grain samples measured with the post-IR IRSL protocol. *Radiation Measurements* **69**: 18-29. (2014)

Kreutzer S. calc_FadingCorr: Apply a fading correction according to Huntley & Lamothe (2001) for a given g-value and a given tc. Function version 0.4.2. In: Kreutzer, S., Burow, C., Dietze, M., Fuchs, M.C., Schmidt, C., Fischer, M., Friedrich, J., Riedesel, S., Autzen, M., Mittelstrass, D., 2020. Luminescence: Comprehensive Luminescence Dating Data Analysis. R package version 0.9.10. https://CRAN.R-project.org/package=Luminescence (2020)

Lapp T., Jain M., Thomsen K.J., Murray A.S. and Buylaert J.-P. New luminescence measurement facilities in retrospective dosimetry. *Radiation Measurements* **47**: 803-808. (2012)

Li B. and Li S.H. Luminescence dating of K-feldspar from sediments: A protocol without anomalous fading correction. *Quaternary Geochronology* **6**: 468-479. (2011)

Li B., Jacobs Z., Roberts R.G. and Li S.H. Review and assessment of the potential of post-IR IRSL dating methods to circumvent the problem of anomalous fading in feldspar luminescence. *Geochronometria* **41**: 178-201. (2014)

Mercier N. and Falguères C. Field gamma dose-rate measurement with a NaI(Tl) detector: re-evaluation of the "threshold" technique. *Ancient TL* **25**(1): 1-4. (2007)

Prescott J.R. and Hutton J.T. Cosmic ray contributions to dose rates for luminescence and ESR dating: Large depths and long-term time variations. *Radiation Measurements* **23**: 497-500. (1994)

Rhodes E.J. and Schwenninger J-L. Dose rate and radioisotope concentrations in the concrete calibration blocks at Oxford. *Ancient TL* **25**(1): 5-8. (2007)

Riedesel S. and Autzen M. Beta and gamma dose rate attenuation in rocks and sediment. *Radiation Measurements* **133**: 10. (2020)

Roberts H.M. Testing Post-IR IRSL protocols for minimising fading in feldspars, using Alaskan loess with independent chronological control. *Radiation Measurements* **47**(9): 716-724. (2012)

Tooth S., McCarthy T.S., Duller G.A.T., Assine M.L., Wolski P. and Coetzee G. Significantly enhanced mid Holocene fluvial activity in a globally important, arid-zone wetland: the Okavango Delta, Botswana. *Earth Surface Processes and Landforms* **47**(3): 854-871. (2022)

Thiel C., Buylaert J.P., Murray A.S., Terhorst B., Hofer I., Tsukamoto S., and Frechen M. Luminescence dating of the Stratzing loess profile (Austria) – Testing the potential of an elevated temperature post-IR IRSL protocol. *Quaternary International* **234**: 23-31. (2011)

Thomsen K.J., Murray A.S., Jain M. and Bøtter-Jensen L. Laboratory fading rates of various luminescence signals from feldspar-rich sediment extracts. *Radiation Measurements* **43**: 1474-1486. (2008)

Wintle A.G. Anomalous fading of thermoluminescence in mineral samples. *Nature* **245**: 143-144. (1973)

Yi S., Buylaert J.P., Murray A.S., Lu H., Thiel C. and Zeng L. A detailed post-IR IRSL dating study of the Niuyangzigou loess site in northeastern China. *Boreas* **45**: 644-657. (2016)

3. Identification of wood samples from Site BLB Kalambo Falls, Zambia

We describe the results of the identification of six samples of wood excavated in 2019 from the early levels of Kalambo Falls (excavation areas BLB3, BLB5, BLB2, BLB4). The identification is accompanied by information on the contemporary uses of the wood with relevance to the selection of the woods as raw materials. SEM analysis was used to examine the wood to discern the diagnostic cellular features for each taxon (whether to genus or species level). A sixth sample (from BLB3) is identified to genus level and although it shows no evidence of shaping its presence adds information about the local habitat.

Identification

SEM images for specimens from BLB2, BLB3 and BLB4.

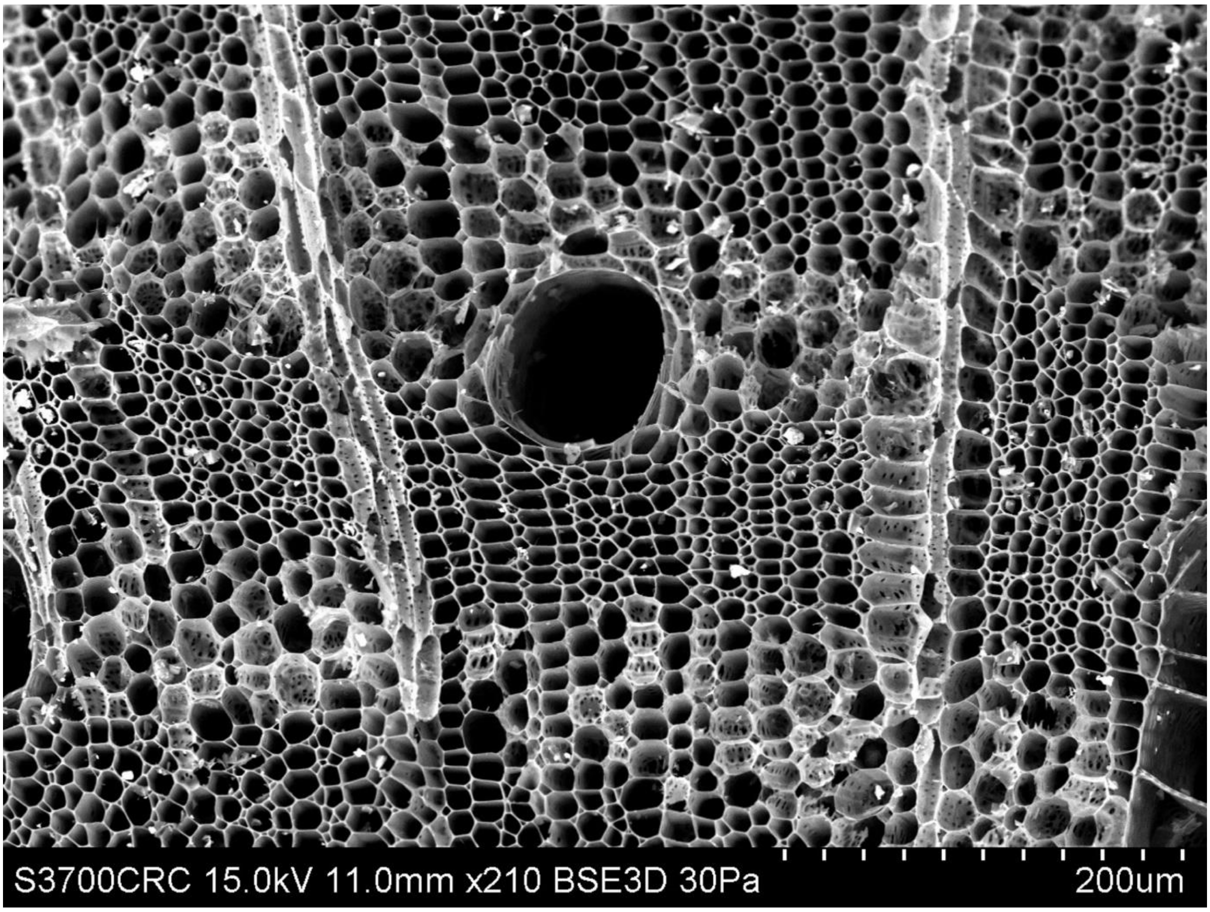

Fig. 3.1, BLB2, 'digging stick' (object 219). VP SEM image of a transverse section of *Kigelia africana* showing vasicentric, aliform, confluent and marginal parenchyma, rays 1 to 3 cells wide, and mostly solitary vessels. Scale bar in microns. Image: C.R. Cartwright; copyright The Trustees of the British Museum.

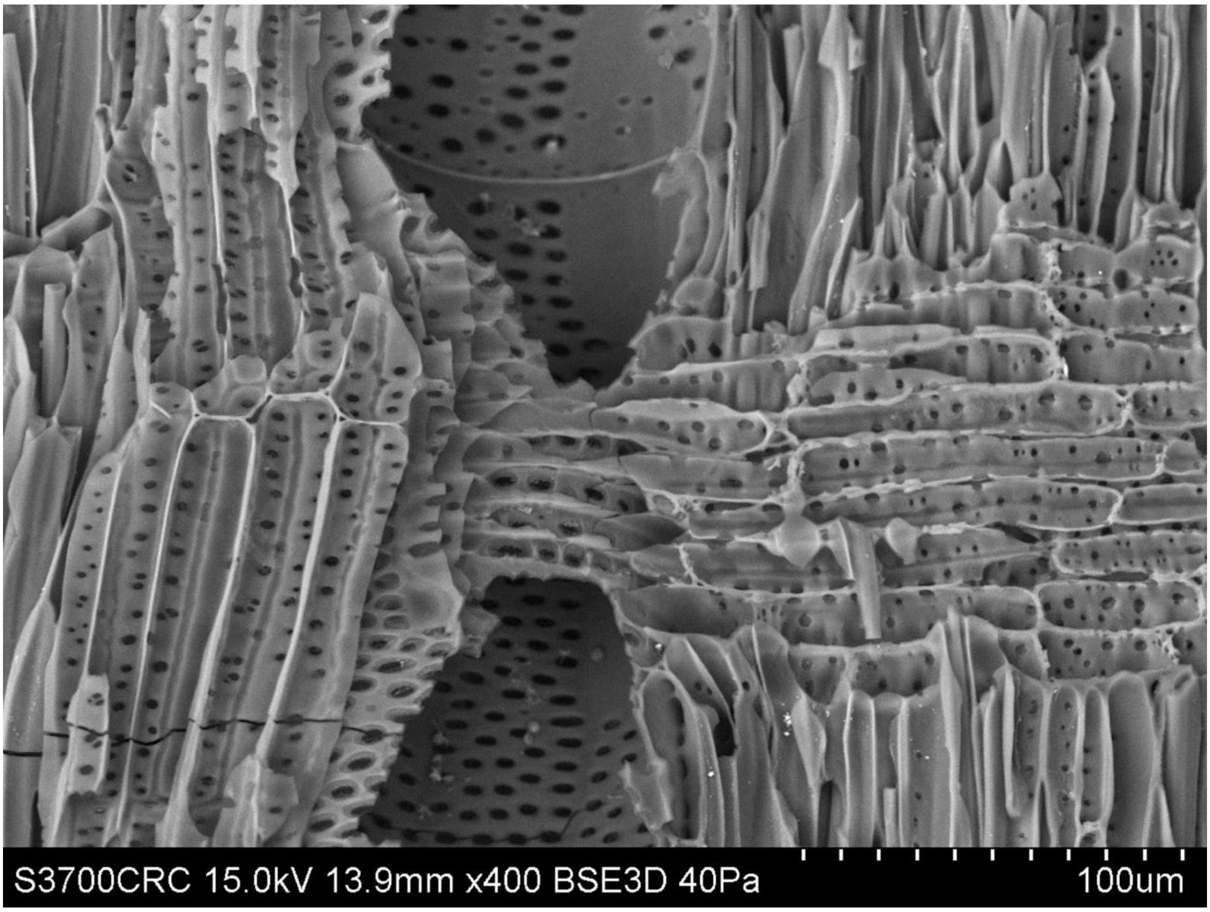

Fig.3.2, BLB-4 cut log. VP SEM image of a radial longitudinal section of *Combretum zeyheri* wood from the 'cut log', showing rays with procumbent body cells, and vessel-ray pits with distinct borders; similar to intervessel pits in size and shape throughout the ray cells. Scale bar in microns. Image: C.R. Cartwright; copyright The Trustees of the British Museum.

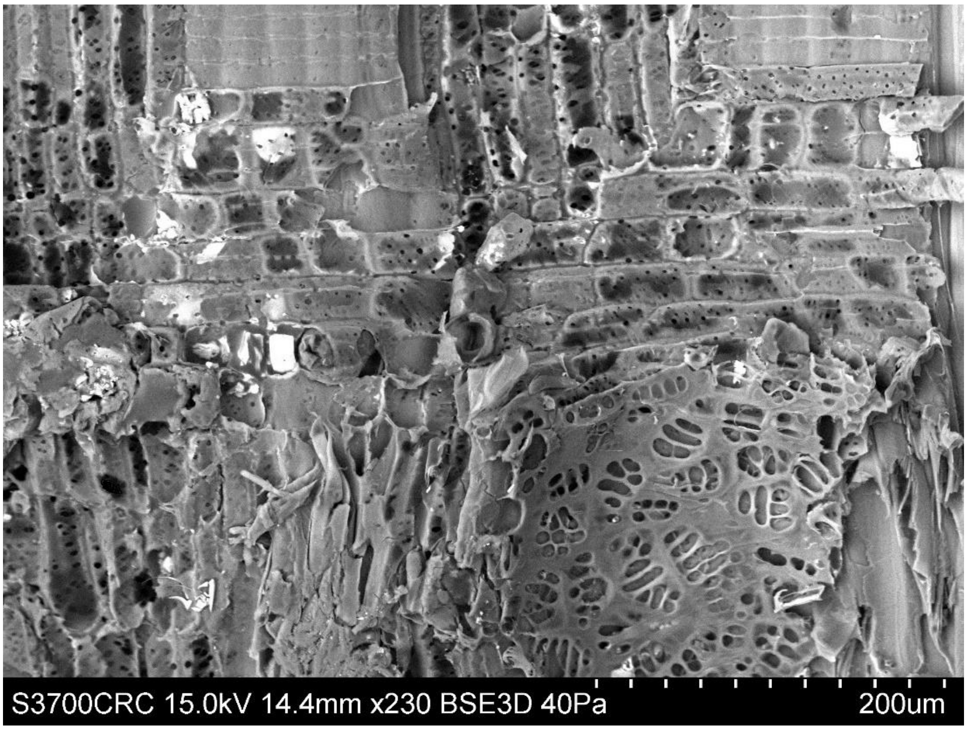

Fig. 3.3, BLB3, (object 661, unworked). VP SEM image of a radial longitudinal section of Ficus sp. wood from BLB3 (object 661), showing rays with procumbent body cells plus one row of upright and / or square marginal cells. Also present are vessel-ray pits with much reduced borders to apparently simple plus scalariform / gash-like horizontal pitting and palisade vertical pitting. Scale bar in microns. Image: C.R. Cartwright; copyright The Trustees of the British Museum.

Table 3.1. Summary of wood identifications.

**Sample**	**Object identifier**	**Wood identifications**
BLB-2 ‘digging stick’	Object 219	*Kigelia africana*
BLB-3 ‘wedge’	Object 660	*Kigelia africana*
BLB-3 ‘unmodified’	Object 661	*Ficus* sp.
BLB-4 ‘cut log’	Upper object	*Combretum zeyheri*
BLB-4 ‘notched branch’	Lower object	*Combretum zeyheri*
BLB-5 ‘structural log’	Object 1033	*Combretum zeyheri*

Contemporary uses

The wood samples BLB2 ‘digging stick’ (object 219) and BLB3 ‘wedge’ (object 660) are *Kigelia africana.* These trees inhabit woodland and riverine fringes. *Kigelia africana* has many traditional African medicinal uses and its fruits and bark are much sought after as food, browse and for a black dye from the tannin-rich fruit pulp (Grace and Davis 2002). Among the many uses for its quality wood are canoes, planks, posts, boxes, drums, stools, tools, mortars, bowls and bows. *Kigelia africana* wood is also used as fuel (Bussmann et al 2020)

Although the ‘unmodified sample’ wood sample from BLB3 (object 661) can only be identified to genus level not species, i.e. *Ficus* sp. (fig), several noteworthy points emerge from its presence. Numerous fig tree species are present in Zambia (Bingham et al 2020), including in the vegetational environmental around Kalambo Falls. Many fig tree species attain mature status when growing on nutrient-rich soils such as riverine alluvium (Burrows and Burrows 2003). As well as providing edible fruits, fig trees are known for traditional medicinal and phytopharmacological uses. Although fig wood can sometimes be soft and not high quality, certain fig species yield timber that is suitable for tools, mortars, boxes, drums and beehives (see, for example, Lumbile and Mogotsi 2008). It is also a valuable source of firewood.

Samples BLB5 ‘structural log’ (object 1033) and BLB4: cut log (upper object), ‘notched branch’ (lower object) are *Combretum zeyheri.* A widespread woodland species, *Combretum zeyheri* provides a general-purpose timber used at the present day for agricultural implements, chairs and fences as well as firewood. When seasoned, the wood is particularly durable. The leaves provide browse for livestock and game. Many parts of this tree have medicinal applications. Its fibrous roots may be used for basketry (Bingham et al 2020).

References

Bingham, M.G., Willemen, A., Wursten, B.T., Ballings, P. and Hyde, M.A. 2020 *Flora of Zambia* https://www.zambiaflora.com/ (2020)

Burrows, J.E. and Burrows, S.M. *Figs of Southern and South-Central Africa.* Umdaus Press, South Africa (2003)

Bussmann, R.W., Paniagua-Zambrana, N.Y., Njoroge, G.N. *Kigelia africana* (Lam.) Benth. Bignoniaceae. In: Bussmann, R.W. (eds.) *Ethnobotany of the Mountain Regions of Africa*. Ethnobotany of Mountain Regions. Springer, Cham. https://doi.org/10.1007/978-3-319-77086-4_97-1 (2020)

Cartwright, C.R. Identifying the woody resources of Diepkloof Rock Shelter (South Africa) using scanning electron microscopy of the MSA wood charcoal assemblages. *Journal of Archaeological Science* **40**; 3463-3474 http://www.sciencedirect.com/science/article/pii/S0305440312005560 (2013)

Cartwright, C.R. The principles, procedures and pitfalls in identifying archaeological and historical wood samples. *Annals of Botany* **116** (1); 1-13. http://aob.oxfordjournals.org/content/116/1/1.full.pdf+html?sid=4f94de01-bdf1-4496-947b-c9d35981f8da (2015)

Cartwright, C. R. and Parkington, J.E. The wood charcoal assemblages from Elands Bay Cave, southwestern Cape: principles, procedures and preliminary interpretation. *South African Archaeological Bulletin* **52** 59-72 (1997)

Grace, O.M. and Davis, S.D. *Kigelia africana* (Lam.) Benth. In: Oyen, L.P.A. and Lemmens, R.H.M.J. (eds) PROTA (Plant Resources of Tropical Africa / Ressources végétales de l'Afrique tropicale), Wageningen, Netherlands. https://uses.plantnet-project.org/en/Kigelia_africana_(PROTA) (2002)

Lumbile, A.U. and Mogotsi, K.K. *Ficus sur* Forssk. In: Louppe, D., Oteng-Amoako, A.A. and Brink, M. (eds). PROTA (Plant Resources of Tropical Africa / Ressources végétales de l'Afrique tropicale), Wageningen, Netherlands (2008)

4. Wood anatomy of modified objects from Site BLB, Kalambo Falls, Zambia

Macroscopic examination of the wooden objects and architectural elements from Kalambo Falls focused on the observation of characteristics to detect the likely position of the shaped wooden items on the tree (Figure 4.1). To this end, we recorded the presence and state of the preservation (e.g., partial) of bark, the direction of the radial and/or the tangential longitudinal grain of wood on preserved external surfaces of the objects. Considering the macroscopic and microscopic assessments carried out using SEM, we propose that BLB 5 (object 1033) and BLB4 (upper object, ‘cut log’) are derived from mature trunk wood. As an alternative, BLB4 (upper object, ‘notched branch’) may have been derived from a buttress near the base of the tree. The objects from BLB2 (219) and BLB4 (lower) represent juvenile, branch wood. BLB4 (lower) further appears to have been split radially on the thicker end (e.g., through/close to the pith), exposing the radial longitudinal plane of wood. On several items preserved bark is very visible, for example on half of the outer surface of BLB3 (object 660). The same object additionally demonstrates the clearly visible transverse plane at its base (where impact damage is observed), and the exposed tangential longitudinal plane on the surface where there is evidence for reduction.

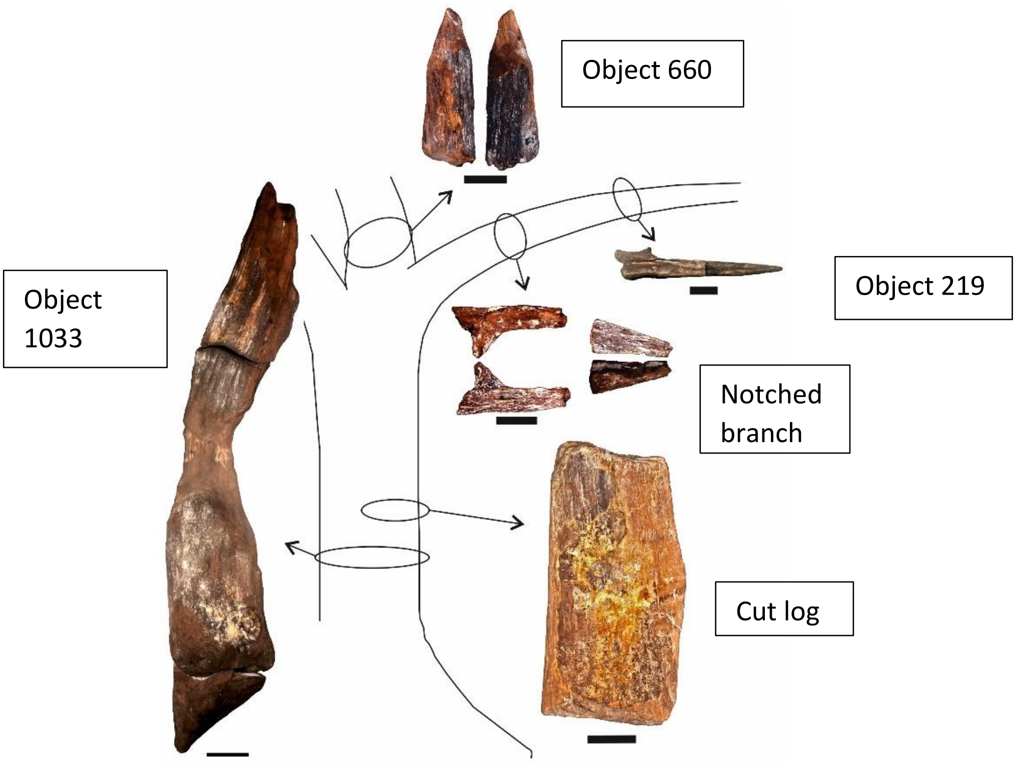

Figure 4.1. The anatomical location of the wood used at Kalambo Falls, site BLB. Object 1033 (BLB5, left) is derived from a tree trunk. The upper object (‘cut log’) in BLB4 (right, lower) is made on trunk wood. Object 660 from BLB3 (‘wedge’, top) is made from the upper part of a tree trunk or large branch. The lower object from BLB4 (‘notched branch’, right, centre) is made on a split branch. Object 219 from BLB2 (‘digging stick’, upper, right) is also branch wood. (Scales = 10 cm.)

5. Infrared (IR) spectroscopy investigation of waterlogged wood from Site BLB, Kalambo Falls, Zambia

In this study, we tried to detect areas of wood exposed to heating. Raman Spectroscopy was used as a preliminary investigation, but due to the small sample size, the wood flakes were obliterated by the laser before the analysis was completed. Given the limited quantity of wood available, we chose infrared spectroscopy which requires only a small sample.

Samples

Two pieces of waterlogged wood were analysed, BLB2 (object 219) and BLB5 (object 1033). Samples were collected using a Swann-Morton No. 3 scalpel and were approximately 5 mm x 5 mm x 2mm or less. On object 219, samples were taken from near the tip (A1, A2), mid-section (B1, B2) and from the broad end (C1, C2). On object 1033, samples D1 and D2 were taken from the broad exposed base of Part 3 (see Fig. 2a, 4; Extended Data Fig. 2a). Samples E1, E2 were taken from the upper part of the notch (Extended Data Fig. 1 a-c) which was considered a possible area for preserving traces of charring based on the hypothesis that partial charring would ease the process of cutting a large notch into the log (e.g. Aranguren et al. 2018). Samples F1, F2 were taken from the lower part of the notch (Extended Data Fig. 2b), as another possible area of burning.

All samples were freeze-dried before analysis to remove moisture. The spectra were recorded using flake samples larger than the ATR diamond crystal. Opus 7.2 software was used to process the spectra of each sample using 30 scans covering a wavelength range of 4000-500 cm-1.

Sample Name	Location on Wood	Observations
BLB2 (object 219)		
A1 and A2	Taken 7cm from the tip	
B1 and B2	28cm back from the tip.	
C1 and C2	17cm back from break next to radiocarbon dating sample.	
BLB5 (object 1033)		
D1 and D2	Taken from wide base where radiocarbon samples taken	
E1 and E2	Taken 48cm from base. From part one notch.	Possible area of burning
F1 and F2	Taken 38cm from ridge. Sample from ridge in the notch.	Possible area of burning

Table 5.1: Descriptions for each area, along with the notes recorded at the time of sampling.

As part of this study, Royal Botanical Gardens, Kew (RBG) supplied a modern comparison sample of *Combretum zeyheri* with IR spectra from three separate areas on the wood. The three samples came from the Economic Botany Collection of RBG Kew, catalogue number: 75630. There was no preparation of the samples before analysis. Perkin Elmer Frontier FT-IR/NIR spectrometer with UATR and NIR accessories was used to conduct the analysis. Four

scans were performed for each sample in the Mid-IR (4000-550 cm-1) and 32 in the Near-IR investigations (1000-4000 cm-1). There is a slight difference in the collection parameters between the two investigations, but the spectra can still be compared.

Results

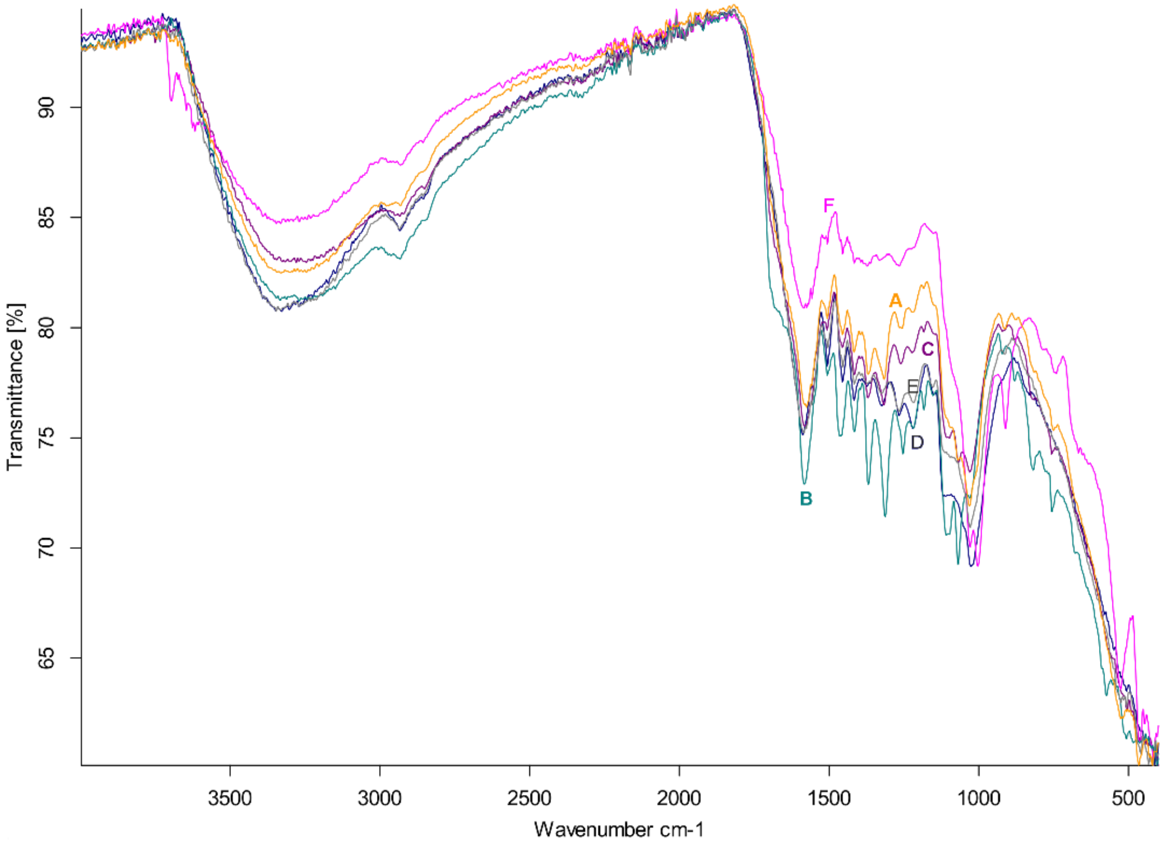

Figure 5.1. Spectra for BLB2 object 219 (‘digging stick’), Samples A-C, and BLB5 (object 1033) samples D-F.

Figure 5.1 shows the spectra from the locations sampled (see Table 5.1). There were no clear differences among the spectra from each location (for example, A1 and A2), and the results were collated.

IR spectra for wood samples vary significantly from location to location. The three spectra object 219 (BLB2 ‘digging stick’) and object 1033 (BLB5) differ significantly in transmittance, with sample F from BLB5 significantly different from the others. As a result, even within the same sample, the wood condition varies significantly, illustrating the difficulty of using IR to study archaeological wood.

Silica, calcium carbonate, and other minerals are known to fossilise and preserve ancient wood via mineralisation. Identifying typical peaks for silica in IR spectra could suggest the petrification of the wood through mineralisation. Pure silica exhibits Si-O stretching vibrations at 1100 cm-1, with a shoulder at 1200 cm-1. A second peak can be found between 800-470 cm-1 due to the Si-O ring structure (Karmakar, et.al, 2016:25). Spectra for sample A

(Figure 5.1) show this feature very clearly, with the peak and shoulder around 1185 cm-1. By contrast, silica peaks are absent in the modern samples of *C. zeyheri* (Figure 5.3). The high levels of silica in the archaeological samples indicates that mineralisation took place in a waterlogged environment.

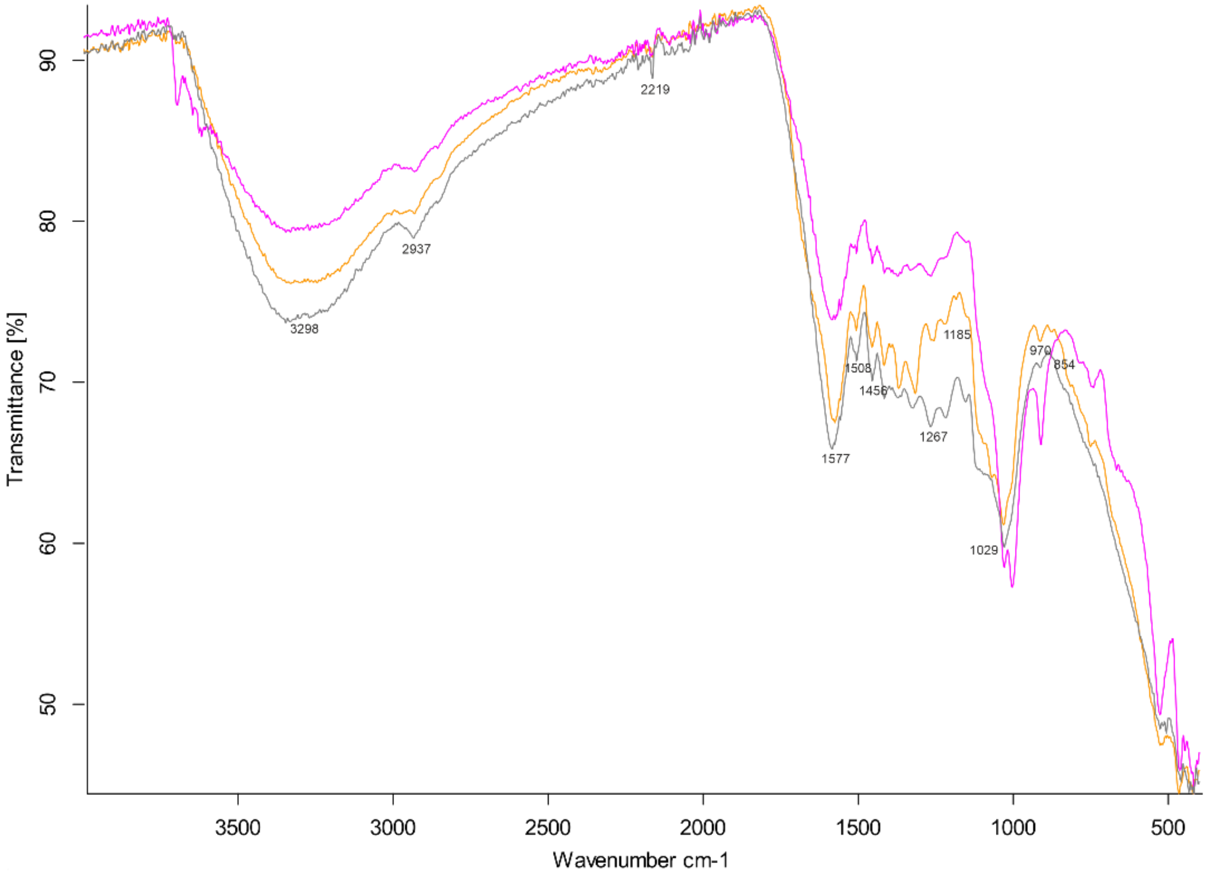

Figure 5.2. Comparison of the IR spectra wavelengths acquired for sample A (object 219) (yellow), E (object 1033) (grey) and F (object 1033) (purple).

In contrast to the other ancient samples, sample F has flattened silica peaks in its spectrum, which may indicate a potential burning zone (Figure 5.2). Leo and Barghoorn (1976:25) argue that carbonised wood is more resistant to silicification than unburnt wood because of the role of hydrogen bonds in the mineralisation process. In their experiment, silica precipitates from holocellulose and lignin because silicic acid is attracted to their hydroxyl groups. These organic components are depleted by burning or charring wood, making wood less susceptible to the uptake of silica. Changes in cell wall structure from burning also reduce porosity and permeability, preventing or slowing silica entry (Mitsi et al. 2021:6).

When examining the fingerprint region of the modern sample spectra, hemicellulose has a clear peak at 1732 cm-1 (Figure 5.3); this peak does not exist in the ancient specimens (Figure 5.2) and in fact the hemicellulose region is absent, suggesting that this organic matter has deteriorated.

The peaks for lignin are recognisable at 1507 cm-1 and for cellulose at 1050 cm-1 (Mitsi et. al. 2021:6). The peak region for lignin, generally between 1510 and 1269 cm-1, appears almost entirely flattened in sample F (Figure 5.2). This flattening indicates that the organic material has deteriorated, either through heating or mineralisation, resulting in a reduction in lignin content.

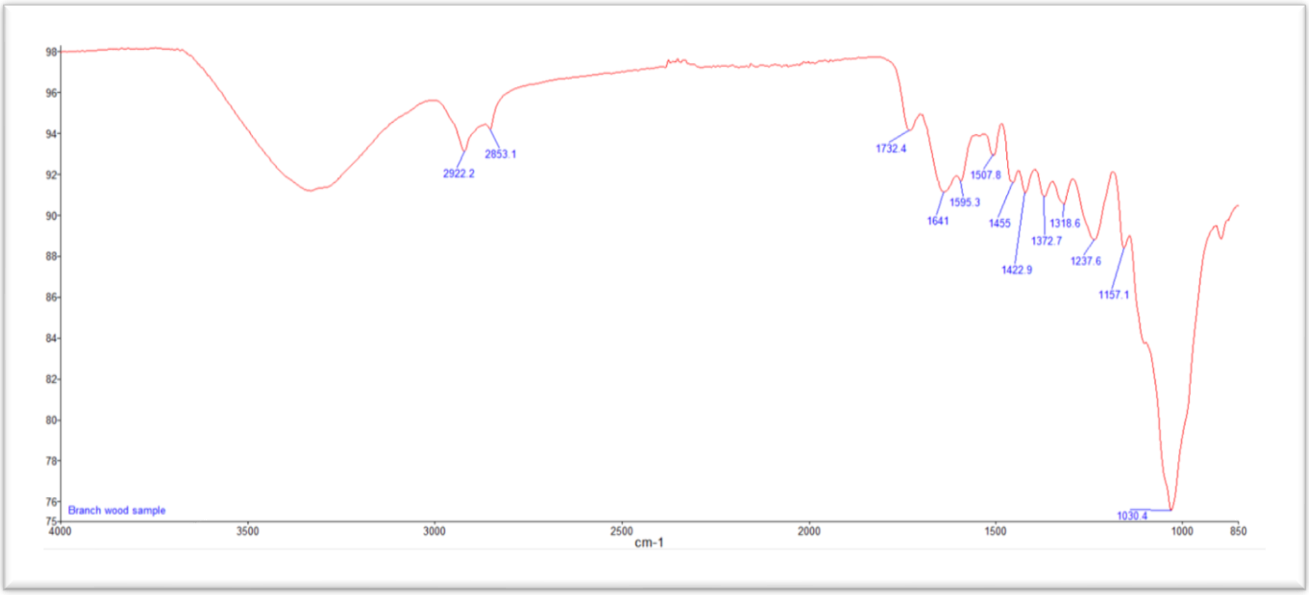

Figure 5.3. Mid-infrared spectrum of C. Zeyheri modern wood sample.

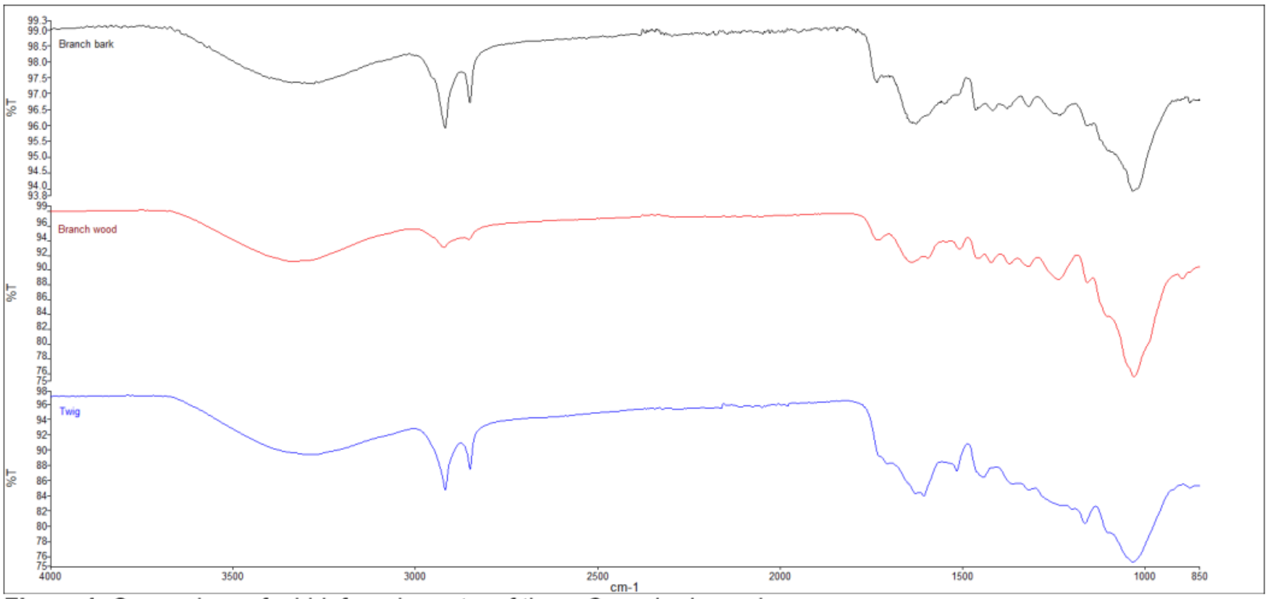

Figure 5.4. Mid-IR of modern reference samples showing little variability.

A comparison of the three modern samples of *C. zeyheri* revealed that the bands were remarkably similar, with only slight differences (Figure 5.4). This further demonstrates the

difficulty of investigating archaeological wood with IR due to unknown effects of geochemical processes on wood over time.

References

Aranguren, B. et al. Wooden tools and fire technology in the early Neanderthal site of Poggetti Vecchi (Italy). *Proc. Natl. Acad. of Sci*. USA **115**, 2054-2059 (2018)

Karmakar, B. Rademann, K. & Steanov, A. *Glass nanocomposites: synthesis, properties and applications*. Elsevier. Oxford. (2016)

Leo, R. F., & Barghoorn, E.S. Silicification of Wood. *Harvard University Botanical Museum Leaflets*, **25**, No. 1. 1-47. (1976)

Mitsi, E. Boyatzis, S. & Pornou, A. The Chemical Characterisation of Waterlogged Charred Wood: The Case of a Medieval Shipwreck. *Forests* **12**, 1594 (2021)

Mustoe. G.E. Wood Petrifaction: A New View of Permineralization and Replacement. *Geosciences* **7**, 119. (2017)

6. Taphonomic experiments on wood surface preservation

A two-part pilot study was undertaken to assess the impact of an active flowing river on the preservation of surface marks on wood, and to assess the preservation of surface features on saturated wood (see Methods; cf Dillehay 1997). The experiments showed that 1) abrasive flows result in the smoothing of facet edges and ridges, but tool marks remain visible; 2) saturated specimens lost definition of facet junctions from swelling and minor surface irregularities created during shaping were smoothed or disappeared; and 3) fluvial exposure did not create marks mimicking intentional shaping marks. Differences between the four dry control samples and the four saturated samples were only visible under SEM examination, with slight cracking observed on the samples allowed to dry. The stone tools produced thinner, more irregular facets than the steel knife, but the stone cut facets were more concave and the facet junctions clearer than cuts made with the knife.

The extent of smoothing observed in this pilot study suggests that running water can flatten irregularities and given enough time tool marks could be removed, as in the case of Site B at Kalambo Falls (Clark 2021). The following images (6.1 – 6.17) show the wood samples before and after exposure to the flume tank (unworked control sample, n=8; steel knife cut sample, n=8; bifacial flint tool modifications, n=8; unifacial flint tool modifications, n=8). Tables display the maximum and minimum sizes of facet marks for each group. All modified sticks were scraped unidirectionally down the long axis away from the experimenter for 10 - 15 minutes (see Methods).

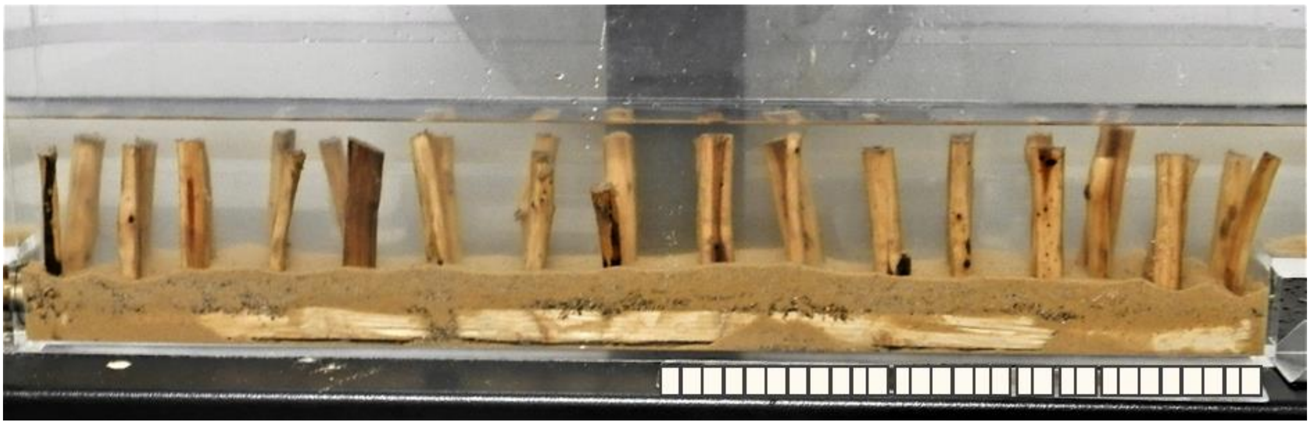

Figure 6.1. Photograph of wood samples in the flume inserted into a sand sediment base (Scale: 1cm per block).

Control sample (Figure 6.2) consisted of eight unmodified wood pieces of wood (S1-S8: left to right). Four were allowed to dry before analysis (S1-S4) (Figure 6.1) and four (S5-S-8) were analysed wet and kept submersed in fresh water throughout the two weeks of the study (Figure 6.2.). The surfaces are smooth with exception of knots (Figures 6.2, 6.3a).

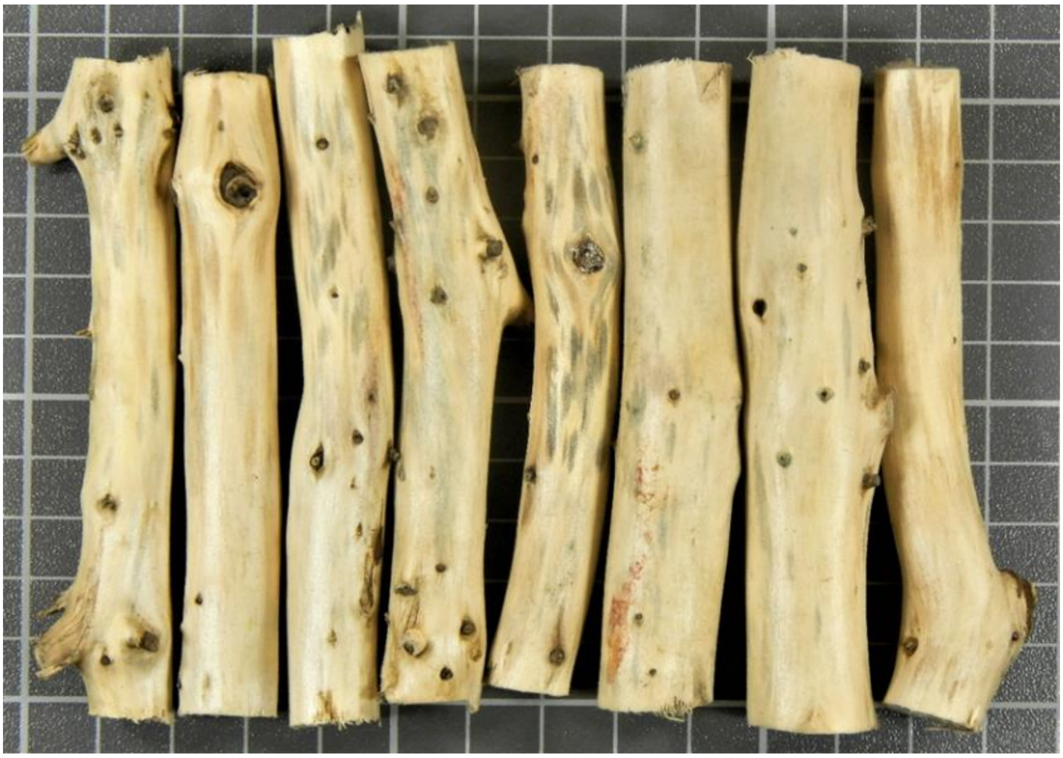

Figure 6.2. Eight control samples, S1-S8, left to right.

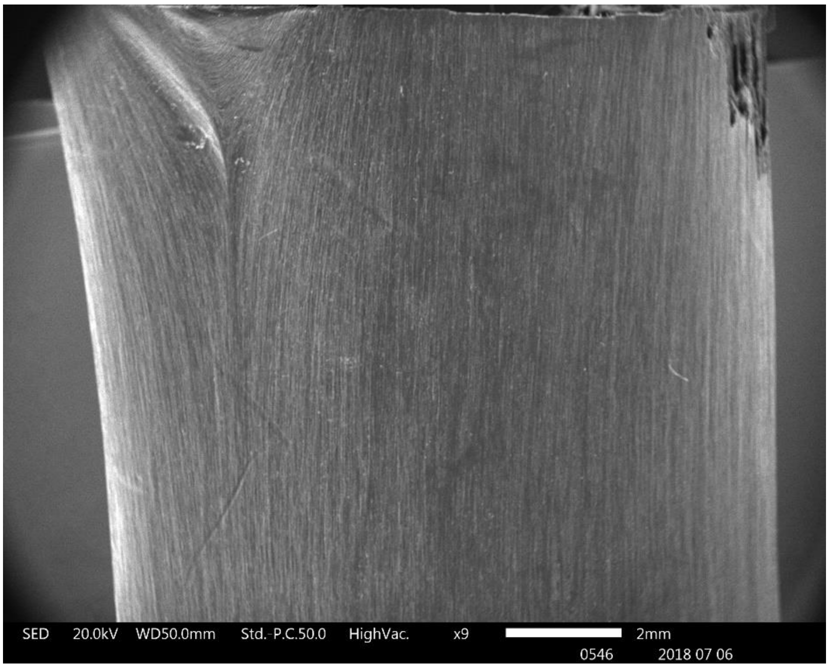

Figure 6.3a. SEM photomicrograph of S7 showing the natural lineation of the grain of the wood and small striations oblique to the grain which may have been produced in the process of removing the bark by hand on all 8 specimens used in each of the four experimental groups. The micrograph shown is representative of 8 independent analyses.

The steel knife cut samples (Figure 6.3b) have noticeably larger facets (Table 6.1) than wood shape using bifacial flint tool (Table 6.2) and unifacial flint tool (Table 6.3).

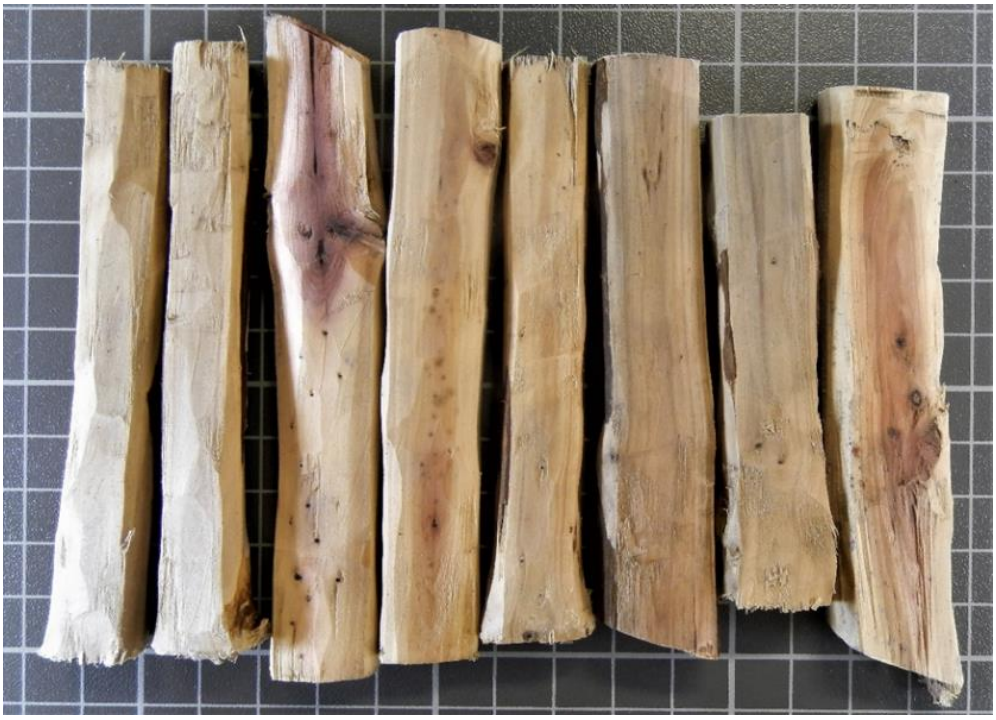

Figure 6.3b. Eight samples cut using a steel knife, K1-K8, left to right. K5-K8 were kept wet throughout the study and K1-K4 were allowed to dry before analysis.

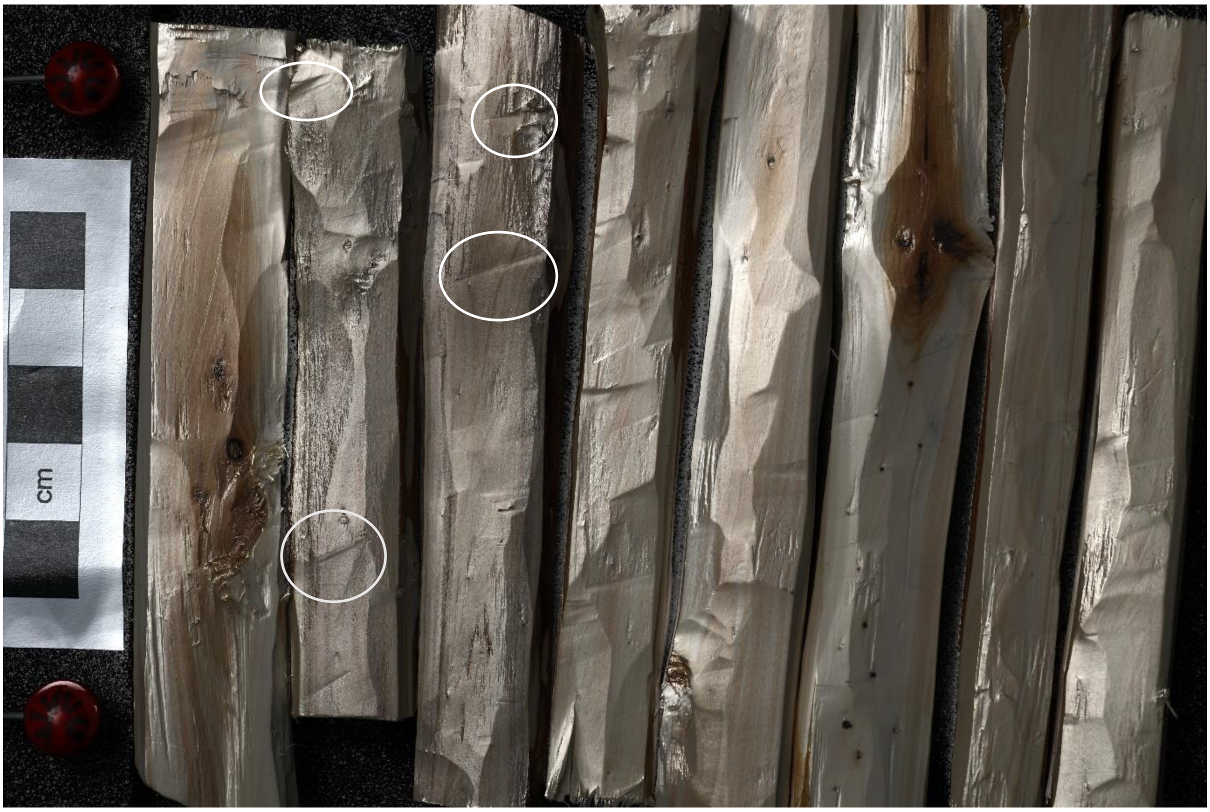

Fig. 6.3c Spectral RTI of steel cut sticks showing long, flat facets with abrupt terminations (circled). Ki – K8 right to left.

Specimen Number	Largest facet	Smallest facet
K1	8.93mm	3.53mm
K2	9.52mm	3.55mm
K3	10.18mm	4.38mm
K4	8.70mm	2.82mm
K5	8.06mm	4.24mm
K6	10.32mm	4.38mm
K7	7.90mm	2.85mm
K8	11.84mm	4.10mm

Table 6.1. The largest and smallest facets identified on each specimen worked with a knife.

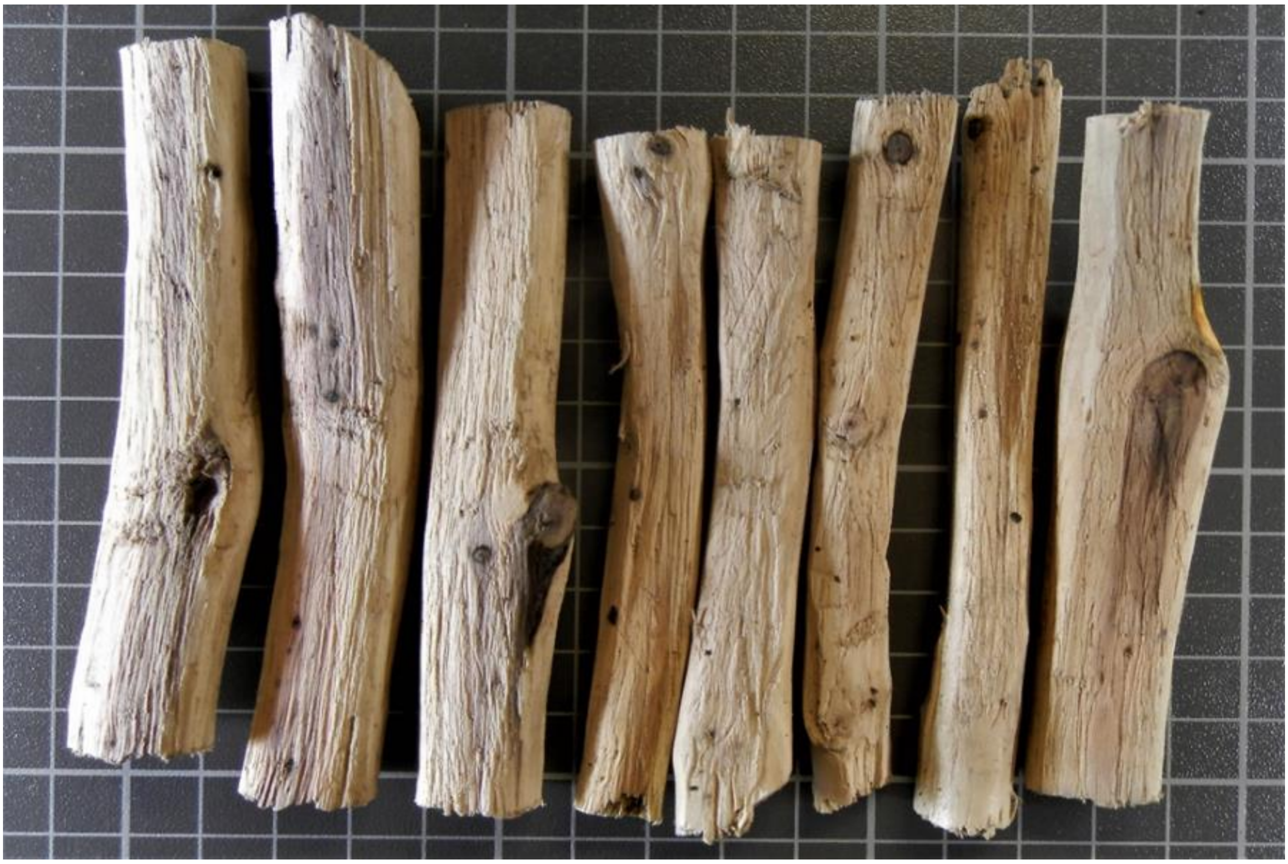

Figure 6.4. Eight samples shaped with a bifacial flint tool, B1-B8, left to right. B1-B4 were allowed to dry before analysis and B5-B8 were kept wet throughout the study.

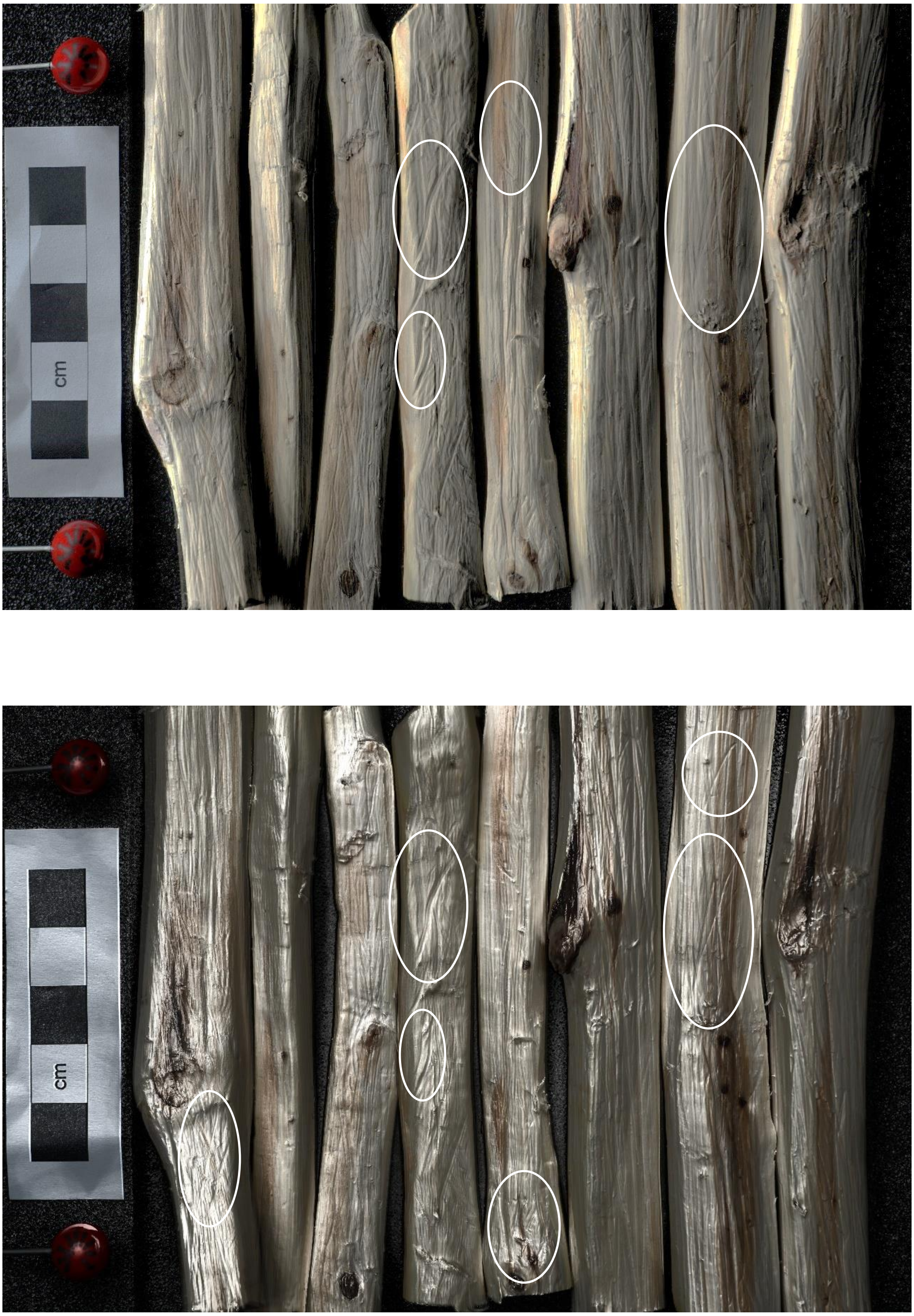

a

b

Figure 6.5 RTI images (a=normal view, b=spectral view) of sticks B1-B8 (left to right) scraped with bifacial flint tools showing the rough surface and varying direction of striations, and (white circles) curving facets (B4, B7) (white circles and see Figure 6.6).

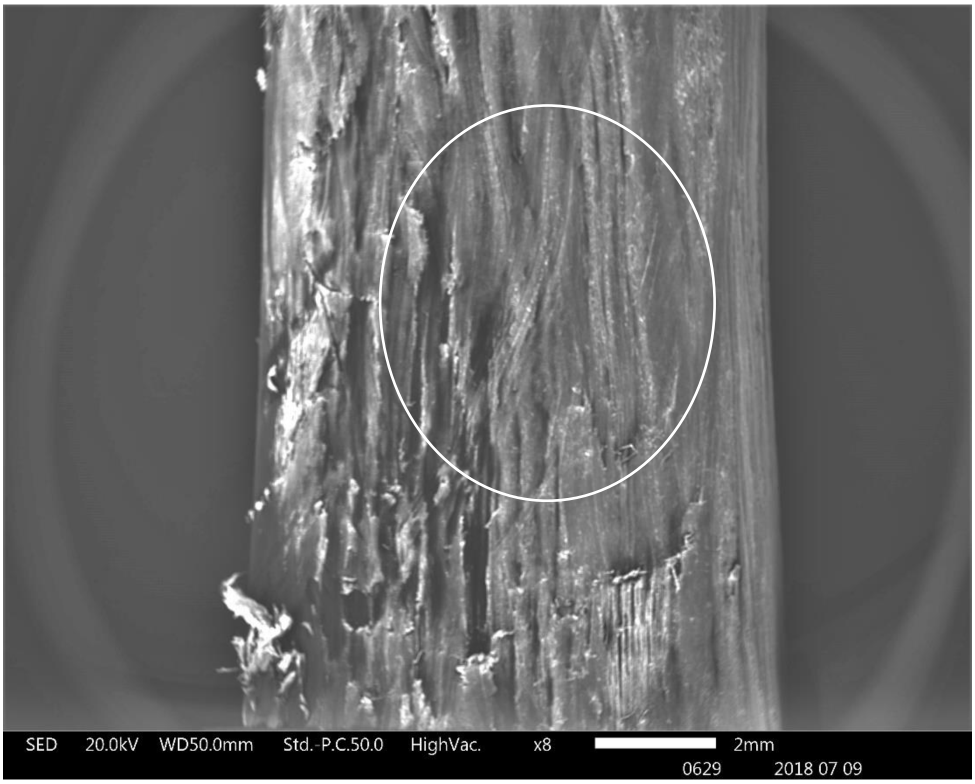

Figure 6.6 SEM photomicrograph of B2 showing the roughness of microfacets and striations that start to curve off to the side (circled). The micrograph is representative of observations from 8 separate replicates.

Four independent experiments were undertaken comparing pairs of saturated versus non-saturated specimens.

Specimen Number	Largest facet	Smallest facet
B1	0.9mm	0.21mm
B2	0.48mm	0.23mm
B3	0.69mm	0.29mm
B4	0.75mm	0.16mm
B5	1.3mm	0.42mm
B6	0.71mm	0.14mm
B7	0.52mm	0.17mm
B8	1.4mm	0.20mm

Table 6.2. Largest and smallest facet marks created using a bifacial flint tool on specimens B1-B8.

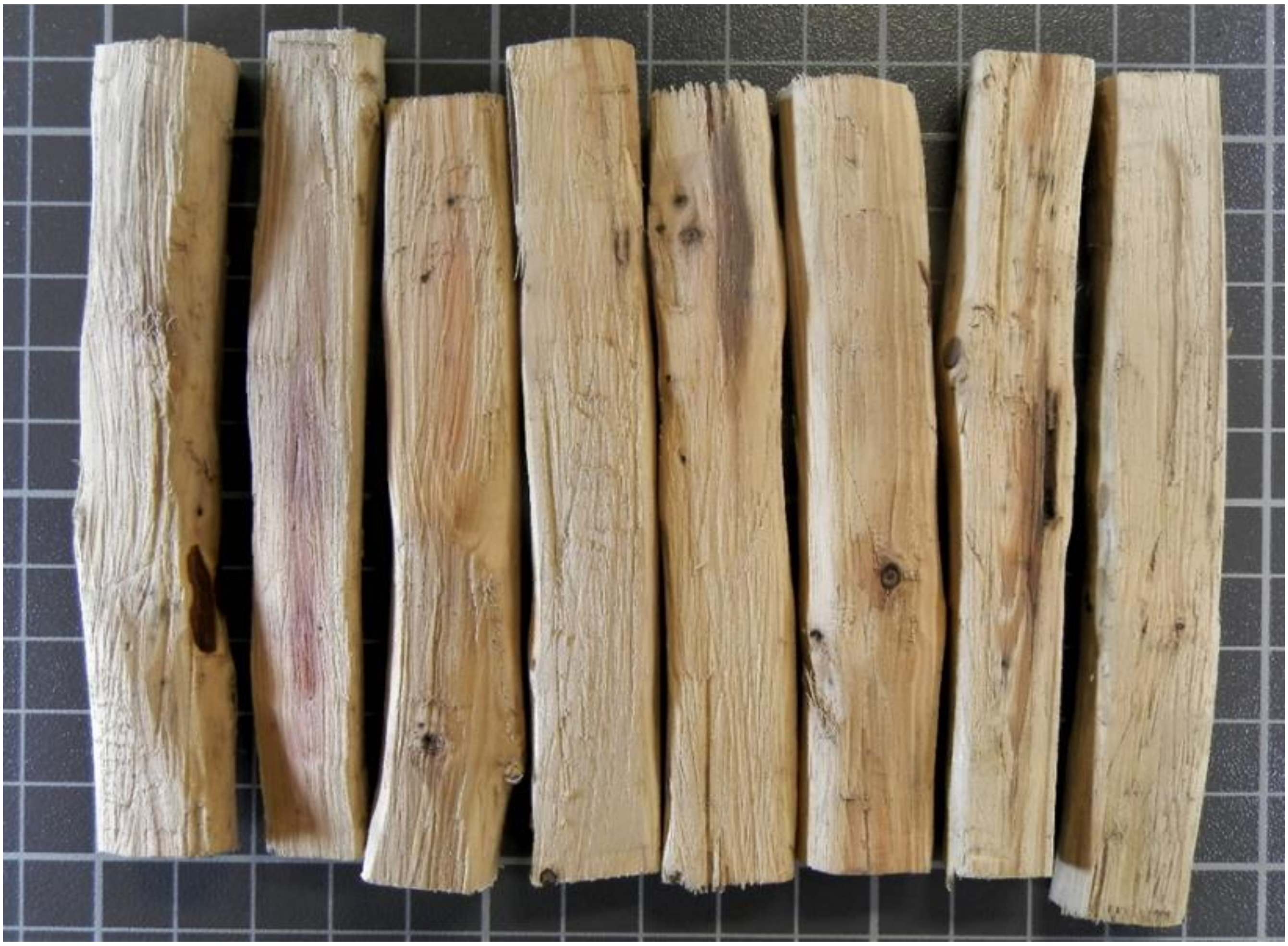

Figure 6.7. Eight samples shaped with a unifacial flint tool, U1-U8 (left to right). Samples U1-U4 were allowed to dry before analysis and U5-U8 were kept wet throughout the study.

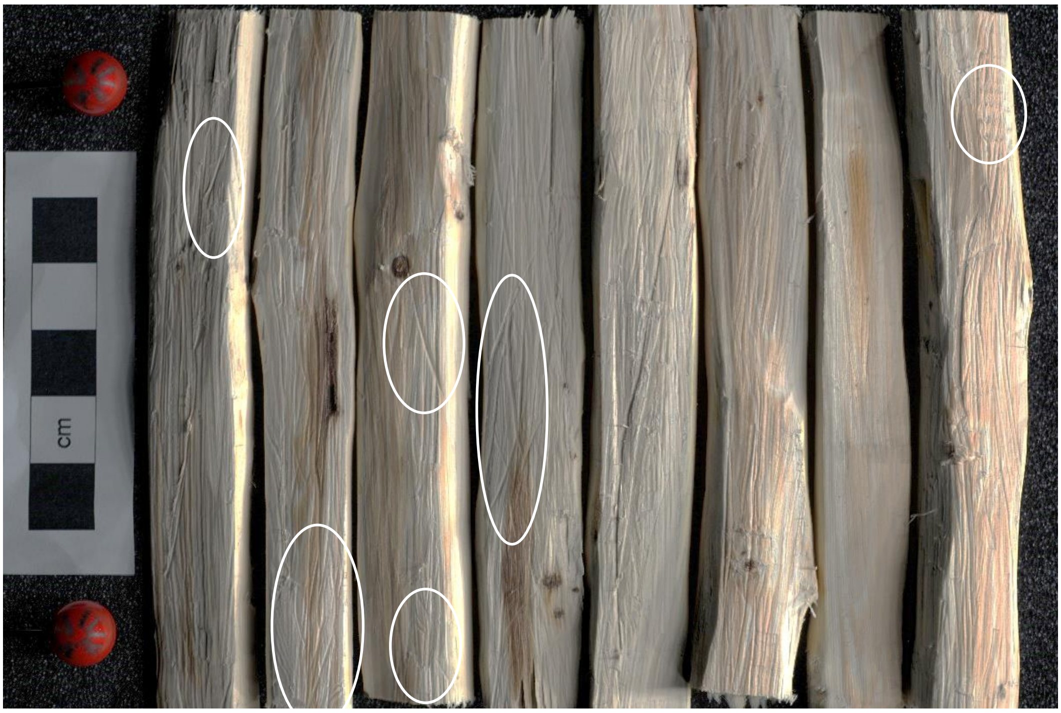

a

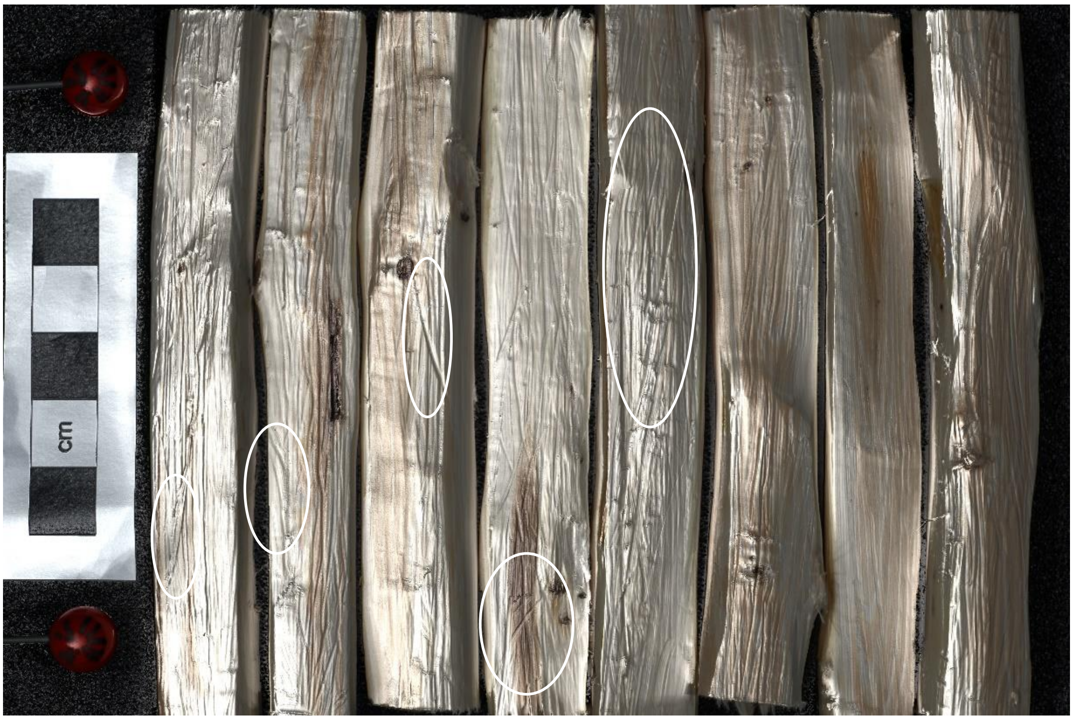

b

Figure 6.8 RTI images (a=normal view; b=spectral view) of sticks scraped using a unifacial flint tool (U1-U8, left to right). The images show the rough texture of the surface and the varying orientation of the striations (white circles) as well as jams and imperfections.

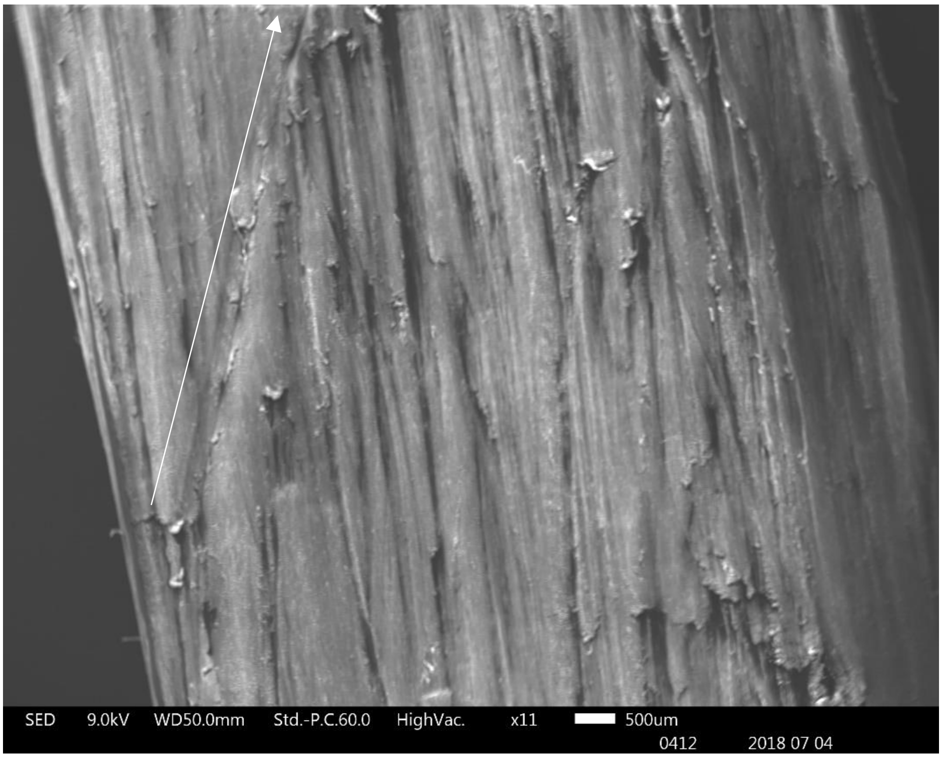

Figure 6.9 SEM photomicrograph of specimen U5 showing the roughness of the worked surface and striation oblique to the grain (arrow). The surface features are indicative of observations made during 8 individual experiments.

Specimen Number	Largest facet	Smallest facet
U1	0.94mm	0.11mm
U2	0.36mm	0.06mm
U3	0.82mm	0.05mm
U4	0.79mm	0.08mm
U5	0.99mm	0.12mm
U6	0.79mm	0.14mm
U7	1.1mm	0.16mm
U8	0.99mm	0.13mm

Table 6.3. Largest and smallest facets created by a unifacial flint tool, U1-U8.

The following RTI images show the surface features by group, recorded at the end of the study. Note, that the numbering of the individual specimens is shown right to left.

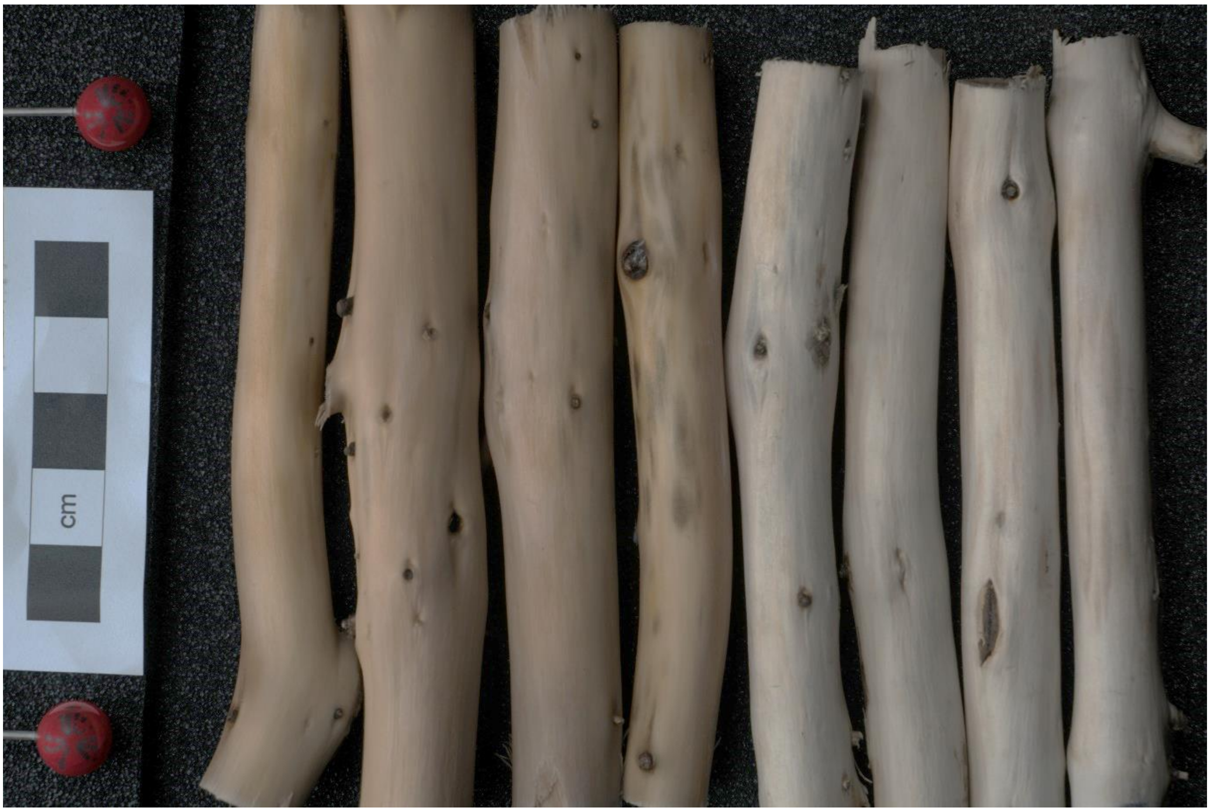

Figure 6.10 RTI image in normal viewing mode of the control sample (S1-S8, right to left) showing the smoothness of these specimens and the imperfections (knots) still visible.

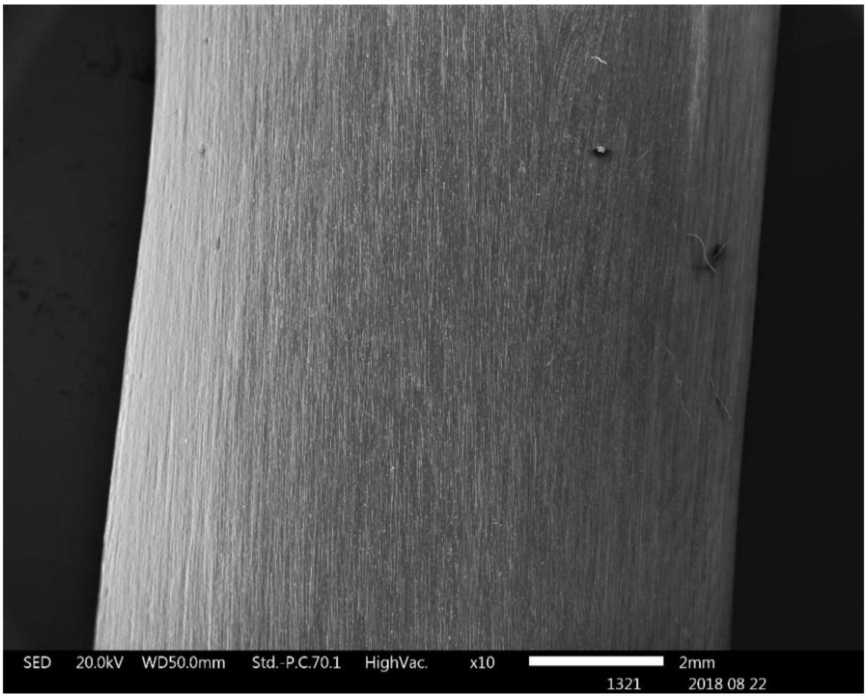

Figure 6.11 SEM photomicrograph of control specimen S8 showing the smooth linear surface grain of this specimen which was kept wet throughout the study. Micrograph shown is representative of results observed on 8 independent replicates.

.

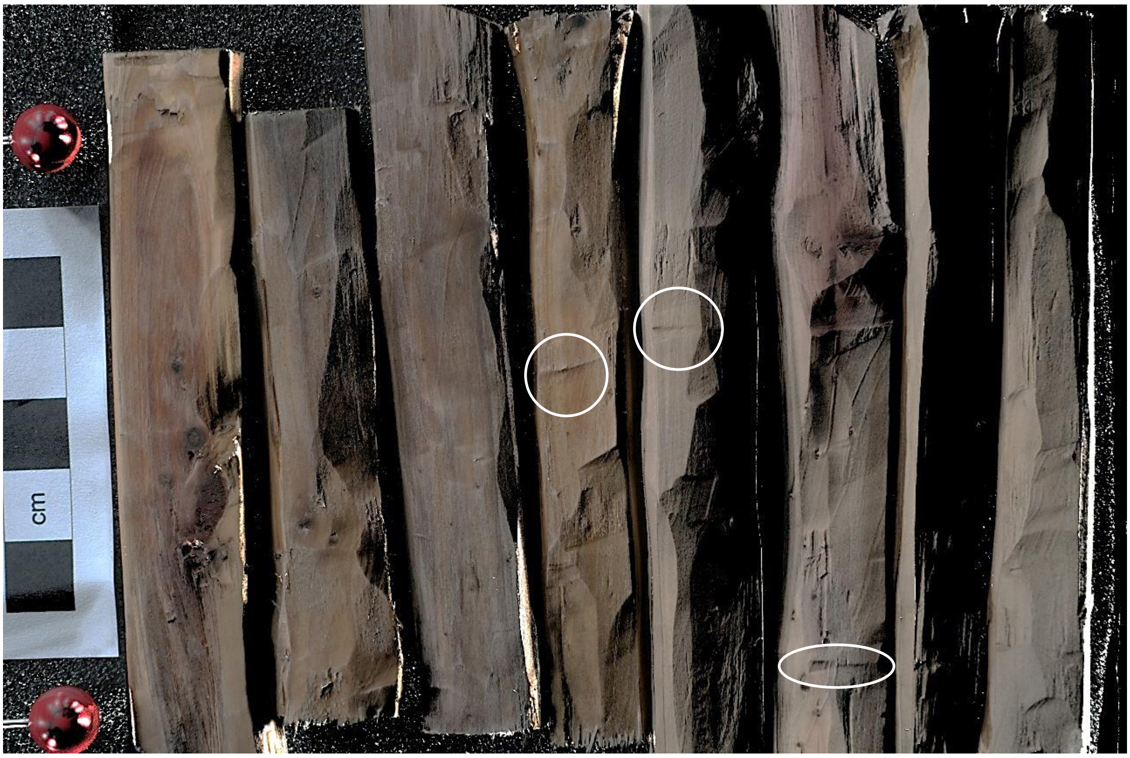

a

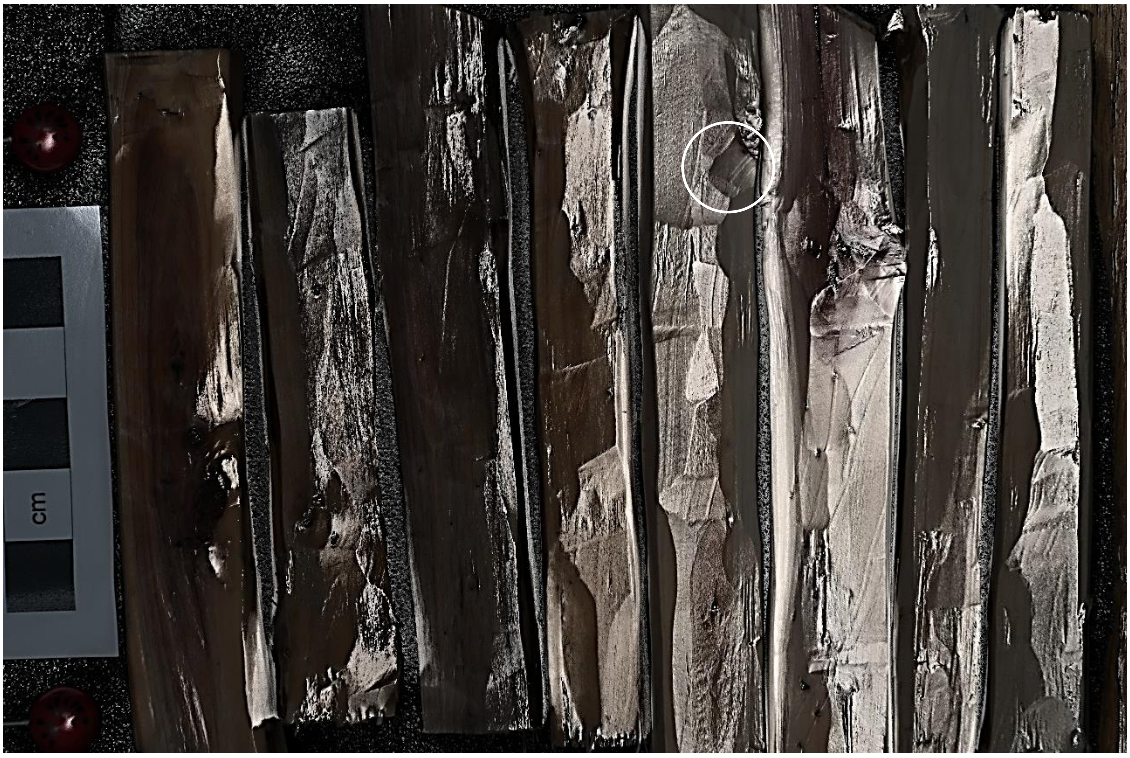

b

Figure 6.12 RTI images (a=diffuse grain view; b=spectral view) showing the smoothness of the facets and the presence of facet junctions on dry (K1-K4) and wet specimens (K5-K8) (white circles). Facets are less well-defined on wet specimens with marks on K8 losing the most definition. K1-K8, right to left.

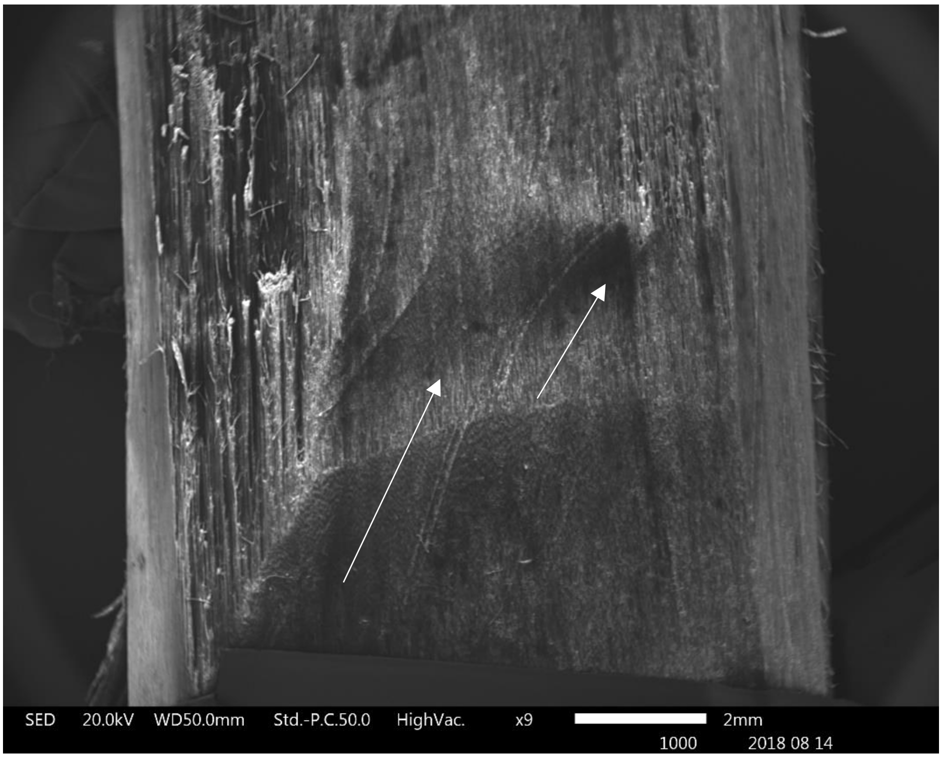

Figure 6.13 SEM photomicrograph showing slight tool marks and striations against the grain (arrows) visible on the dry specimen K1. Results shown are an example of similar observations across 8 separate replications.

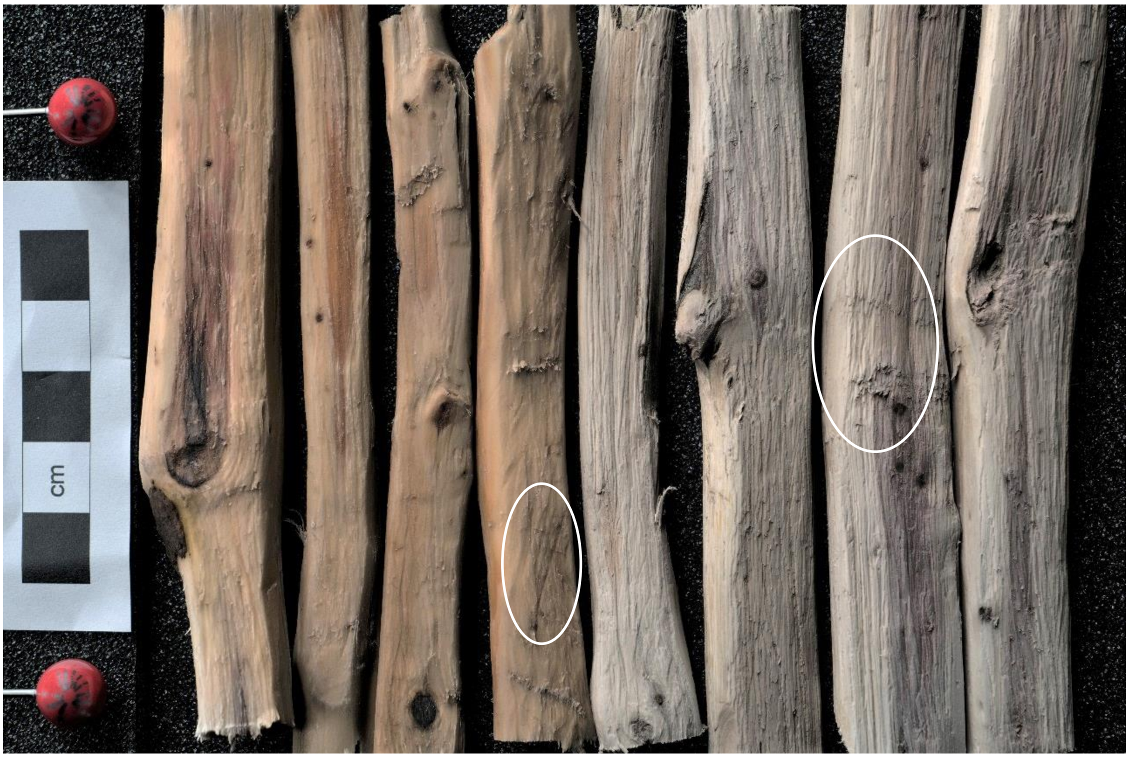

a

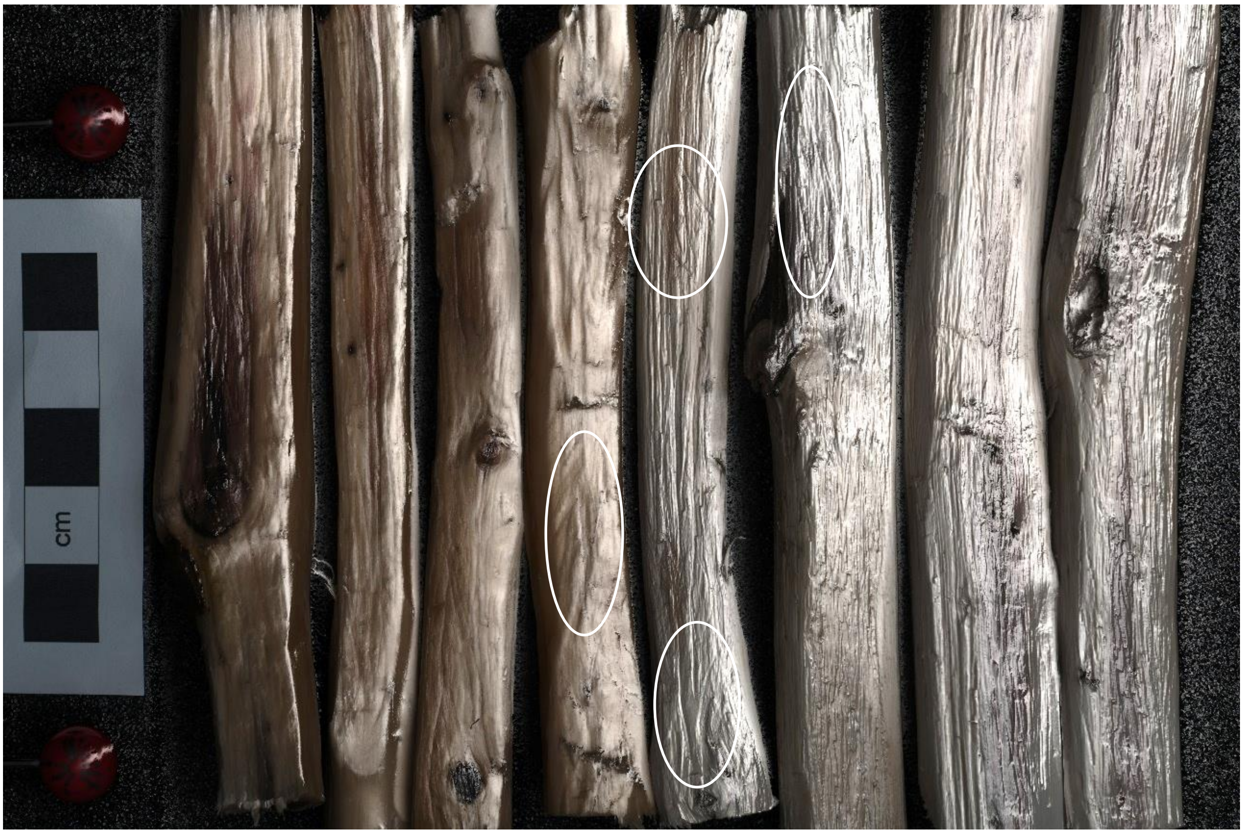

b

Figure 6.14 RTI images of bifacial tool shaping marks in normal (a) and spectral (b) viewing modes showing the smoothness of the facets on the wet specimens (B5-B8) compared with the dry specimens (B1-B4), but still maintaining facet junctions. White circles highlight a few striations that are still visible. B1-B8, right to left.

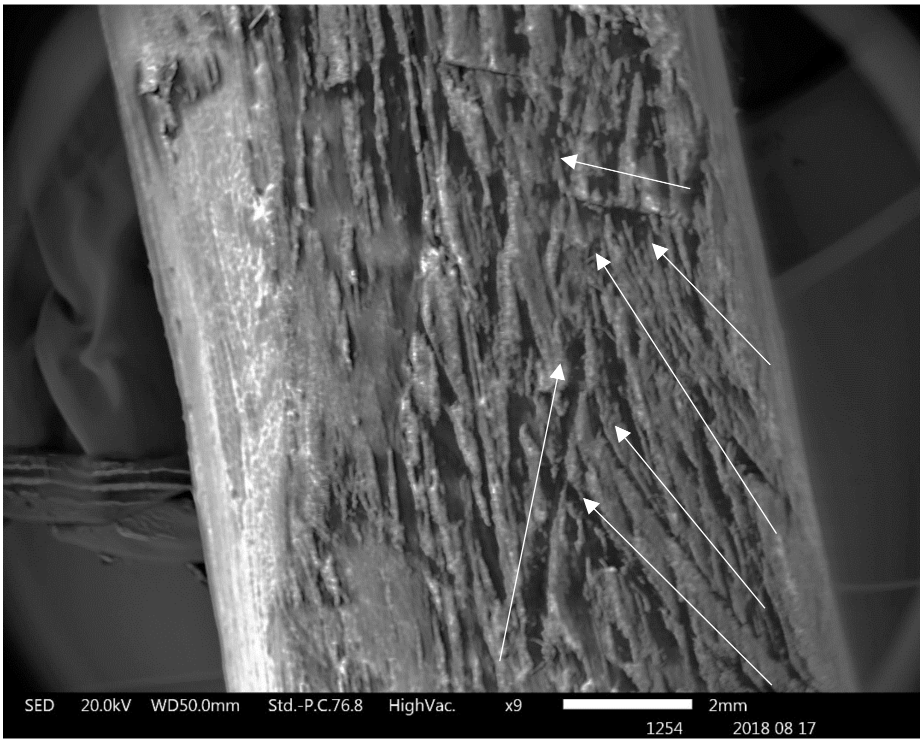

Figure 6.15 SEM photomicrograph of wet specimen B6 showing striations cutting across the grain. The micrograph is representative of results observed on 8 separate replicates.

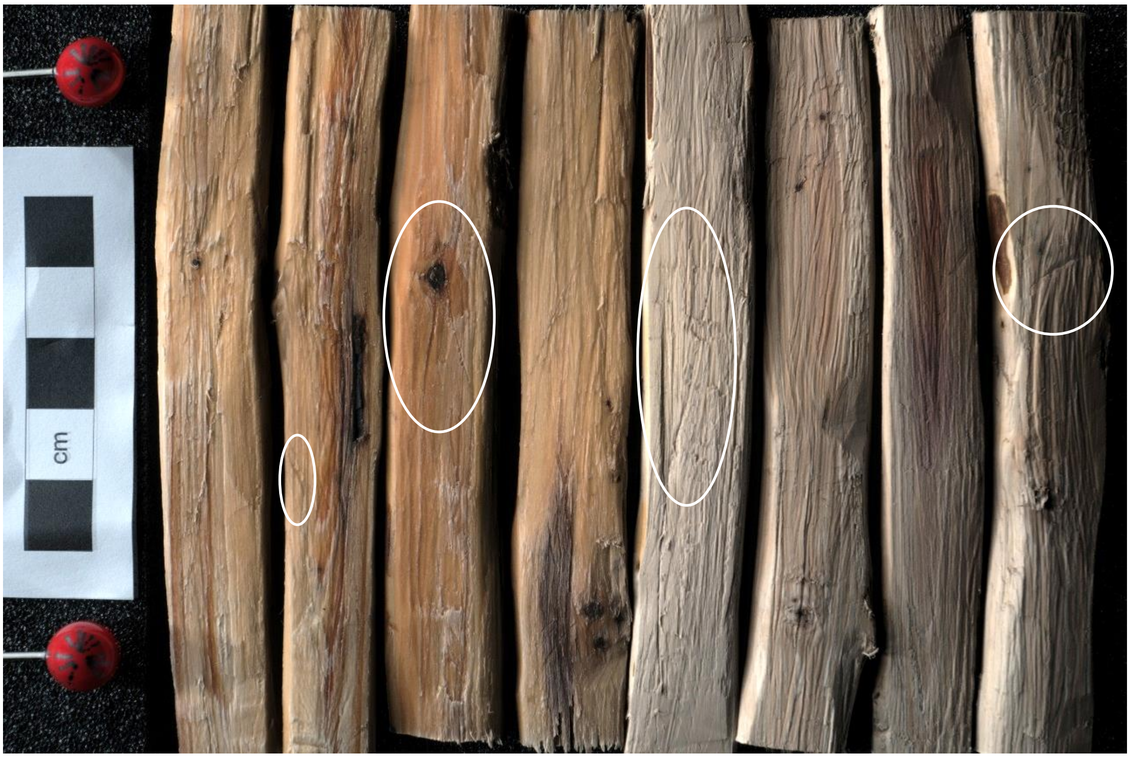

a

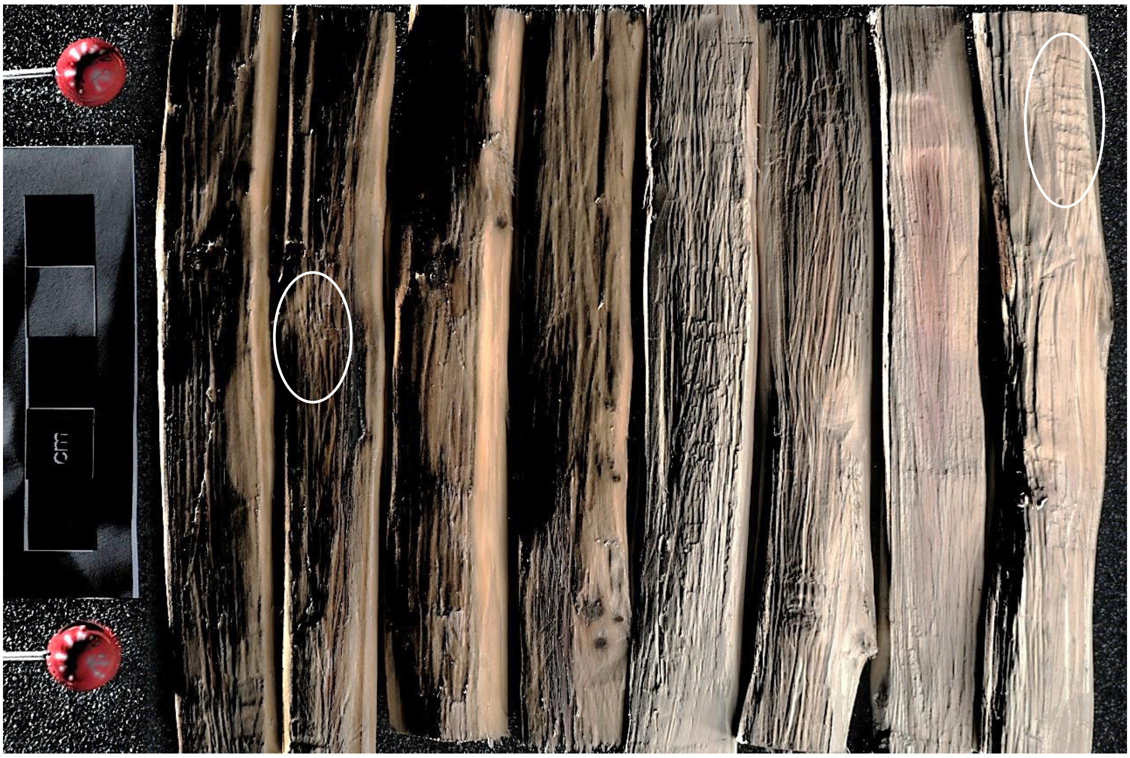

b

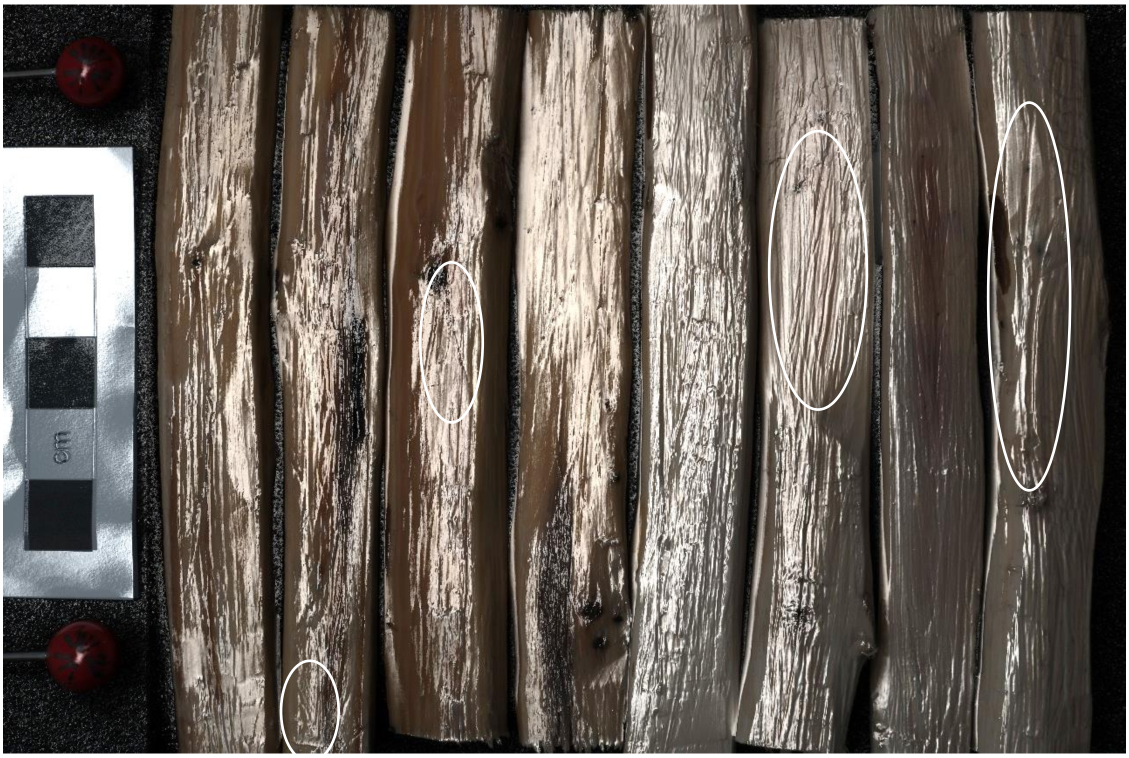

c

Figure 6.16 RTI images of unifacial tool shaped specimens in normal (a), diffuse grain (b) and spectral (c) viewing modes, showing the greater smoothness of the facets on the wet specimens (U5-U8) compared with the dry specimens (U1-U4) while both maintain facet junctions and striations. White circles highlight striations that are still visible. U1-U8, right to left.

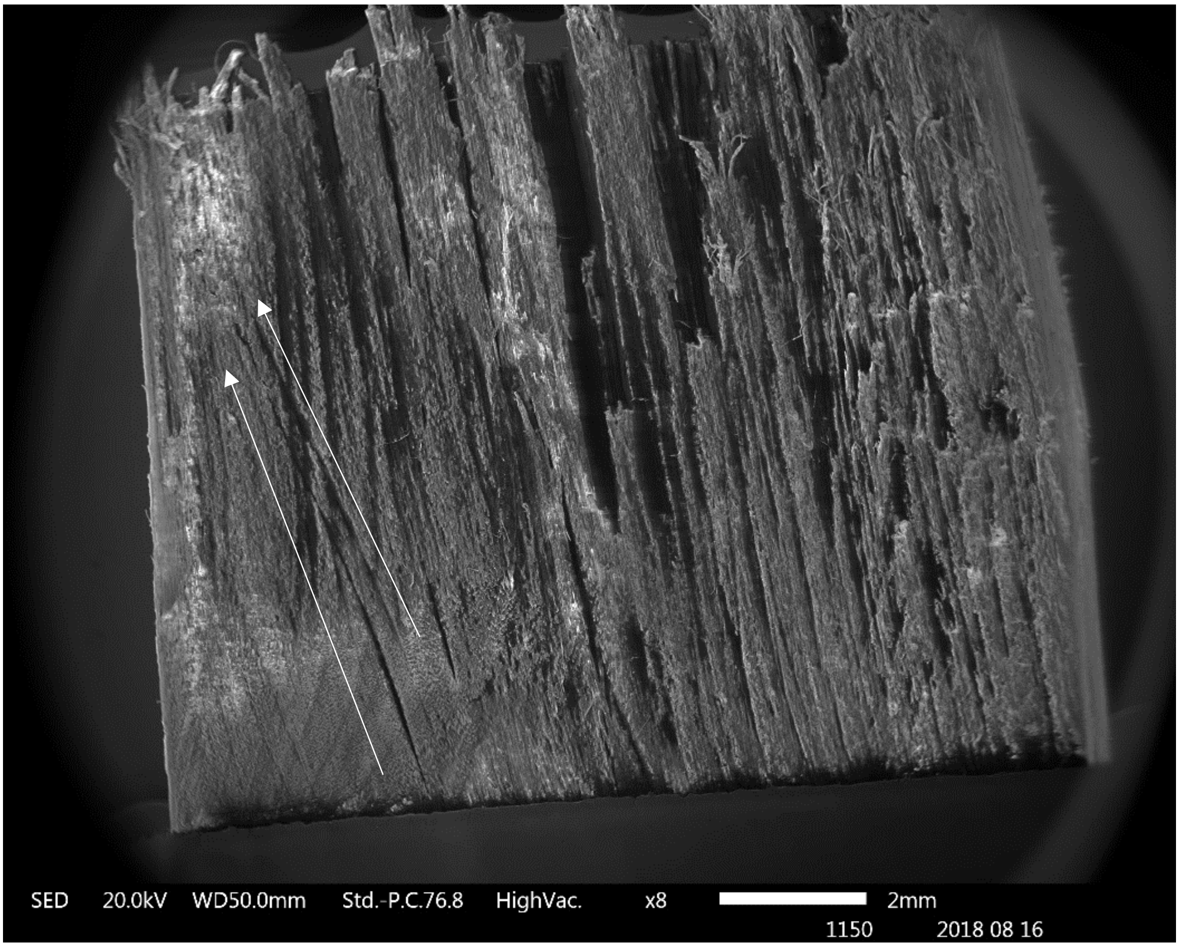

Figure 6.17 SEM photomicrograph showing striations cutting across the grain (arrows) visible on the wet specimen U5. Representative micrograph of observations made on 8 independent replicates.

References

Clark, J.D. (Ed.) *Kalambo Falls Prehistoric Site, Vol. III*. Cambridge: Cambridge University Press. (2001)

Dillehay, T.D. Archaeological and Taphonomical Analyses of the Wood Assemblage, in: Monte Verde: *A Late Pleistocene Settlement in Chile Volume 2. The Archaeological Context and Interpretation.* Smithsonian Institution Press, Washington, pp. 119–172. (1997)

7. Wood working experiments

Four experiments were undertaken to replicate marks observed on the modified wood pieces from site BLB, Kalambo Falls (see Methods for detail). **Experiment 1** assessed the marks left by scraping green elm and ash logs using quartzite flakes and scrapers, handheld and hafted (Figures 7.1 – 7.4). In all cases a series of tightly clustered grooves/striations result covering the entire area worked. This pilot study suggests hafted use appears to provide greater manual control, observed by the reduced curved striations caused when the hand slips during use. **Experiment 2** involved the making and use of wedge-shaped hazel branches to split birch. Compression damage perpendicular to the grain of the wood was observed on the tips (Figure 7.5). A follow-on experiment involved charring and scraping the wedges with flint flakes. The results (Figure 7.6) resemble the asymmetry and surface texture of the obverse side of the BLB3 'wedge' (object 660) tip (Extended Data Figure 6). **Experiment 3** used a bifacial flint tool ('handaxe') as a wedge to split a birch log with the base of the biface struck with a wood baton. The resulting splitting (Figures 7.7 – 7.9) resembles the deep basal split and side splits seen on the BLB3 'wedge' (object 660) (Extended Data Figure 6). **Experiment 4** replicated the chop marks on end 'a' of the BLB4 'cut log' (Extended Data Figure 8) using a hafted quartzite cleaver (socket haft in elm handle) to chop fresh elm. The tool was effective, and sharp stepped chops marks emerged during chopping resembling those on the 'cut log' (BLB4) (Figures 7.10 – 7.12) (see Methods for details.)

Experiment 1

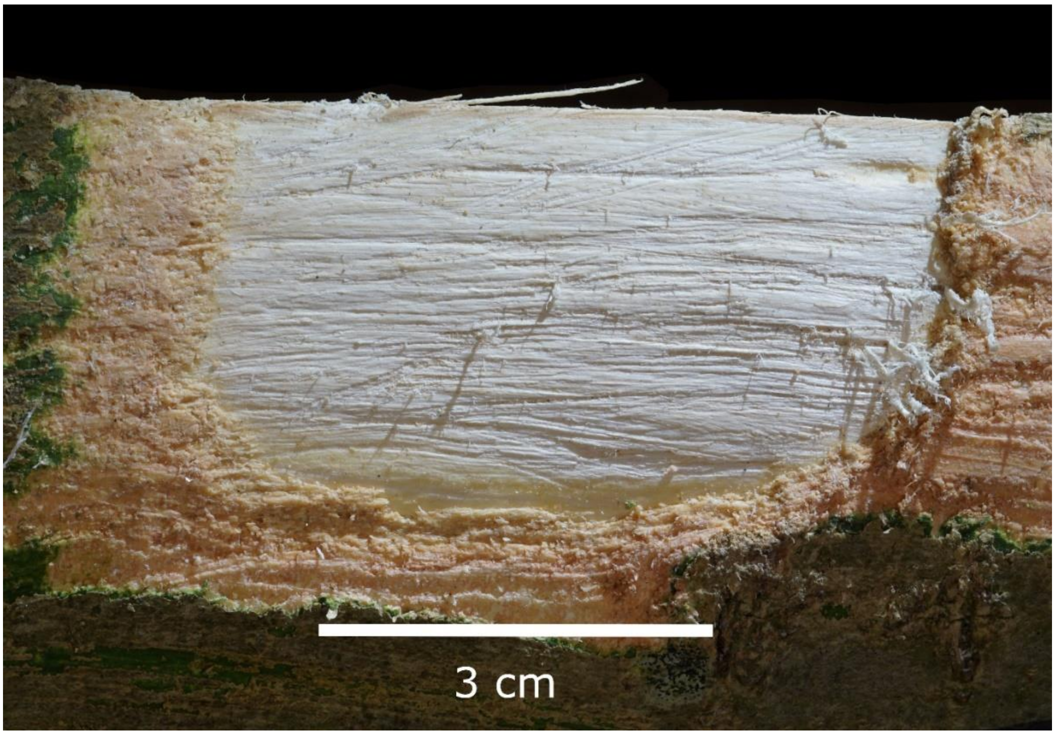

Figure 7.1. Area of ash log scraped using an unretouched quartzite flake.

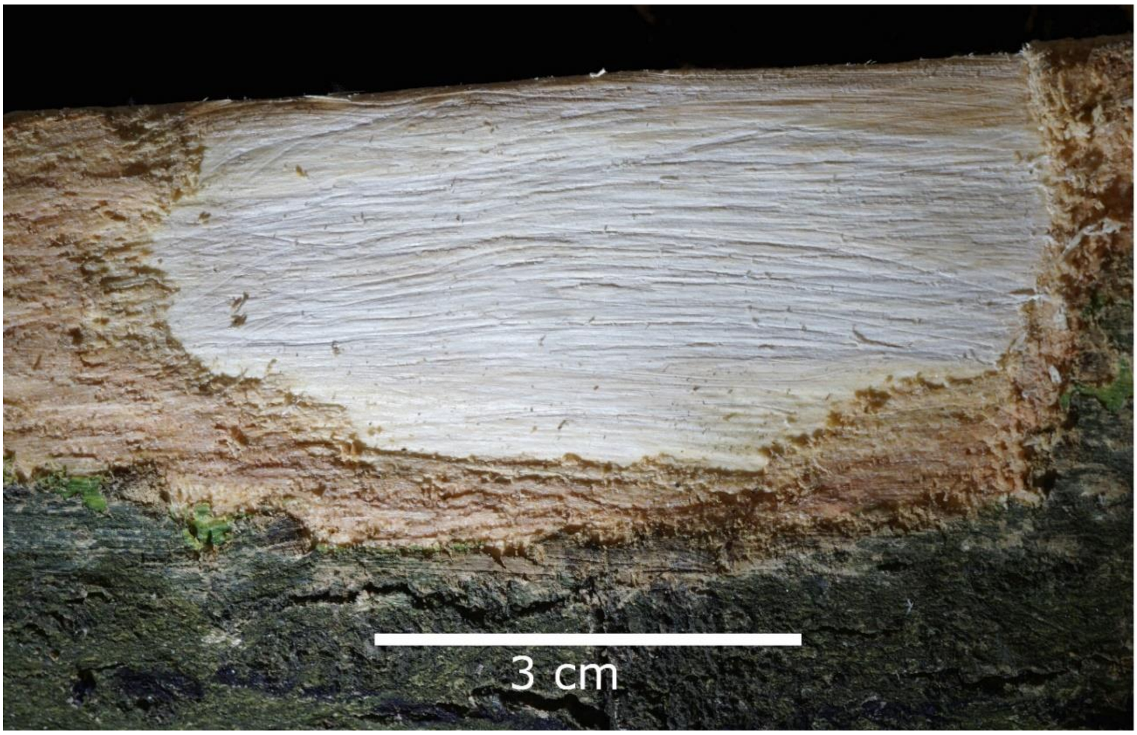

Figure 7.2. Area of ash log scraped with a retouched quartzite flake.

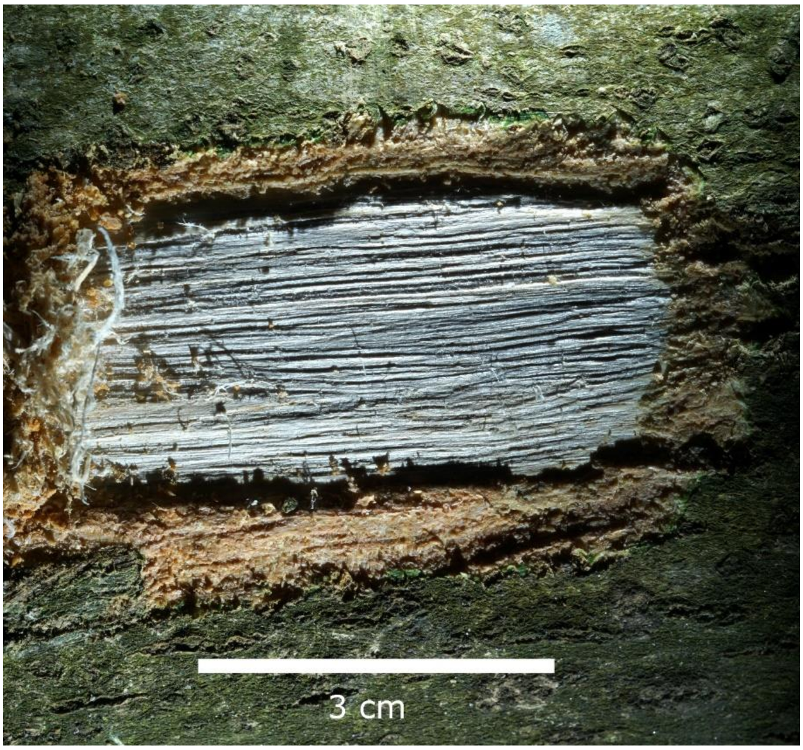

Figure 7.3. Area of ash scraped using a hafted scraper.

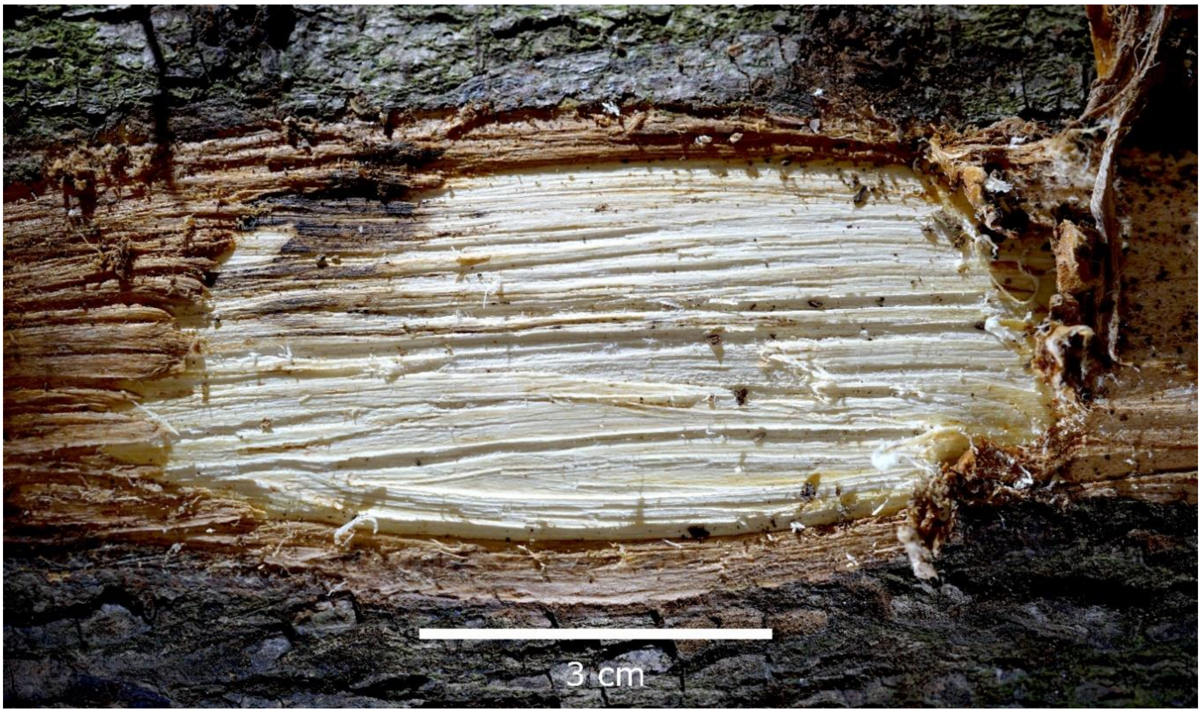

Figure 7.4. Area of elm scraped using a hafted scraper.

Experiment 2

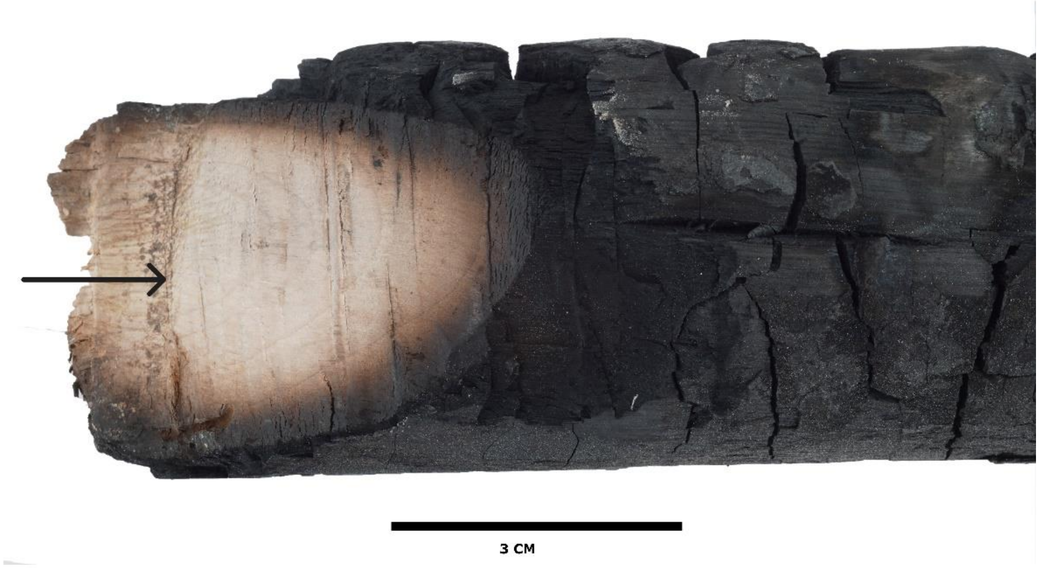

Figure 7.5. Experimental hazel wedge showing compression damage and blunting of the tip. The wood was charred before shaping.

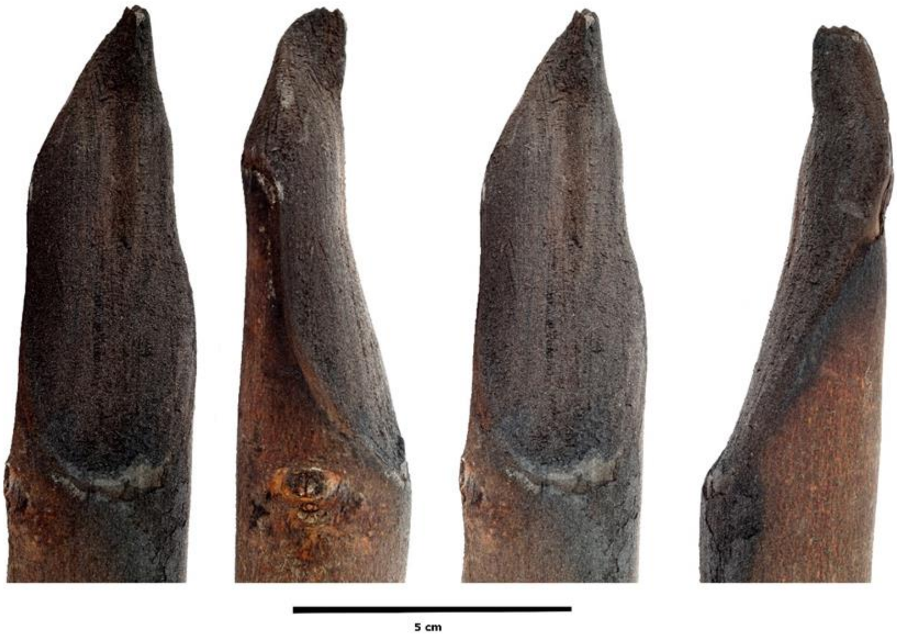

Figure 7.6. Charred and scraped hazel wedge tips, unused. (Scale = 5 cm.)

Experiment 3

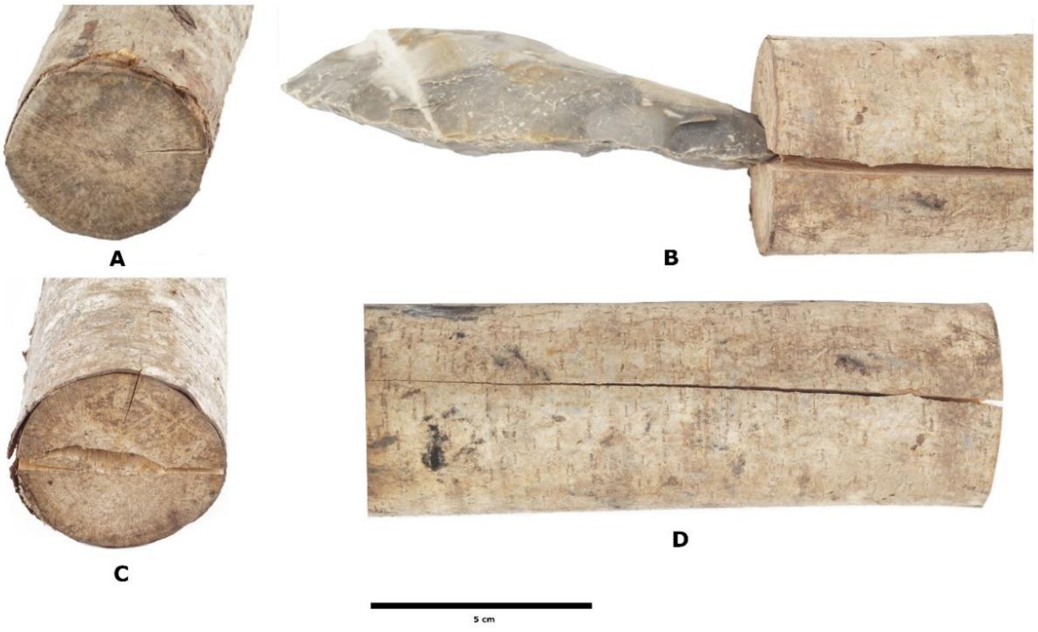

Figure 7.7 Hazel log splitting experiment using a biface wedge: A, before splitting; B, flint biface used as a wedge; C, D, basal and side splits resulting from the insertion of the biface wedge. (Scale = 5 cm.)

Experiment 4

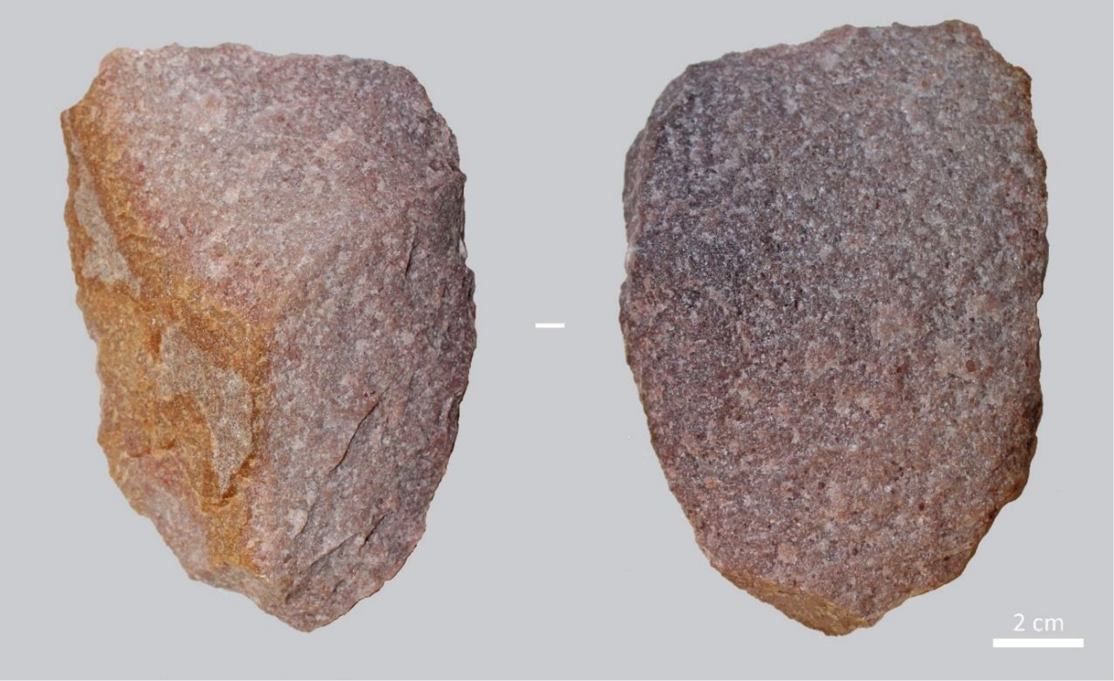

Figure 7.8. Quartzite cleaver before use.

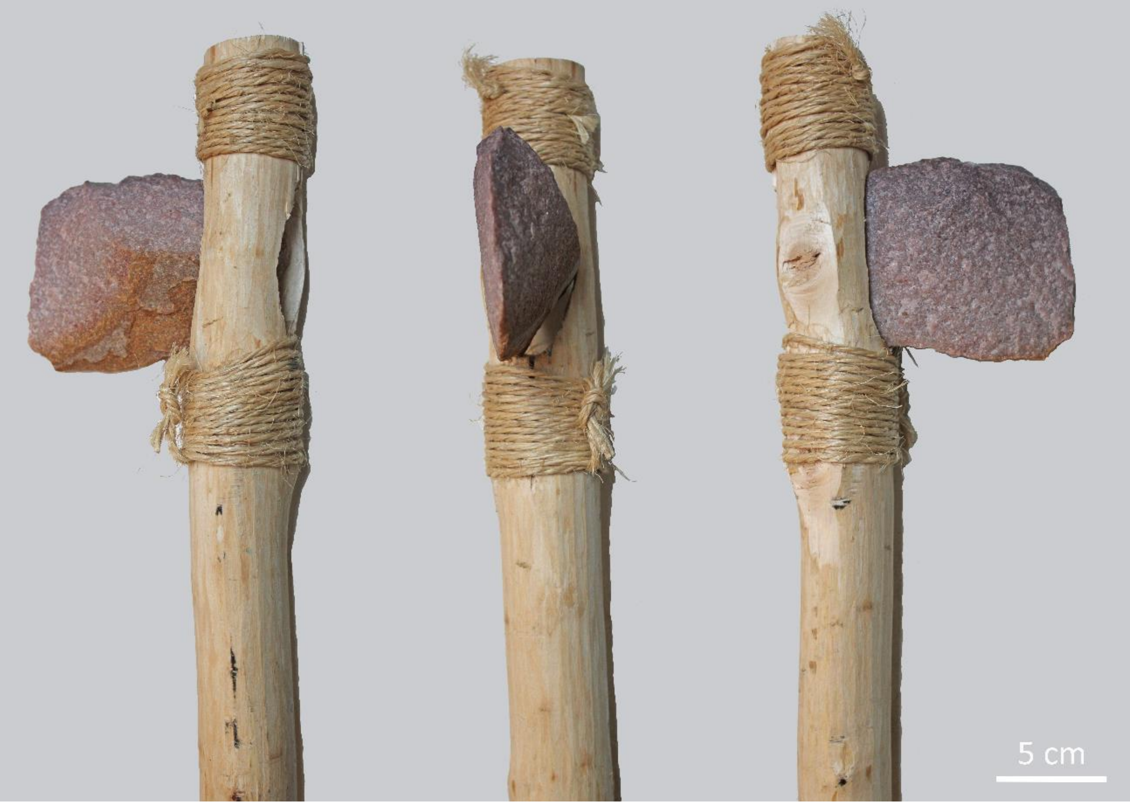

Figure 7.9. Experimental cleaver hafted in socketed handle out of elm wood. Split secured with vegetal bindings.

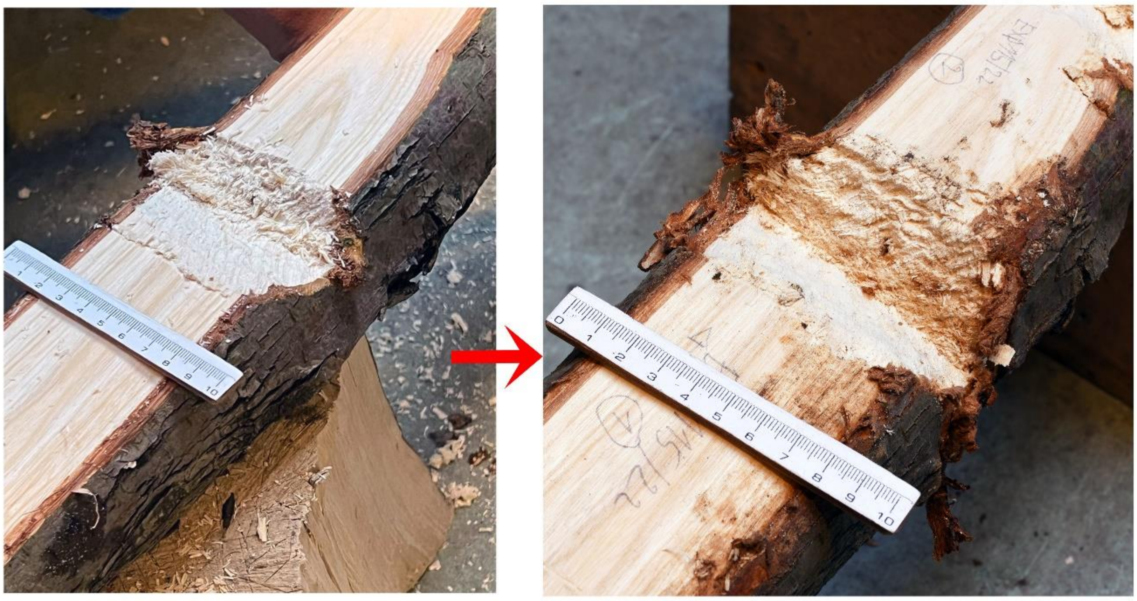

Figure 7.10. From left to right: gradual progression of cut during chopping.

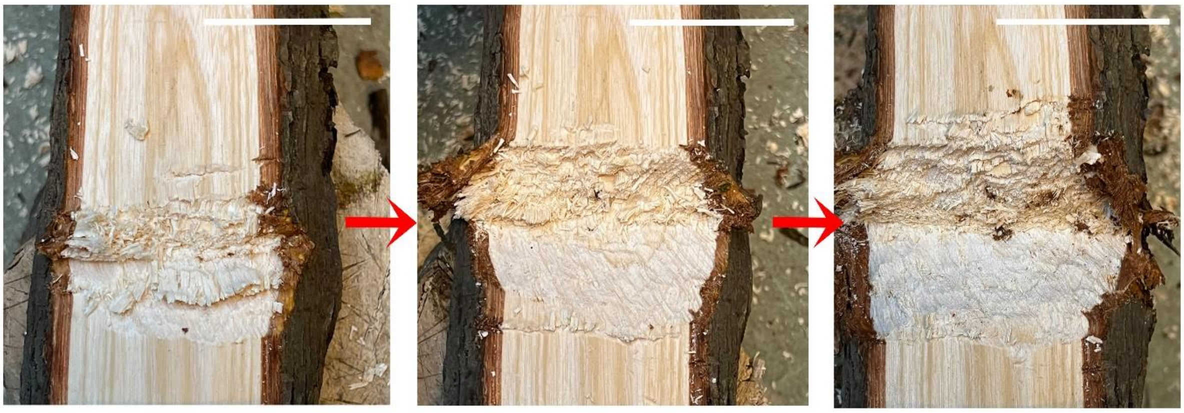

Figure 7.11. From left to right: gradual progress of cut during chopping.

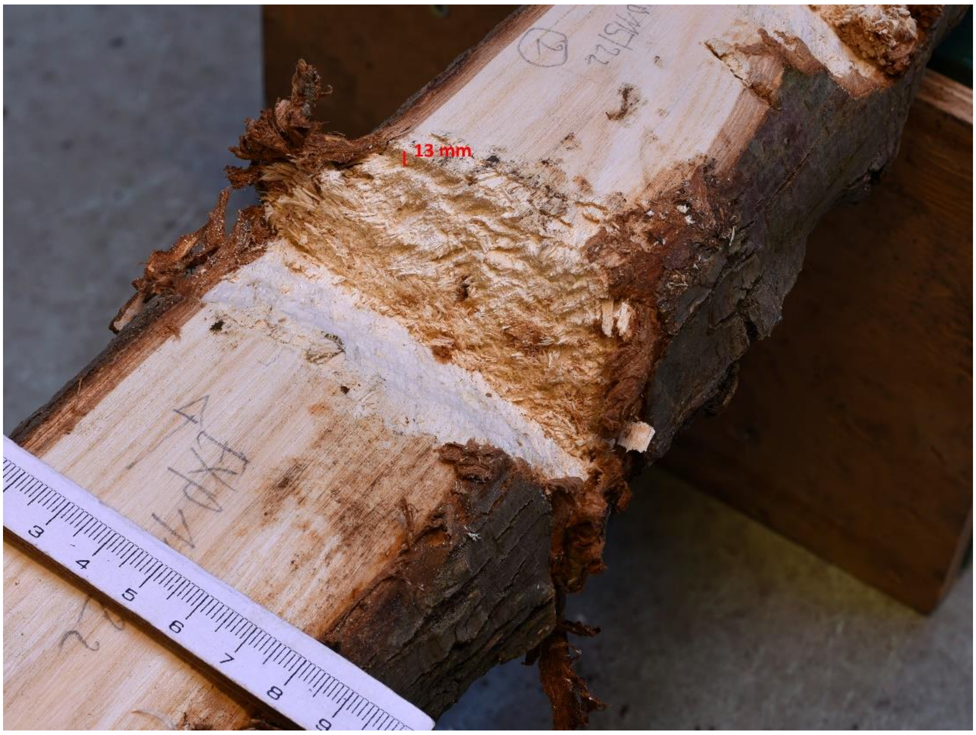

Figure 7.12. Stepwise incision, with indication of the depth (13 mm) of one of the first steps.

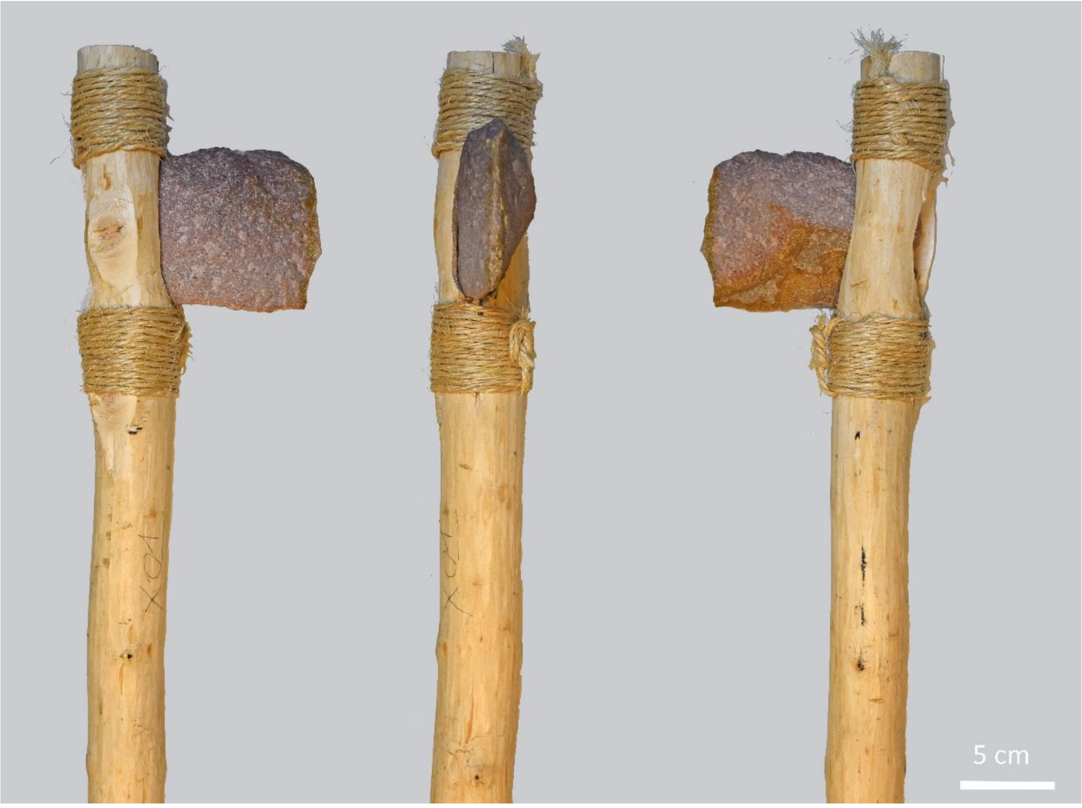

Figure 7.13. Hafted quartzite cleaver after 20 minutes of use.

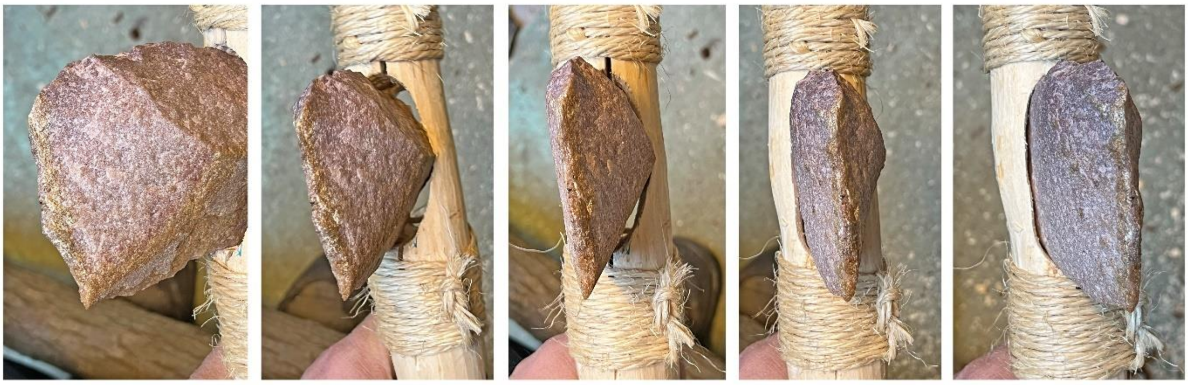

Figure 7.14. Detail of the used edge.

8. Ethnographic observations of woodworking, northern Zambia

Recording the wood-working knowledge of a traditional Bemba craftsman, Mr John Mukopa (age 80), was the focus of ethnographic fieldwork undertaken in July and August 2022 at his village in the Mungwi District, Northern Zambia. The village is situated in dryland forest known as *miombo* and dominated by tree species from three genera, *Brachestygia*, *Julbernardia* and *Isoberlina* (Smith and Allen 2004). Miombo woodland occurs across southern, central, and eastern Africa – from Angola to Mozambique - including the area around Kalambo Falls. The wood species identified at Site BLB are found locally in riverine settings and in miombo woodlands.

Mr Mukopa makes bark cloth, wooden drums, stools, and the handles for axes, adzes, mallets, and chisels and knives (Fig. 8.1). The working edges of his tools are iron inserts excepting mallets which have wood inserts for bark cloth making. Iron tools are known to produce facets on wood that are longer, wider and flatter than those made using chipped stone and ground axes (Coles and Orme 1985; López-Bultó et al. 2012; Sands 2016; Bamforth et al. 2018). The steel knife used in the taphonomic experiment (above) produced large smooth flat facets compared with the small concave facets left by the bifacial and unifacial stone tools. Iron axe edges also tend to get jammed into wood more frequently than thicker edged stone adzes and axes (Bamforth et al 2018). Despite the lack of direct comparability between iron and stone tool marks, the tools used by Mr Mukopa for felling trees and working roundwood (branches, sticks) have some relevance for interpreting the kinds of tools that might have been used to shape the tree trunks in BLB5 (object 1033 and the underly trunk), and to square the ends of the upper object ('cut log') in BLB4. In the case of the latter, stepped facets are a feature of metal and stone axes (Sands 2016) which enables us to speculate on the possible use of hafted tools in the shaping of the upper object in BLB4. The experimental hafted cleaver (Supplementary Section 7) also created stepped facets, similar in morphology to those seen on ends 'a' and 'b' of the BLB4 'cut log' (Extended Data Figure 8).

An axe is essential for felling trees and the preferred tool for removing large branches (Figure 8.2). Iron axe heads leave long deep chop marks when used vertically (Figure 8.3). Adzes are used for stripping bark and are the primary tool shaping wood (Figure 8.4). A long-bladed chisel, struck with a wood baton combined with a long, narrow axe blade is used to hollow a tree trunk (e.g., to make drums) (Figure 8.5). Felled trees serve as expedient work surfaces (Figure 8.3). A more permanent and stable work surface is made by flattening the base of a log and squaring the ends. A durable wood is chosen (*Pericopsis angolensis*) (Figure 8.6). Mr Mukopa's work surface was made in 1984 and is used for bark cloth making with a wood-headed mallet (Figures 8.6, 8.7) and for fine shaping work. The stepped squared ends resemble those of the BLB4 'cut log' (Extended Data Figure 8).

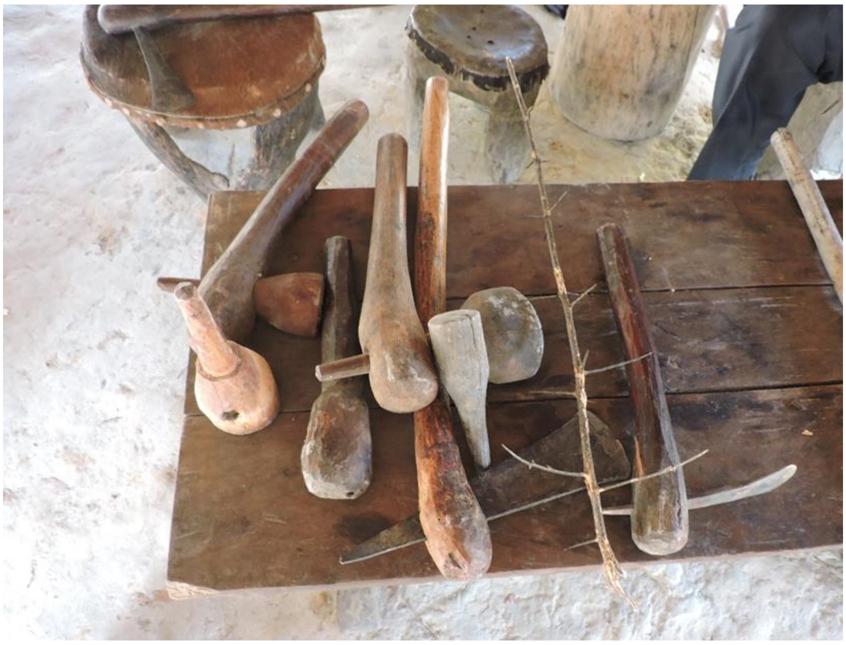

Figure 8.1. Tools made and used by Mr Mukopa. Adze (lower right), axe (lower central) and mallets (upper left). A loose mallet head is in the centre of the photo. The twig in the centre is a source of needles for sewing.

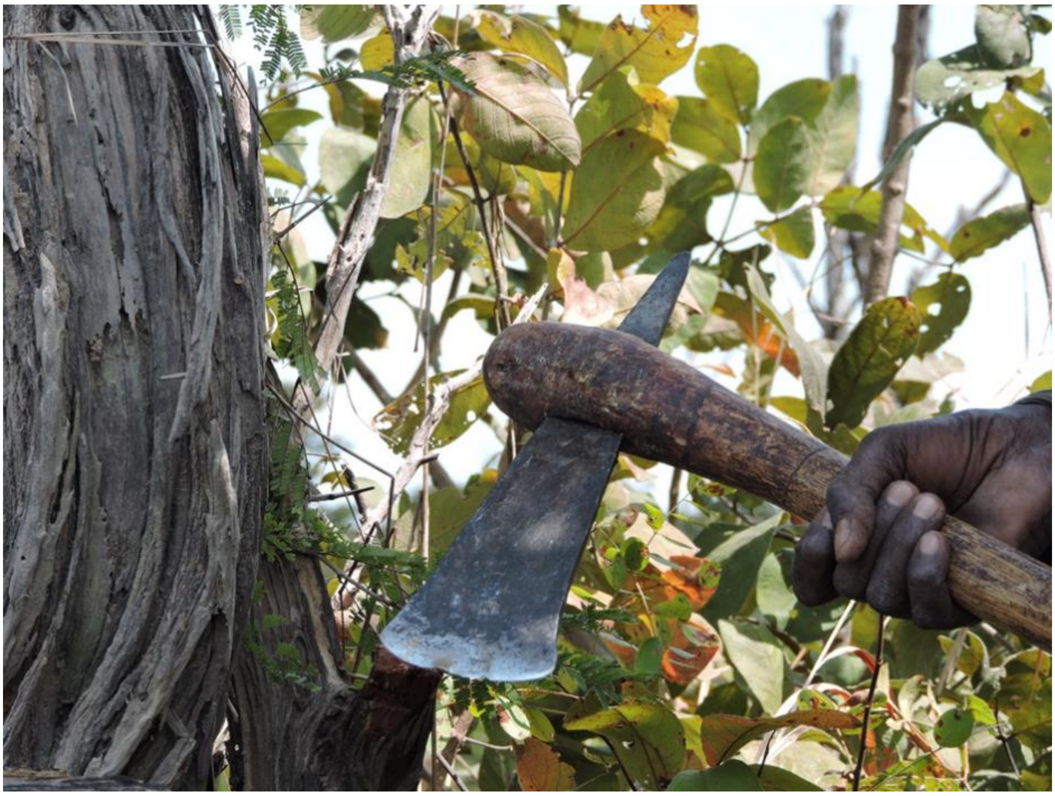

Figure 8.2. Hafted iron axe (socket arrangement) used to fell a medium size tree (10-12 m tall) *Pericopsis angolensis*). The axe blade is 8 cm wide and Mr Mukopa's large axes have cutting edges that range from 6-8 cm in width.

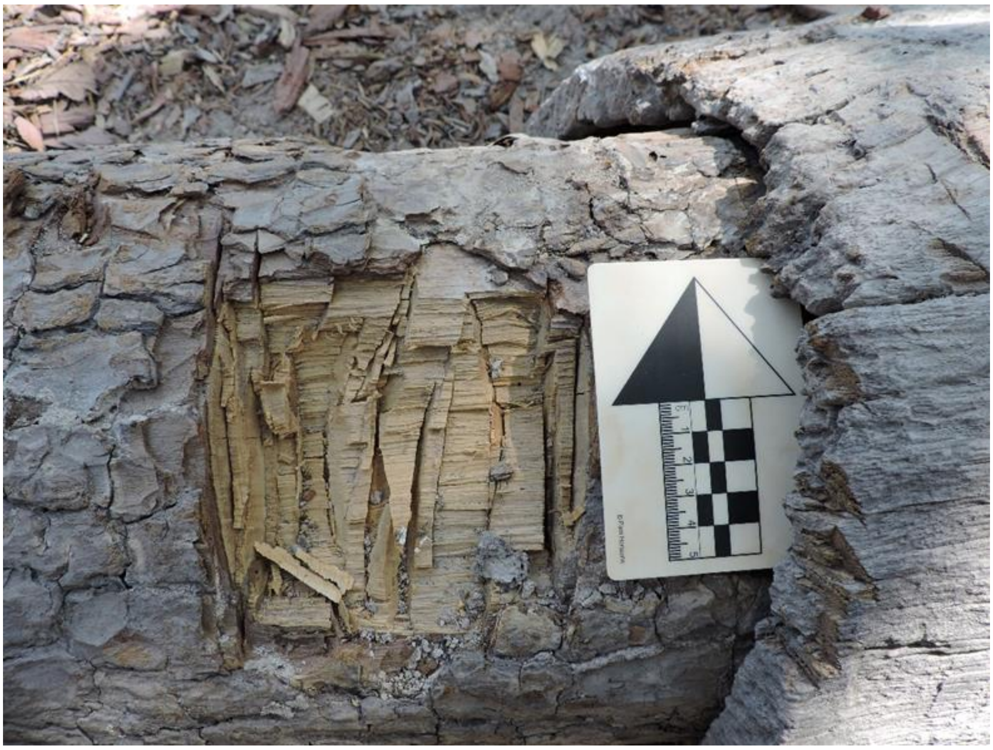

Figure 8.3. Iron axe chop marks on an expedient work surface (felled tree trunk).

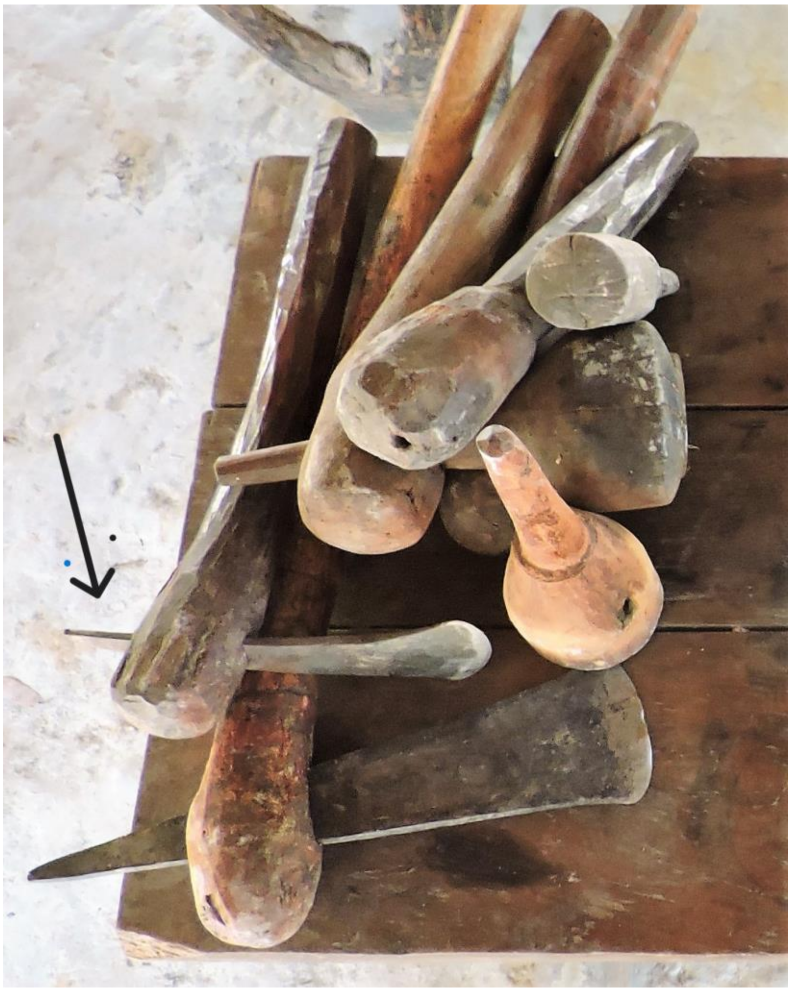

Figure 8.4. Set of tools for bark cloth making including a hafted adze blade (black arrow) overlying a hafted axe blade. The adze blade is perpendicular to the long axis of the handle and axe blade is parallel to the handle. Mr Mukopa's adze blades range in the width of the cutting edge from 2-4 cm. The adze is used for removing knots in wood, and fine shaping.

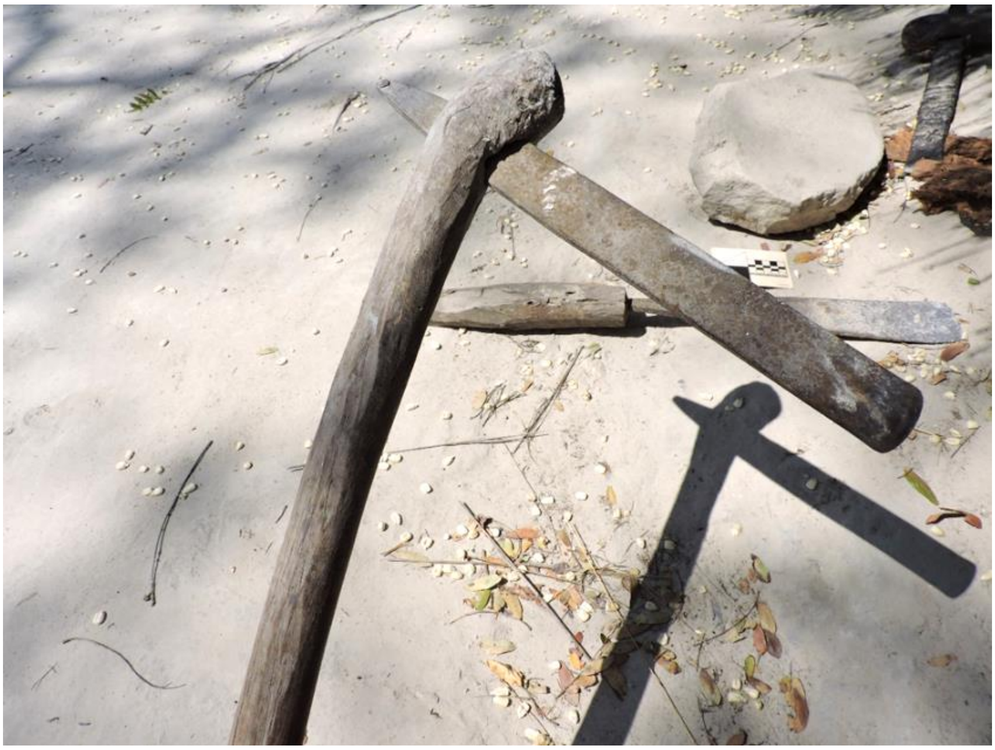

Figure 8.5. Long-bladed axe (foreground) and long-bladed chisel (background) for hollowing tree trunks. The quartzite block (rear, upper right) is a whetstone for sharpening axe and adze blades.

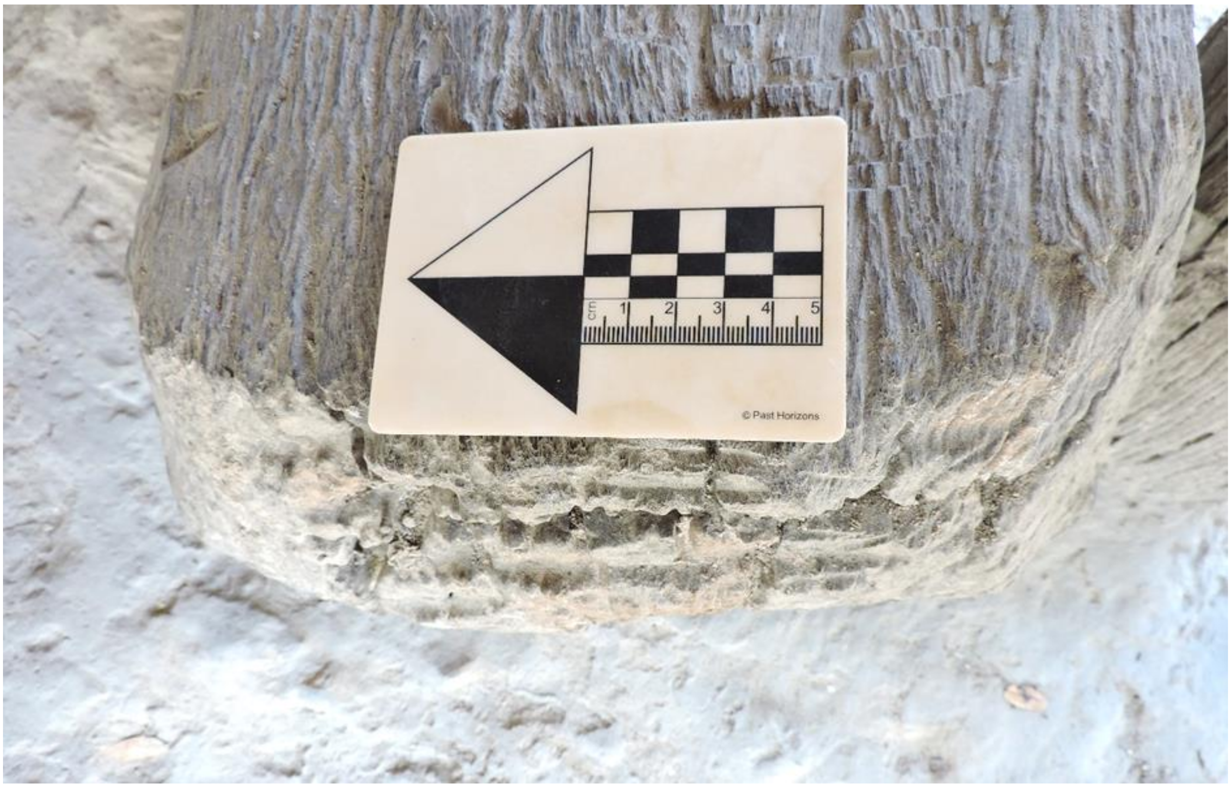

Figure 8.6. Work surface (debarked log) with squared ends. The stepped end is made using an axe, and the work surface has a slight lateral camber.

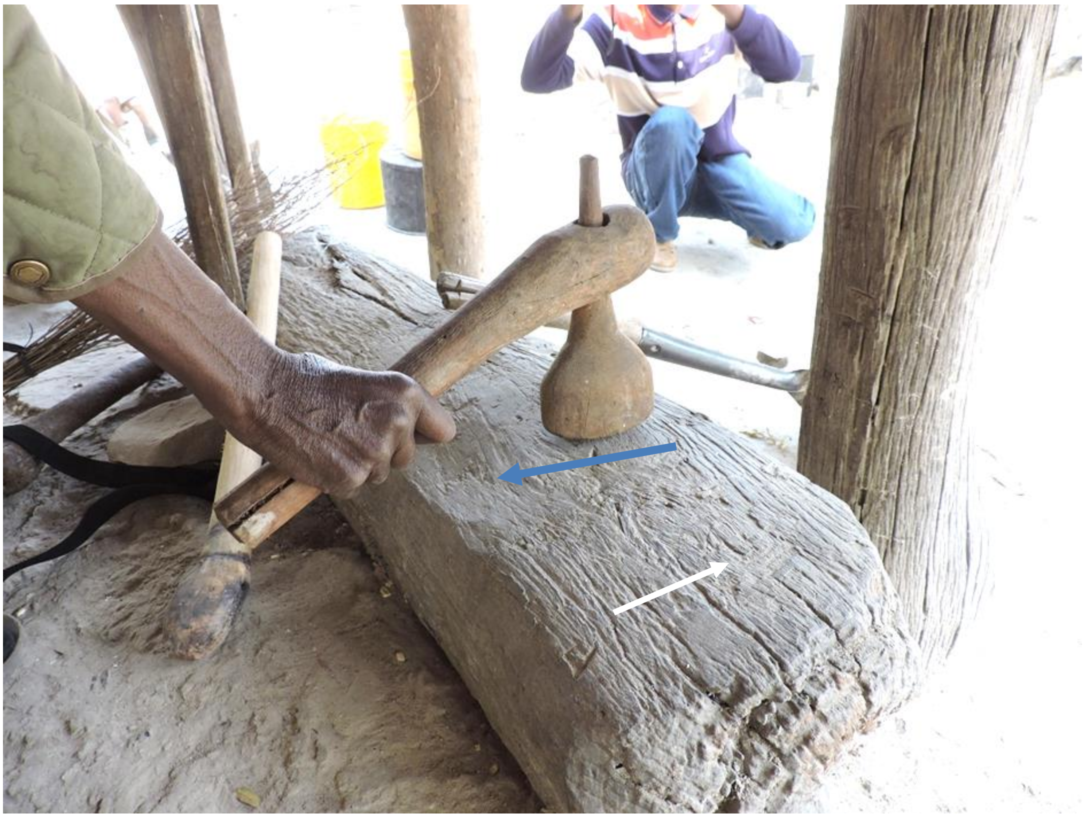

Figure 8.7. Permanent work surface showing detail of use history with random surface cut marks (white arrow) and what appears to be flattening and smoothing from long-term pounding with a mallet (blue arrow, below mallet head).

References:

Bamforth, M., et al. Woodworking technology. In Milner, N., Conneller, C. and Taylor, B. (eds.), *Star Carr Volume 2: Studies in Technology, Subsistence and Environment*. York: White Rose University Press. https://doi.org/10.22599/book2 (2018)

Coles, J.M., Orme, B.J. Prehistoric woodworking from the Somerset Levels: 3 Roundwood. In Coles, J.M., Orme, B.J. & Rouillard, S.E. (eds), Somerset Levels Papers, pp. 25-50. https://archaeologydataservice.ac.uk/library/browse/personDetails.xhtml?personId=20056 (1985)

López-Bultó, O., Piqué, R., Palomo, A. Woodworking technology and functional experimentation in the Neolithic site of La Draga (Banyoles, Spain). Exp. Archäol. Eur. - Bilanz 56–65. (2012)

Sands, R. *Prehistoric Woodworking*. Abingdon, Routledge (2016)

Smith, P. & Allen, Q. *Field Guide to the Trees and Shrubs of the Miombo Woodlands*. Kew: Royal Botanic Gardens (2004)
