# Supplementary material for: Evidence for the earliest structural use of wood at least 476,000 years ago
Source: Nature. 2023 Sep 20;622(7981):107–11. doi: 10.1038/s41586-023-06557-9 (PMC10550827; doi:10.1038/s41586-023-06557-9)
Supplement: Supplementary file 2 — Reporting Summary [file 41586_2023_6557_MOESM2_ESM.pdf]

nature portfolio

Corresponding author(s): Lawrence Barham

Last updated by author(s): 23/06/2023

Reporting Summary

Nature Portfolio wishes to improve the reproducibility of the work that we publish. This form provides structure for consistency and transparency in reporting. For further information on Nature Portfolio policies, see our Editorial Policies and the Editorial Policy Checklist.

Statistics

For all statistical analyses, confirm that the following items are present in the figure legend, table legend, main text, or Methods section.

n/a	Confirmed	
☐	☒	The exact sample size (n) for each experimental group/condition, given as a discrete number and unit of measurement
☐	☒	A statement on whether measurements were taken from distinct samples or whether the same sample was measured repeatedly
☒	☐	The statistical test(s) used AND whether they are one- or two-sided<br>*Only common tests should be described solely by name; describe more complex techniques in the Methods section.*
☒	☐	A description of all covariates tested
☐	☒	A description of any assumptions or corrections, such as tests of normality and adjustment for multiple comparisons
☐	☒	A full description of the statistical parameters including central tendency (e.g. means) or other basic estimates (e.g. regression coefficient) AND variation (e.g. standard deviation) or associated estimates of uncertainty (e.g. confidence intervals)
☒	☐	For null hypothesis testing, the test statistic (e.g. F, t, r) with confidence intervals, effect sizes, degrees of freedom and P value noted<br>*Give P values as exact values whenever suitable.*
☒	☐	For Bayesian analysis, information on the choice of priors and Markov chain Monte Carlo settings
☒	☐	For hierarchical and complex designs, identification of the appropriate level for tests and full reporting of outcomes
☒	☐	Estimates of effect sizes (e.g. Cohen's d, Pearson's r), indicating how they were calculated

Our web collection on statistics for biologists contains articles on many of the points above.

Software and code

Policy information about availability of computer code

Data collection	Reflectance transformation imaging (RTI) models of the wood objects were built using RTIbuilder 2.0.2, HSH highlight based fitter 1.0.1, and viewed with RTI viewer 1.1.0. (www.Culturalheritageimaging.org). Measurements from RTI models were taken with imageJ (version 1.53t) TPsDig2 version 2.31. GIMP 2.10 was used for post processing RTI images.<br>Photographs of the wood were taken in raw format and converted to JPEG using Adobe Lightroom. 3D photogrammetry models were constructed using Agisoft Metashape (1.8.1, build 13915, 64 bit) selecting the highest accuracy settings. 3D models were refitted and rotated for imaging as .stl files using MorphoDig (version 1.6.7 64 bit).
Data analysis	Opus 7.2 software was used to process the infrared spectra of each waterlogged wood sample.

For manuscripts utilizing custom algorithms or software that are central to the research but not yet described in published literature, software must be made available to editors and reviewers. We strongly encourage code deposition in a community repository (e.g. GitHub). See the Nature Portfolio guidelines for submitting code & software for further information.

Data

Policy information about availability of data

All manuscripts must include a data availability statement. This statement should provide the following information, where applicable:

- Accession codes, unique identifiers, or web links for publicly available datasets
- A description of any restrictions on data availability
- For clinical datasets or third party data, please ensure that the statement adheres to our policy

Photographic data (wood objects, RTI models, and 3D photogrammetry models) freely available (open access) at 10.17638/datacat.liverpool.ac.uk/2200

Research involving human participants, their data, or biological material

Policy information about studies with human participants or human data. See also policy information about sex, gender (identity/presentation), and sexual orientation and race, ethnicity and racism.

Reporting on sex and gender	Ethnographic research (Supplementary Section 8) was undertaken in Zambia with a single participant (self-reported as biological male, aged 80) with full informed consent given.
Reporting on race, ethnicity, or other socially relevant groupings	Ethnicity self-reported as Bemba (linguistic identification).
Population characteristics	The study is based on a single individual.
Recruitment	The individual was selected as one of the last three remaining traditional wood-working craftspeople in northern Zambia. Informed consent was obtained and ethics approval was obtained from the University of Zambia (HSSREC-20220-APR-022) and the University of Liverpool Central Univeristy Ethics Comitteees (REF: 11408).
Ethics oversight	Ethics approval was obtained from the University of Zambia (HSSREC-20220-APR-022), the University of Liverpool Central University Ethics Committees (REF: 11408) and the British Museum (EMKP 2020SG01) with end of project reporting to these institutions.

Note that full information on the approval of the study protocol must also be provided in the manuscript.

Field-specific reporting

Please select the one below that is the best fit for your research. If you are not sure, read the appropriate sections before making your selection.

☐ Life sciences ☐ Behavioural & social sciences ☒ Ecological, evolutionary & environmental sciences

For a reference copy of the document with all sections, see nature.com/documents/nr-reporting-summary-flat.pdf

Life sciences study design

All studies must disclose on these points even when the disclosure is negative.

Sample size	*Describe how sample size was determined, detailing any statistical methods used to predetermine sample size OR if no sample-size calculation was performed, describe how sample sizes were chosen and provide a rationale for why these sample sizes are sufficient.*
Data exclusions	*Describe any data exclusions. If no data were excluded from the analyses, state so OR if data were excluded, describe the exclusions and the rationale behind them, indicating whether exclusion criteria were pre-established.*
Replication	*Describe the measures taken to verify the reproducibility of the experimental findings. If all attempts at replication were successful, confirm this OR if there are any findings that were not replicated or cannot be reproduced, note this and describe why.*
Randomization	*Describe how samples/organisms/participants were allocated into experimental groups. If allocation was not random, describe how covariates were controlled OR if this is not relevant to your study, explain why.*
Blinding	*Describe whether the investigators were blinded to group allocation during data collection and/or analysis. If blinding was not possible, describe why OR explain why blinding was not relevant to your study.*

Behavioural & social sciences study design

All studies must disclose on these points even when the disclosure is negative.

Study description	*Briefly describe the study type including whether data are quantitative, qualitative, or mixed-methods (e.g. qualitative cross-sectional, quantitative experimental, mixed-methods case study).*
Research sample	*State the research sample (e.g. Harvard university undergraduates, villagers in rural India) and provide relevant demographic information (e.g. age, sex) and indicate whether the sample is representative. Provide a rationale for the study sample chosen. For studies involving existing datasets, please describe the dataset and source.*
Sampling strategy	*Describe the sampling procedure (e.g. random, snowball, stratified, convenience). Describe the statistical methods that were used to predetermine sample size OR if no sample-size calculation was performed, describe how sample sizes were chosen and provide a rationale for why these sample sizes are sufficient. For qualitative data, please indicate whether data saturation was considered, and what criteria were used to decide that no further sampling was needed.*
Data collection	*Provide details about the data collection procedure, including the instruments or devices used to record the data (e.g. pen and paper, computer, eye tracker, video or audio equipment) whether anyone was present besides the participant(s) and the researcher, and whether the researcher was blind to experimental condition and/or the study hypothesis during data collection.*
Timing	*Indicate the start and stop dates of data collection. If there is a gap between collection periods, state the dates for each sample cohort.*
Data exclusions	*If no data were excluded from the analyses, state so OR if data were excluded, provide the exact number of exclusions and the rationale behind them, indicating whether exclusion criteria were pre-established.*
Non-participation	*State how many participants dropped out/declined participation and the reason(s) given OR provide response rate OR state that no participants dropped out/declined participation.*
Randomization	*If participants were not allocated into experimental groups, state so OR describe how participants were allocated to groups, and if allocation was not random, describe how covariates were controlled.*

Ecological, evolutionary & environmental sciences study design

All studies must disclose on these points even when the disclosure is negative.

Study description	Archaeological excavations took place at site BLB, Kalambo Falls, Zambia from 7 July to 4 August 2019. Five distinct areas were excavated, labeled BLB1, BLB2, BLB3, BLB4, and BLB5. BLB1 was a geological section through the full 9m depth of deposit.
Research sample	Five fossil wood objects were collected for analysis and 16 sediment samples for dating.
Sampling strategy	The placement of excavation units was designed to sample a stretch of exposed river bank for sampling sediments and recovery of archaeological and paleoenvironmental material. The deepest excavations (BLB3, BLB5) reached 1 m below the water table at which point the excavations were halted.
Data collection	All objects and sediment samples were recorded in three-dimensional space using a total station by the project team under the direction of PI Prof Barham. Excavators wore nitrile powder free gloves to minimise surface contact and all artefacts were doubled bagged and separate sediment sample included for residue analyses. Artefact bags numbered and logged. Photographs for photogrammetry taken and sections drawn.
Timing and spatial scale	Data collection at Kalambo Falls took place between 7 July and 4 August 2019 with wood objects recovered from four of the five excavation blocks. Ethnographic fieldwork took place in northern Zambia in July-August 2022. Analyses of the wood objects began in October 2019 at the University of Liverpool and was interrupted by Covid-19 from March 2020 until January 2021. Photographic analyses completed in October 2022. Infrared analyses of wood samples completed January 2023.
Data exclusions	No data were excluded.
Reproducibility	The data collection parameters for producing wood infrared spectra differed slightly between the analysis of the modern reference collection and the waterlogged wood and the modern reference sample. Royal Botanical Gardens, Kew (RBG) supplied a modern comparison sample of Combretum zeyheri with IR spectra from three separate areas on the wood. There was no preparation of the samples before analysis. Four scans were performed for each sample in the Mid-IR (4000-550 cm-1) and 32 in the Near-IR investigations (1000-4000 cm-1). Six samples were taken from each waterlogged wood specimens (object 219, 1033), and freeze dried before analysis. The spectra of each sample spectra of each sample was captured using 30 scans covering a wavelength range of 4000-500 cm-1.
Randomization	Infrared samples were collected within and beyond the target area of interest on object 1033 (BLB5) to identify patterning in the spectra. No randomisation was applied.
Blinding	Blinding was not necessary for the collection of infrared data. A modern reference sample of the wood species Combretum zeyheri

Blinding	was used as the comparator to the archaeological wood identified as Combretum zeyheri.

Did the study involve field work? ☒ Yes ☐ No

Field work, collection and transport

Field conditions	Exposed riverbank sediments, including waterlogged sediments below river level.
Location	08º 35' 38.96"South; 31º 14' 36.77", East, elevation 1390 amsl, river depth unknown
Access & import/export	Permission to excavate Site BLB, Kalambo Falls was granted by the National Heritage Conservation Commission as part of the Deep Roots Project (Permit dated 29 June 2017; PI Lawrence Barham, University of Liverpool). The right to temporary export was granted by the National Heritage Conservation Commission on the agreement that the wood objects would be conserved before their return to the Livingstone Museum, Zambia (Permit NHCC/2019/01/LB1). Access to the site is by track from the town of Mbala, 35km to the south. A footpath leaves the track toward the river and follows the river bank. Wood samples were collected from waterlogged sediments and from organic clays above the river. The samples were stored in waterfilled tanks on site before being wrapped in cling film, to retain moisture, and embedded in 60 mm foam to minimise shock on travel, and placed in purpose made marine plywood boxes. The crated samples were transported to Lusaka from where they were airfreighted to the University of Liverpool for storage in water filled tanks during the period of analysis. The samples are now being conversed by the York Archaeological Trust.
Disturbance	All excavation areas were backfilled to minimise disturbance to the deposits.

Reporting for specific materials, systems and methods

We require information from authors about some types of materials, experimental systems and methods used in many studies. Here, indicate whether each material, system or method listed is relevant to your study. If you are not sure if a list item applies to your research, read the appropriate section before selecting a response.

Materials & experimental systems

n/a	Involved in the study	
☒	☐	Antibodies
☒	☐	Eukaryotic cell lines
☐	☒	Palaeontology and archaeology
☒	☐	Animals and other organisms
☒	☐	Clinical data
☒	☐	Dual use research of concern
☒	☐	Plants

Methods

n/a	Involved in the study	
☒	☐	ChIP-seq
☒	☐	Flow cytometry
☒	☐	MRI-based neuroimaging

Antibodies

Antibodies used	*Describe all antibodies used in the study; as applicable, provide supplier name, catalog number, clone name, and lot number.*
Validation	*Describe the validation of each primary antibody for the species and application, noting any validation statements on the manufacturer's website, relevant citations, antibody profiles in online databases, or data provided in the manuscript.*

Eukaryotic cell lines

Policy information about cell lines and Sex and Gender in Research

Cell line source(s)	*State the source of each cell line used and the sex of all primary cell lines and cells derived from human participants or vertebrate models.*
Authentication	*Describe the authentication procedures for each cell line used OR declare that none of the cell lines used were authenticated.*
Mycoplasma contamination	*Confirm that all cell lines tested negative for mycoplasma contamination OR describe the results of the testing for mycoplasma contamination OR declare that the cell lines were not tested for mycoplasma contamination.*
Commonly misidentified lines (See ICLAC register)	*Name any commonly misidentified cell lines used in the study and provide a rationale for their use.*

Palaeontology and Archaeology

Specimen provenance	All specimens were collected from the site BLB, Kalambo Falls, Zambia (08º 35' 38.96"South; 31º 14' 36.77) with specimens identified by excavation area (BLB2, BLB3, BLB4, BLB5) and position recorded in x,y,z coordinates. Permission to excavate Site BLB was granted by the National Heritage Conservation Commission as part of the Deep Roots Project (Permit dated 29 June 2017; PI Lawrence Barham, University of Liverpool). The right to temporary export of the wood was granted by the National Heritage Conservation Commission on the agreement that the wood objects would be conserved before their return to the Livingstone Museum, Zambia (Permit NHCC/2019/01/LB1).
Specimen deposition	The specimens are currently being conserved by the York Archaeological Trust (York, UK) and on completion of conservation they will be deposited in the Livingstone Museum, Livingstone, Zambia.
Dating methods	Sixteen samples for luminescence dating were collected by hammering opaque plastic tubes into the sediment. The ends of the tubes were sealed in the field to avoid light exposure and to retain moisture, and then returned to the laboratory for analysis. Sediment from the inner part of the tubes was isolated and further processed for luminescence dating analyses. The samples were airfreighted from Zambia to Aberystwyth University, UK. All sample processing and analysis was undertaken at Aberystwyth Luminescence Research Laboratory in red light conditions. Prior to chemical treatments, the coarsest samples were wet-sieved to isolate grains ranging from 90-1000 µm, whilst other samples were immediately subjected to chemical reagents. Carbonates were removed by treating the samples with 10% HCl for at least 3 days or until no continued reaction was observed. Initial reactions with HCl were mild or unobservable for all samples. After rinsing three times with distilled water, the sediment was put in to 20 vol H2O2 to dissolve organic matter before the samples were again rinsed three times and dried. Samples were then dry sieved using a stack of sieves ranging from 90 to 355 µm. A combination of field gamma spectrometry, laboratory alpha and beta counting, and geochemical analyses were used to determine the radionuclide content and DRAC was used to calculate the radiation dose rate. Sand-sized grains (~150 to 250 µm in diameter) of quartz and potassium-rich feldspar were isolated under red-light conditions for luminescence measurements and measured on Risø TL/OSL instruments using single aliquot regenerative dose protocols. Single grain quartz optically stimulated luminescence (OSL) measurements were able to date sediments younger than ~60 ka; beyond this age the OSL signal was saturated. Post-infrared infrared stimulated luminescence (pIR-IRSL) measurements of aliquots consisting of ~50 grains of potassium-rich feldspars were able to provide ages for all samples collected. The pIR-IRSL signal yielded an average value for anomalous fading of 1.46 ± 0.50 % per decade. Where quartz OSL and feldspar pIR-IRSL were applied to the same samples, the ages were consistent within uncertainties without needing to correct for anomalous fading.

☒ Tick this box to confirm that the raw and calibrated dates are available in the paper or in Supplementary Information.

Ethics oversight	Ethics oversight was required on the collection of ethnographic observations as outlined above, and the research was approved by the University of Liverpool Central University Ethics Committees (no. 11408) the University of Zambia Human and Social Sciences Research Ethics Committee (HSREC-2022-APR-022,and the British Museum Endangered Material Knowledge Programm (2020SG01)

Note that full information on the approval of the study protocol must also be provided in the manuscript.

Animals and other research organisms

Policy information about studies involving animals; ARRIVE guidelines recommended for reporting animal research, and Sex and Gender in Research

Laboratory animals	*For laboratory animals, report species, strain and age OR state that the study did not involve laboratory animals.*
Wild animals	*Provide details on animals observed in or captured in the field; report species and age where possible. Describe how animals were caught and transported and what happened to captive animals after the study (if killed, explain why and describe method; if released, say where and when) OR state that the study did not involve wild animals.*
Reporting on sex	*Indicate if findings apply to only one sex; describe whether sex was considered in study design, methods used for assigning sex. Provide data disaggregated for sex where this information has been collected in the source data as appropriate; provide overall numbers in this Reporting Summary. Please state if this information has not been collected. Report sex-based analyses where performed, justify reasons for lack of sex-based analysis.*
Field-collected samples	*For laboratory work with field-collected samples, describe all relevant parameters such as housing, maintenance, temperature, photoperiod and end-of-experiment protocol OR state that the study did not involve samples collected from the field.*
Ethics oversight	*Identify the organization(s) that approved or provided guidance on the study protocol, OR state that no ethical approval or guidance was required and explain why not.*

Note that full information on the approval of the study protocol must also be provided in the manuscript.

Clinical data

Policy information about clinical studies

All manuscripts should comply with the ICMJE guidelines for publication of clinical research and a completed CONSORT checklist must be included with all submissions.

Clinical trial registration	*Provide the trial registration number from ClinicalTrials.gov or an equivalent agency.*

Study protocol	*Note where the full trial protocol can be accessed OR if not available, explain why.*
Data collection	*Describe the settings and locales of data collection, noting the time periods of recruitment and data collection.*
Outcomes	*Describe how you pre-defined primary and secondary outcome measures and how you assessed these measures.*

Dual use research of concern

Policy information about dual use research of concern

Hazards

Could the accidental, deliberate or reckless misuse of agents or technologies generated in the work, or the application of information presented in the manuscript, pose a threat to:

No	Yes	
☐	☐	Public health
☐	☐	National security
☐	☐	Crops and/or livestock
☐	☐	Ecosystems
☐	☐	Any other significant area

Experiments of concern

Does the work involve any of these experiments of concern:

No	Yes	
☐	☐	Demonstrate how to render a vaccine ineffective
☐	☐	Confer resistance to therapeutically useful antibiotics or antiviral agents
☐	☐	Enhance the virulence of a pathogen or render a nonpathogen virulent
☐	☐	Increase transmissibility of a pathogen
☐	☐	Alter the host range of a pathogen
☐	☐	Enable evasion of diagnostic/detection modalities
☐	☐	Enable the weaponization of a biological agent or toxin
☐	☐	Any other potentially harmful combination of experiments and agents

Plants

Seed stocks	*Report on the source of all seed stocks or other plant material used. If applicable, state the seed stock centre and catalogue number. If plant specimens were collected from the field, describe the collection location, date and sampling procedures.*
Novel plant genotypes	*Describe the methods by which all novel plant genotypes were produced. This includes those generated by transgenic approaches, gene editing, chemical/radiation-based mutagenesis and hybridization. For transgenic lines, describe the transformation method, the number of independent lines analyzed and the generation upon which experiments were performed. For gene-edited lines, describe the editor used, the endogenous sequence targeted for editing, the targeting guide RNA sequence (if applicable) and how the editor was applied.*
Authentication	*Describe any authentication procedures for each seed stock used or novel genotype generated. Describe any experiments used to assess the effect of a mutation and, where applicable, how potential secondary effects (e.g. second site T-DNA insertions, mosiacism, off-target gene editing) were examined.*

ChIP-seq

Data deposition

☐ Confirm that both raw and final processed data have been deposited in a public database such as GEO.

☐ Confirm that you have deposited or provided access to graph files (e.g. BED files) for the called peaks.

Data access links<br>*May remain private before publication.*	*For "Initial submission" or "Revised version" documents, provide reviewer access links. For your "Final submission" document, provide a link to the deposited data.*
Files in database submission	*Provide a list of all files available in the database submission.*

Genome browser session
(e.g. UCSC)

Provide a link to an anonymized genome browser session for "Initial submission" and "Revised version" documents only, to enable peer review. Write "no longer applicable" for "Final submission" documents.

Methodology

Replicates

Describe the experimental replicates, specifying number, type and replicate agreement.

Sequencing depth

Describe the sequencing depth for each experiment, providing the total number of reads, uniquely mapped reads, length of reads and whether they were paired- or single-end.

Antibodies

Describe the antibodies used for the ChIP-seq experiments; as applicable, provide supplier name, catalog number, clone name, and lot number.

Peak calling parameters

Specify the command line program and parameters used for read mapping and peak calling, including the ChIP, control and index files used.

Data quality

Describe the methods used to ensure data quality in full detail, including how many peaks are at FDR 5% and above 5-fold enrichment.

Software

Describe the software used to collect and analyze the ChIP-seq data. For custom code that has been deposited into a community repository, provide accession details.

Flow Cytometry

Plots

Confirm that:

☐ The axis labels state the marker and fluorochrome used (e.g. CD4-FITC).

☐ The axis scales are clearly visible. Include numbers along axes only for bottom left plot of group (a 'group' is an analysis of identical markers).

☐ All plots are contour plots with outliers or pseudocolor plots.

☐ A numerical value for number of cells or percentage (with statistics) is provided.

Methodology

Sample preparation

Describe the sample preparation, detailing the biological source of the cells and any tissue processing steps used.

Instrument

Identify the instrument used for data collection, specifying make and model number.

Software

Describe the software used to collect and analyze the flow cytometry data. For custom code that has been deposited into a community repository, provide accession details.

Cell population abundance

Describe the abundance of the relevant cell populations within post-sort fractions, providing details on the purity of the samples and how it was determined.

Gating strategy

Describe the gating strategy used for all relevant experiments, specifying the preliminary FSC/SSC gates of the starting cell population, indicating where boundaries between "positive" and "negative" staining cell populations are defined.

☐ Tick this box to confirm that a figure exemplifying the gating strategy is provided in the Supplementary Information.

Magnetic resonance imaging

Experimental design

Design type

Indicate task or resting state; event-related or block design.

Design specifications

Specify the number of blocks, trials or experimental units per session and/or subject, and specify the length of each trial or block (if trials are blocked) and interval between trials.

Behavioral performance measures

State number and/or type of variables recorded (e.g. correct button press, response time) and what statistics were used to establish that the subjects were performing the task as expected (e.g. mean, range, and/or standard deviation across subjects).

Acquisition

Imaging type(s) | *Specify: functional, structural, diffusion, perfusion.*

Field strength | *Specify in Tesla*

Sequence & imaging parameters | *Specify the pulse sequence type (gradient echo, spin echo, etc.), imaging type (EPI, spiral, etc.), field of view, matrix size, slice thickness, orientation and TE/TR/flip angle.*

Area of acquisition | *State whether a whole brain scan was used OR define the area of acquisition, describing how the region was determined.*

Diffusion MRI ☐ Used ☐ Not used

Preprocessing

Preprocessing software | *Provide detail on software version and revision number and on specific parameters (model/functions, brain extraction, segmentation, smoothing kernel size, etc.).*

Normalization | *If data were normalized/standardized, describe the approach(es): specify linear or non-linear and define image types used for transformation OR indicate that data were not normalized and explain rationale for lack of normalization.*

Normalization template | *Describe the template used for normalization/transformation, specifying subject space or group standardized space (e.g. original Talairach, MNI305, ICBM152) OR indicate that the data were not normalized.*

Noise and artifact removal | *Describe your procedure(s) for artifact and structured noise removal, specifying motion parameters, tissue signals and physiological signals (heart rate, respiration).*

Volume censoring | *Define your software and/or method and criteria for volume censoring, and state the extent of such censoring.*

Statistical modeling & inference

Model type and settings | *Specify type (mass univariate, multivariate, RSA, predictive, etc.) and describe essential details of the model at the first and second levels (e.g. fixed, random or mixed effects; drift or auto-correlation).*

Effect(s) tested | *Define precise effect in terms of the task or stimulus conditions instead of psychological concepts and indicate whether ANOVA or factorial designs were used.*

Specify type of analysis: ☐ Whole brain ☐ ROI-based ☐ Both

Statistic type for inference | *Specify voxel-wise or cluster-wise and report all relevant parameters for cluster-wise methods.*

(See Eklund et al. 2016)

Correction | *Describe the type of correction and how it is obtained for multiple comparisons (e.g. FWE, FDR, permutation or Monte Carlo).*

Models & analysis

n/a	Involved in the study
☐	☐ Functional and/or effective connectivity
☐	☐ Graph analysis
☐	☐ Multivariate modeling or predictive analysis

Functional and/or effective connectivity | *Report the measures of dependence used and the model details (e.g. Pearson correlation, partial correlation, mutual information).*

Graph analysis | *Report the dependent variable and connectivity measure, specifying weighted graph or binarized graph, subject- or group-level, and the global and/or node summaries used (e.g. clustering coefficient, efficiency, etc.).*

Multivariate modeling and predictive analysis | *Specify independent variables, features extraction and dimension reduction, model, training and evaluation metrics.*
